# The Life of a Kidney Podocyte

**DOI:** 10.1111/apha.70081

**Published:** 2025-07-23

**Authors:** Desiree Loreth, Wiebke Sachs, Catherine Meyer‐Schwesinger

**Affiliations:** ^1^ Institute of Cellular and Integrative Physiology University Hospital Hamburg‐Eppendorf Hamburg Germany; ^2^ Hamburg Center for Kidney Health University Hospital Hamburg‐Eppendorf Hamburg Germany

**Keywords:** actin cytoskeleton, degradation, foot process, metabolism, physiology, podocyte, polarity, regeneration, slit diaphragm

## Abstract

**Aim:**

Podocytes, highly specialized epithelial cells located in the glomerulus of the kidney, are essential to the filtration barrier that ensures separation of blood and urine. These cells exhibit a unique architecture, characterized by an intricate network of foot processes interconnected by slit diaphragms, which serve as a critical selective filter for plasma ultrafiltration.

This review focusses on synthesizing current knowledge on podocyte physiology, emphasizing the roles of key proteins, signaling pathways, and environmental factors that influence their function.

**Methods:**

Publications featuring current advances in molecular biology and imaging techniques were used to summarize new insights into the regulatory pathways governing podocyte homeostasis, as well as the mechanisms of injury and repair.

**Results:**

The biology of podocytes encompasses diverse processes, including cytoskeletal dynamics, cellular signaling, and interactions with neighboring cells and the extracellular matrix. Disruption of podocyte structure or function is fundamental to a variety of glomerular diseases, which can lead to proteinuria and progressive kidney failure.

**Conclusion:**

Understanding the intricate mechanisms involved in maintaining podocyte homeostasis offers potential therapeutic strategies to protect and restore podocyte integrity, addressing a critical need in nephrology. By highlighting the intricate balance required for podocyte survival, we reinforce their significance as both a cornerstone of renal filtration and a focal point in kidney disease research.

## 1 Introduction on the Importance of Podocytes

**FIGURE 1:**
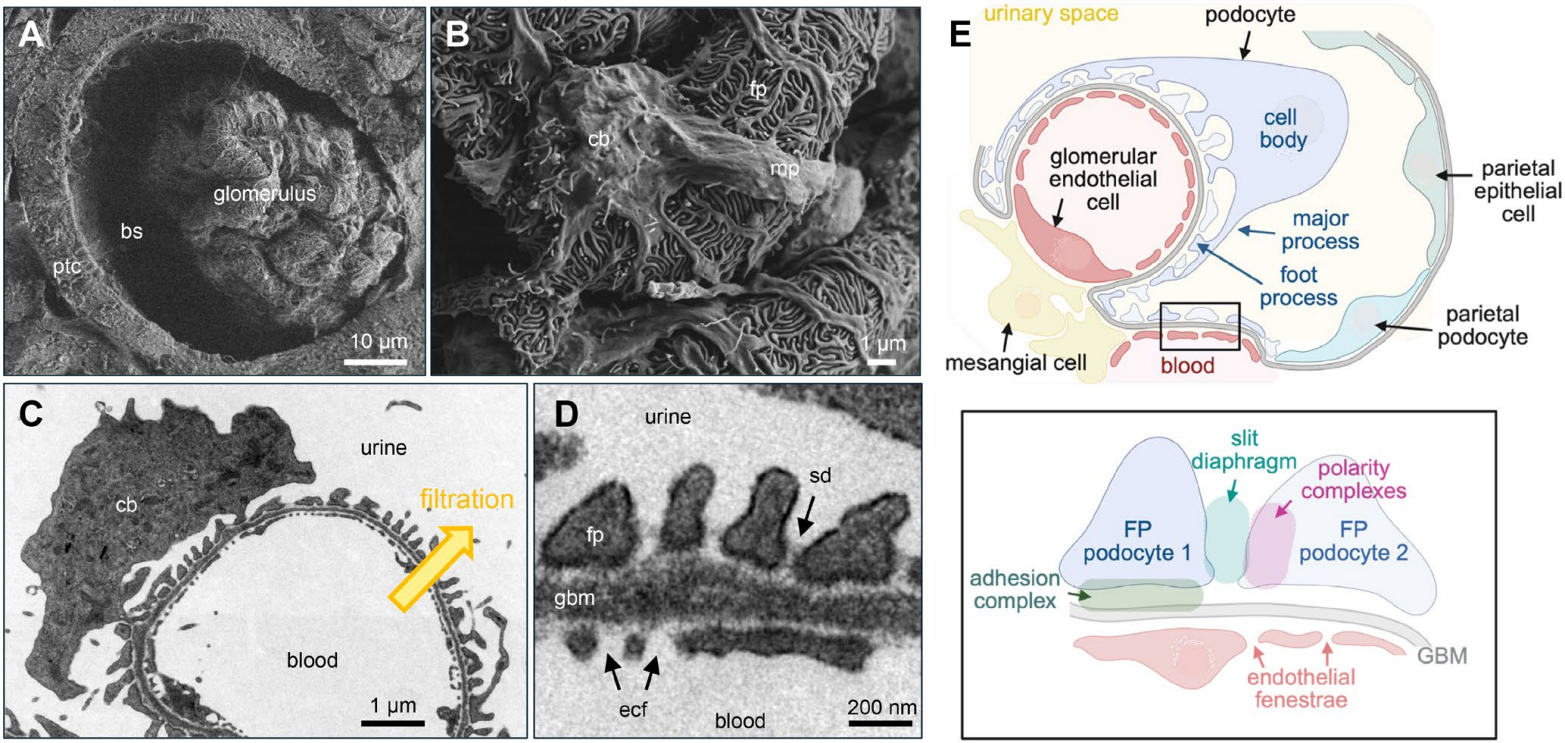
Overview of podocyte localization and cellular organization. (A, B) Scanning electron micrograph of a mouse glomerulus; bs = Bowman's space; cb = cell body; fp = foot processes; mp = major processes; ptc = proximal tubular cell. (C, D) Transmission electron micrographs of a glomerular capillary loop and the filtration barrier; ecf = endothelial cell fenestration; gbm = glomerular basement membrane; sd = slit diaphragm. (E) Podocytes reside within the urinary space and envelope the glomerular capillaries with an intricate network of interdigitating major‐ and foot processes (FP). Foot process proteins can be organized into functional domains, such as the slit diaphragm, and adhesion and polarity complexes. SEM and TEM photos : Courtesy: Lars Fester.

The kidney assures adequate blood filtration and urine production within the glomerulus by the functional interplay of three resident glomerular cell types, namely visceral epithelial cells (podocytes), glomerular endothelial cells (GEnCs), and mesangial cells (Figure 1). The glomerular filtration barrier (GFB) imparts both size‐selective and charge‐selective properties and is composed of podocytes from the urinary side and GEnCs from the blood side. Both cell types are separated by the glomerular basement membrane (GBM). Mesangial cells anchor the glomerular capillaries to the vascular stalk to provide structural support. As specialized pericytes, mesangial cells indirectly participate in filtration by reducing the glomerular surface area by contraction and are also thought to participate in matrix turnover and innate immune function [1]. Parietal epithelial cells (PECs) cover the inner aspect of Bowman's capsule and, besides GEnCs, podocytes, and mesangial cells, constitute the fourth resident cell type of the kidney glomerulus. In the mature kidney, PECs are a heterogeneous population of cells, as PECs at the urinary pole maintain features of proximal tubular cells and PECs at the vascular pole maintain features of podocytes and are therefore termed parietal podocytes [2].

While all glomerular cells form a functional and integrated syncytium, podocytes have been center stage of research in the last 25 years due to their fundamental importance for filtration barrier development and function, imparting both maintenance and selectivity of filtration. Podocytes regulate glomerular filtration [3] by presumably compressing the GBM through their adhesion to the GBM and through the tensile forces of their cytoskeleton, which in turn reduces the permeability of the filtration barrier to macromolecules [4]. Furthermore, podocytes regulate glomerular filtration through the formation of the slit diaphragm, a specialized cell–cell junction, and by sensing the glomerular filtration pressure by a mechanoreceptor complex situated at the slit diaphragm [5]. Podocytes have also reached center stage of research due to the fact that podocyte injury represents a major independent risk factor not only for a wide variety of kidney diseases [6] but also for cardiovascular mortality [7]. Podocytes are exposed to mechanical stress from the pulsating glomerular capillaries and shear stress from the permanent filtration of large volumes. Additionally, due to their localization in Bowman's urinary space, podocytes are also exposed to multiple metabolic, inflammatory, toxic, and osmotic stressors resulting in podocyte injury. The hallmark of podocyte disease is loss of protein into the urine (proteinuria), especially albumin (albuminuria), which is an early indicator. Thus, understanding podocyte physiology has major clinical and health economic significance and represents a prerequisite for the development of pathophysiologic concepts of podocyte disease.

This review aims to provide a broad introduction to the podocyte field for a large readership, especially for young researchers. Updating the 2003 review on the “Cell biology of the glomerular podocyte” by H. Pavenstädt, W. Kriz, and M. Kretzler [8], this review will focus on summarizing our latest advancements in deciphering the basic cell physiology of podocytes from birth to death. In the following paragraphs we will cover the main aspects of podocyte structure and molecular specialties, highlighting (1) proteins with podocyte‐restricted expression within the kidney, some of which were identified in the last 2 decades due to their role in kidney autoimmune injury and (2) proteins podocytes share with neurons as an example as to how comparative analyses can broaden our understanding of podocytes. We address the principles of podocyte development and of their terminal differentiation. We will summarize experimental data that demonstrate how many podocytes we have and what regenerative capacity they might have, as podocyte loss is one major determinant of chronic kidney disease. The physiologic functions and motility of podocytes at the filtration barrier and their involvement in the immune system will be synthesized. The communication of podocytes with the other resident glomerular cells, which is thought to not only assure glomerular health but also to contribute to glomerular demise in the setting of podocyte injury will be addressed. Our current view on the principles of the podocyte metabolism, protein degradation and waste removal, as well as membrane protein proteolysis will be summarized as new emerging research fields. Stressors affecting podocytes and what is known about their subsequent death paths will be briefly summarized. Lastly, to finalize this review, we will provide a concise overview of the main experimental models developed over the decades to study podocyte biology, as these provide the foundation of our current knowledge about this intricate cell type. Some basic podocyte functions will appear in more than one highlighted aspect of podocyte physiology. This review is based on many excellent expert reviews on podocyte biology. Importantly, we apologize to those many researchers, whose work could not be cited.

## 2 Podocyte Structure and Molecular Specialities

### 2.1 Overview

**FIGURE 2:**
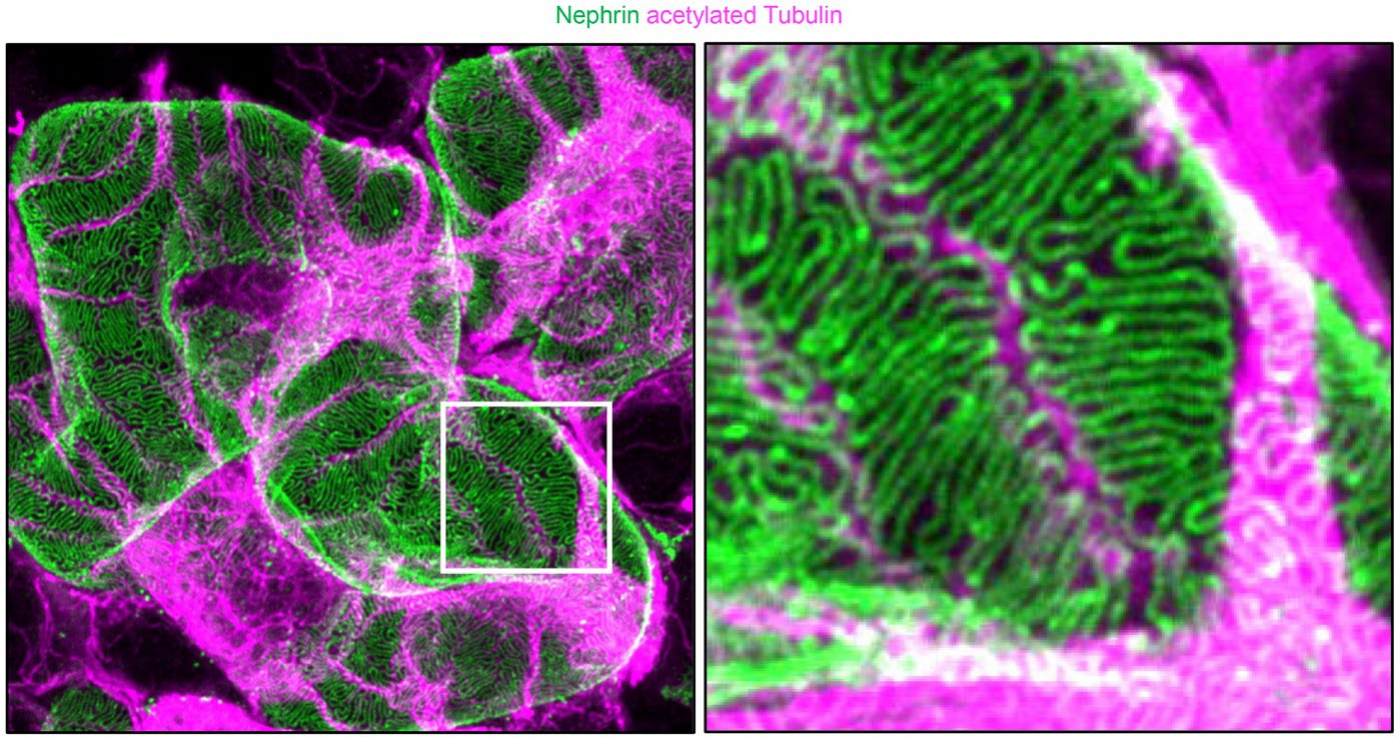
Super‐resolution fluorescence microscopy of podocytes in a cleared mouse kidney. Podocyte processes are not randomly organized on glomerular capillaries but show a preferred orientation with secondary foot processes being aligned in parallel with the axis of orientation (longitudinal axis). The slit diaphragm protein nephrin is stained in green and acetylated tubulin, which demarcates the podocyte cell body and primary processes, in magenta. Micrographs courtesy: David Unnersjö‐Jess.

Podocytes are highly differentiated, mesenchymal‐derived cells [9], which are intriguing because of their unique morphology and function. They are highly polarized cells with an elaborate cytoskeleton. The apico‐basal polarity axis (see Section 2.3) allows for podocyte orientation between the urinary space and the glomerular basement membrane (GBM) [10]. Podocytes reside in the urinary space and embrace the glomerular capillaries with their flat cell body, from which they extend 5 to 10 long branching major (primary) processes. Major processes give rise to numerous secondary processes (often called and commonly known as “foot processes”), which interdigitate in a zipper‐like fashion with foot processes of neighboring podocytes [11]. The newly described ridge‐like prominences, which are formed at the basal surface of the cell body and major processes, serve as an adhesion apparatus for the attachment of the cell body and major processes to the GBM and as a connecting apparatus of peripheral foot processes to the cell body and major processes [12]. Foot processes attach podocytes to the underlying extracellular matrix (ECM) of the GBM by an adhesion complex, which is composed of specific proteins such as adhesion receptors [13] (see Section 2.4). Foot processes are interconnected by highly sophisticated cell–cell contacts that combine components of several types of cell–cell junctions, including tight, adhesion, neuronal, and gap junctions and are called “slit diaphragms” [14, 15] (see Section 2.2). In general, interdigitating foot processes of the mature podocyte are connected by bicellular junctions; however, areas of tricellular junctions (connection between foot processes from 3 different podocytes) have been described [12]. It is thought that tricellular junctions express slightly different junctional proteins than bicellular junctions in podocytes. In other epithelial cells, tricellular junctions contain proteins such as tricellulin (also known as MARVELD2) transmembrane protein [16] and angulin family transmembrane proteins [17, 18]. In podocytes, the angulin ILDR2 interacts with the junctional protein claudin 5. Even though ILDR2 is strongly upregulated in podocyte injury, mice deficient of ILDR2 do not develop a foot process phenotype [19]. Mature foot processes are uniform in width [12]. Deep learning‐based approaches for segmentation and quantitative analysis of foot processes in images acquired with confocal and super‐resolution fluorescence microscopy of cleared kidneys (Figure 2) now allow a detailed analysis of foot process morphology and organization [20, 21, 22], which represent the basis for mathematical modeling of mechanistic principles of the filtration barrier, such as its permeability [23]. Using this method, it was recently shown that podocyte processes are not randomly organized on glomerular capillaries but show a preferred orientation with foot processes being aligned in parallel with the axis of orientation (longitudinal axis). The authors speculate that this could be a result of mechanical cues originating from the different magnitudes of wall stress in the circumferential/axial dimensions of capillaries [24]. Further, detailed serial block‐face SEM imaging that enables 3D cellular ultrastructure analyses of the mature podocyte have identified new morphological aspects of podocyte processes, such as ridge‐like prominences, foot processes that derive from the cell body, and major process arcades. Major process arcades originate from the same podocyte when the distal portions of two major processes are anastomosed [12].

The apical surfaces of podocytes are covered by the surface sialomucin podocalyxin [25], whose highly negative charge functions to keep adjacent foot processes separated, thereby keeping the urinary filtration barrier open [26]. The importance of the podocytes' architecture is emphasized by the fact that among the 50+ known gene defects leading to inherited forms of podocyte diseases are a large number of genes encoding cytoskeleton‐ or slit diaphragm‐associated proteins [27] (see Sections 2.5, 2.7). A stereotypical response of the stressed podocyte is foot process effacement. Effacement leads to a decrease in the filtration area [22] (see also Section 14). Although unproven at this point, it could be that this reduction in filtration area represents an energetically more favorable state. The combination of morphometric data with mathematical modeling suggests that foot process effacement leads to a reduction of the compressive forces required for the counteraction of filtration pressure, resulting in capillary dilatation and ultimately albuminuria [23]. Noteworthy, the podocyte is capable of quickly switching to a more epithelial appearance, including tight junctions and apical microvilli [8, 28]. This effect can be reversible, with podocytes regaining their normal morphology within minutes [29], suggesting that the morphological changes might, in part, occur at the post‐transcriptional/translational level. The following paragraphs will detail our current knowledge on podocyte structure and highlight some specific molecular specialties of this highly specialized epithelial cell.

### 2.2 Slit Diaphragm

**FIGURE 3:**
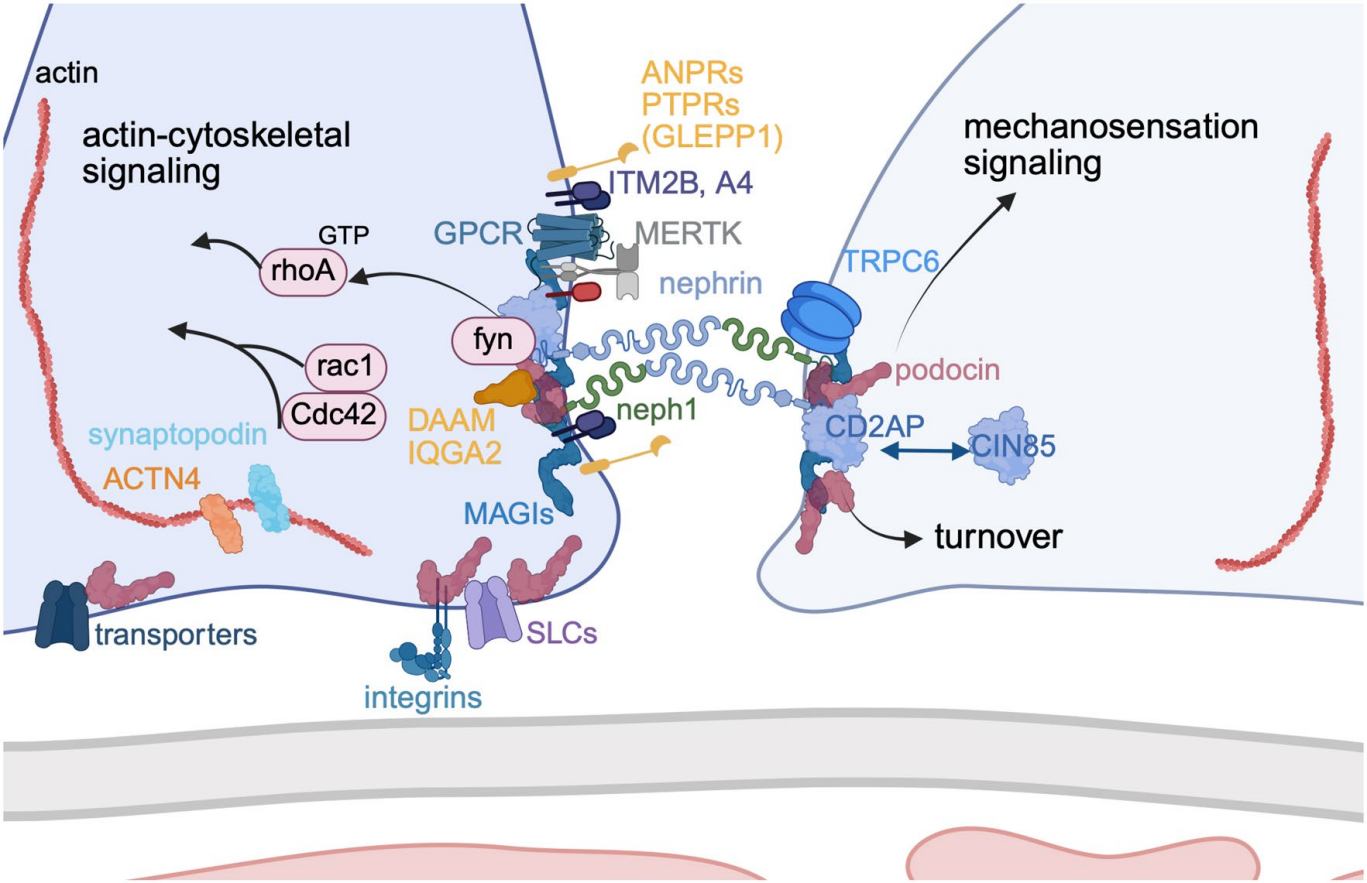
Molecular composition of the slit diaphragm. Scheme of hypothetical molecular composition of the rodent slit diaphragm, illustration restricted to structurally distinct constituents, modified from Kocylowski et al. [15]. 4A = A423; ANPRs = atrial natriuretic peptide receptors; CD2AP = CD2‐associated protein; CIN85 = Cbl‐interacting protein of 85 kDa; DAAM = disheveled‐associated activator of morphogenesis; GPCR = G‐protein coupled receptors; IQGA2 = IQ motif containing GTPase activating protein 2; ITM2B = Integral membrane protein 2B; MAGIs = membrane‐associated guanylate kinase with inverted orientation PDZ; MERTK = MER proto‐oncogene tyrosine kinase; PTPRs = protein tyrosine phosphatase receptors; SLCs = solute carriers; TRPC6 = Ca^2+^−permeable transient receptor potential channel 6; ACTN4 = alpha‐actinin‐4.

Foot processes are interconnected by slit diaphragms, which represent a lipid‐raft‐like structure in which essential podocyte proteins such as transmembrane proteins (like nephrin, neph1), integral membrane proteins (like podocin), structural proteins (like alpha‐actinin‐4 [ACTN4]), signaling adaptors (like CD2‐associated protein [CD2AP]), ion channels (like the Ca^2+^‐permeable transient receptor potential channel [TRPC]‐6), G‐protein coupled receptors (GPCR), and receptor tyrosine kinases (like MERTK [MER proto‐oncogene tyrosine kinase]) are organized into a multi‐protein complex (Figure 3). Lipid rafts, as small (10–200 nm diameter) specialized plasma membrane domains, are enriched with sphingolipids, cholesterol, and protein complexes that have roles in signal transduction. Cholesterol is enriched 5–8‐fold in lipid rafts compared with the rest of the plasma membrane and interacts with sphingolipids via its saturated hydrophobic side chains [30]. The importance of lipid rafts in the spatial organization of glomerular slit‐diaphragm proteins was recognized several years ago when nephrin and podocin were found to be enriched in these rafts [31]. Additionally, the cholesterol component of lipid rafts is required for the proper localization and function of slit‐diaphragm proteins [32]. As such, the binding of podocin enables cholesterol to associate with TRPC6, a step that is necessary for podocin‐dependent activation of this protein [33]. Besides podocin, many other proteins can bind cholesterol at the slit diaphragm; for example, caveolin‐1, a lipid raft‐associated protein that binds nephrin and CD2AP [34].

The slit diaphragm, as a highly sophisticated cell–cell contact, forms an adjustable, non‐clogging barrier through which glomerular filtration occurs. While the term ‘diaphragm’ implicates a thin‐layered sieve, studies now reveal that the slit diaphragm is composed of multiple layers of flexible transmembrane molecules to limit the passage of macromolecules [14]. The podocyte slit diaphragm is thought to display features of several types of cell–cell junctions, including tight, adhesion, neuronal, and gap junctions [14, 15] as proteins from tight (zonula occludens [ZO]‐1, junctional adhesion molecule [JAM]4, occludin, and cingulin) and adherens junctions (P‐cadherin, FAT atypical cadherin 1 [FAT1], and the catenin family of proteins) have been localized to the slit diaphragm over the years, in association with the key components of this cell–cell junction, namely nephrin and neph1 [35]. Both proteins represent immunoglobulin superfamily members and bind directly to the PDZ domain of partitioning defective protein (par)3 of the par apical polarity complex via their carboxy‐terminal type 1 (neph1) and type 2 (nephrin) PDZ‐binding motifs [36, 37]. Nephrin and neph1 form the core component of the slit diaphragm by a bipartite assembly with neph1 molecules spanning the lower part of the slit close to the GBM and nephrin molecules positioned in the apical side [14]. Recent cryo EM tomography of high pressure frozen murine glomeruli demonstrated that nephrin and neph1 are organized in a fish‐net pattern with a complex interaction pattern with multiple contact sites between both molecules [38] (see Figure 9). The stomatin protein family member podocin (NPHS2) generates a signaling hub in lipid rich membrane compartments for TRPC6 [33] and other slit diaphragm proteins as detailed above. This is thought to translate mechanical tension to ion channel action and cytoskeletal regulation [39]. On their intracellular C‐terminal parts, nephrin and neph1 are associated with several signaling adaptor molecules and scaffold proteins linking the slit diaphragm to the actin cytoskeleton [14, 40]. Combining our recent knowledge derived from improved microscopy techniques, such as cryo EM tomography [14], as well as from high‐resolution proteomic analysis of slit‐diaphragms affinity‐isolated from rodent kidney [15], it is thought that the slit‐covering layer of nephrin/neph1 molecules most likely does not operate as a filtration barrier [14, 41] but rather is endowed with context‐dependent dynamics via its co‐assembled protein network. As such, in the native slit diaphragm, nephrin, neph1, and podocin co‐assemble with distinct classes of proteins including components with enzymatic activity (MERTK), receptor‐triggered signaling (like atrial natriuretic peptide receptor [ANPRs], protein tyrosine phosphatase receptors [PTPRs]) and scaffolding function (integral membrane protein [ITM]2B and its interacting partner A423), rather than with cell–cell junctional proteins [15]. Further details on the podocyte specific proteins nephrin, neph1, and podocin are provided below (see Section 2.6).

### 2.3 Polarity

**FIGURE 4:**
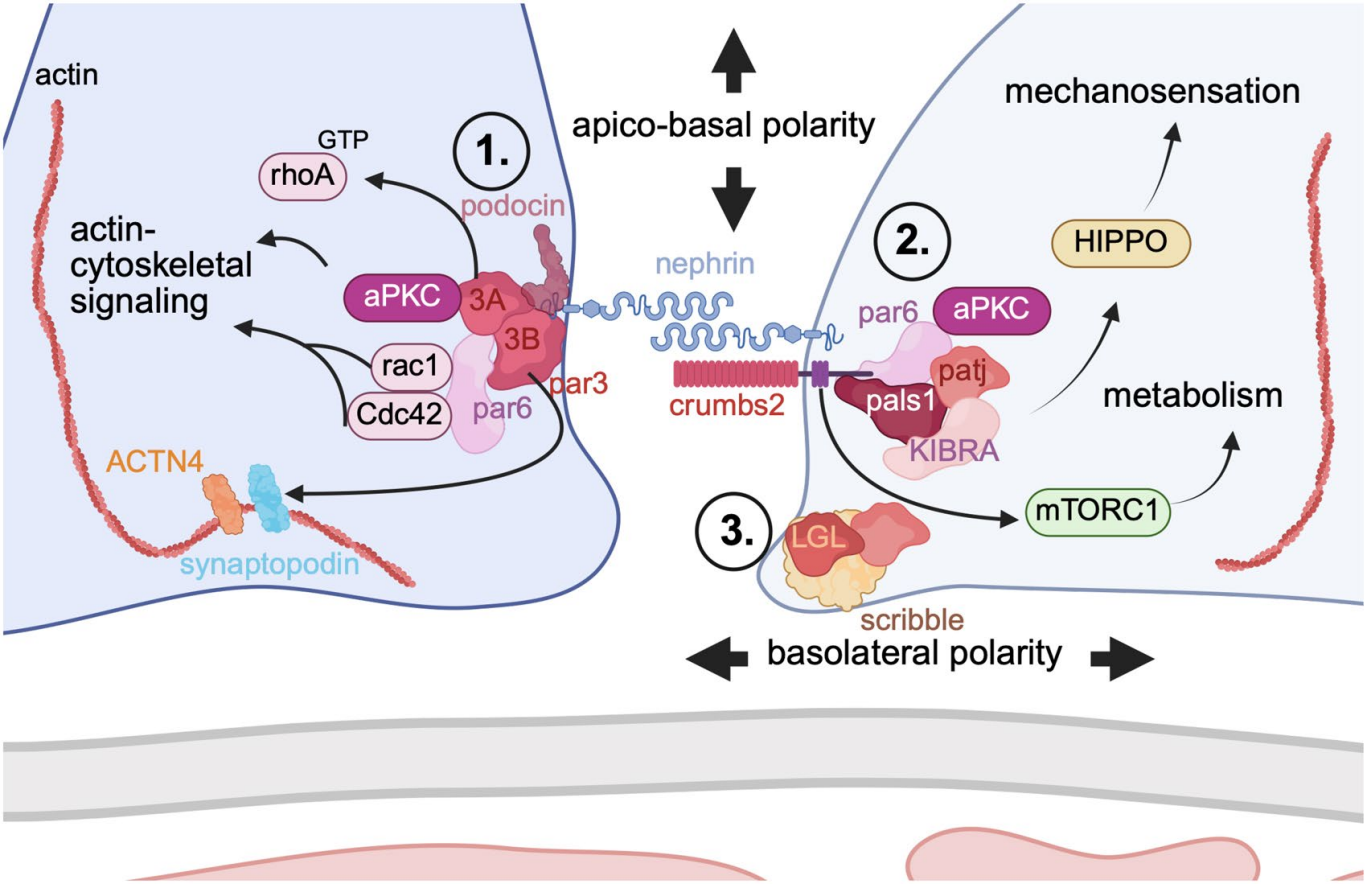
Polarity complexes of podocyte foot processes. The partitioning defective (PAR) complex (1.) and the crumbs (2.) complex facilitate apical polarity, whereas the scribble complex (3.) functions at the basolateral cell surface. The asymmetric distribution of polarity complexes is coupled to the asymmetric organization as well as the formation of the slit diaphragm. Central to the functions of the polarity proteins is their capability to create membrane‐associated nanodomains that recruit and control many downstream effectors, which in turn feed back to the polarity proteins to ensure a stable asymmetric cellular architecture. Basolateral polarity signaling via scribble is dispensable for podocyte function. Apical membrane expansions of podocytes are dominated by apical polarity complexes rather than by basolateral polarity signaling. ACTN4 = alpha‐actinin‐4; aPKC = atypical protein kinase C; Cdc42 = cell division cycle 42; DGL = discs large giant larvae; GTP = guanosine triphosphatase; KIBRA = kidney brain protein; LGL = lethal giant larvae; mTORC = mammalian target of rapamycin; Pals1 = protein associated with LIN7 1; Par = partitioning defective protein; Patj = InaD like protein; rac1 = ras‐related C3 botulinum toxin substrate 1; rhoA = ras homolog family member A.

Podocytes are polar cells. Polarity involves the asymmetric organization of most aspects of the cell, including the plasma membrane, intracellular organelles, and the cytoskeleton. Polarity of the mature podocyte is orchestrated by polarity proteins including the partitioning defective (PAR) complex, the crumbs, and the scribble complexes (Figure 4). The PAR and crumbs complexes facilitate apical polarity, whereas the scribble complex functions at the basolateral cell surface. The asymmetric distribution of polarity complexes is coupled to the asymmetric organization as well as the formation of junctional complexes, such as the slit diaphragm. Central to the functions of the polarity proteins is their capability to create membrane‐associated nanodomains that recruit and control a large number of downstream effectors, which in turn feed back to the polarity proteins to ensure a stable asymmetric cellular architecture. Among others, these complexes cooperate with the small guanosine triphosphatases (GTPases) Rac1 (ras‐related C3 botulinum toxin substrate 1), rhoA (ras homolog family member A), and CDC42 (cell division cycle 42) to modulate membrane trafficking, phosphatidyl inositol phosphate regulation, and the organization of the microtubular and actin cytoskeleton [42, 43, 44, 45]. The importance of podocyte polarity signaling is reflected by the high level of redundancy of the involved molecules [46, 47, 48].

The core of the PAR complex consists of par6 and atypical protein kinase C (aPKC), which localize together with the scaffold protein par3 to the podocyte slit diaphragm [36, 37]. Par6 (in its interaction with rac1 and CDC42 [49, 50]) is necessary for the activation of aPKC [51]. Par3 proteins share a high functional redundancy with different polarity proteins but also have specific functions. Par3A is the dominant par3 gene expressed in podocytes and found at the basis of the slit diaphragm, where it partially colocalizes with podocin. Studies in par3A knockout mice demonstrate that par3A is dispensable for slit diaphragm integrity due to a high redundancy with other polarity proteins such as par3B (a second *Pard3* variant known as *Pard3B/Pard3L* [52, 53] expressed in podocytes), lethal giant larvae (LGL), or protein associated with LIN7 1 (pals1), assuring the function of the GFB [46]. Par3B does not contain a classical aPKC binding domain and, consequently, does not directly interact with aPKC [52, 53]. Only the double knockout of par3A and par3B results in a podocyte phenotype [54]. Nonetheless, par3A acts in an aPKC‐par6 dependent way and regulates rhoA‐GTP levels, while par3B exploits par6 independent functions influencing synaptopodin localization. Hence, par3A and par3B link elements of polarity signaling and actin regulators to maintain podocyte architecture [54]. Atypical PKC signaling is essential for podocyte polarization and slit diaphragm positioning. As such, a podocyte‐specific deletion of the aPKC isoform lambda/iota causes severe morphologic podocyte alterations, including an apical displacement of the slit diaphragm and a mislocalization of apical proteins such as podocalyxin [37, 55]. In podocytes, aPKC seems not only to be important for podocyte maintenance and foot process assembly but also for the initial steps of process formation, as shown in a study using double podocyte knockout of aPKC isoforms lambda/iota and zeta [56].

Besides the PAR complex, the crumbs complex (comprises crumbs, pals1, inaD like protein [PatJ], and lin7 proteins [57] among others) also facilitates apical polarity in podocytes. Of the three crumbs (CRB) homolog proteins (CRB1, CRB2, and CRB3) expressed in mammalian epithelial cells, crumbs2 is highly abundant in podocytes. *CRB2* mutation was reported to be a cause of steroid‐resistant nephrotic syndrome [58, 59], potentially as disease‐associated CRB2 variants predominantly remain at the ER [60]. Deletion of crumbs2 in zebrafish [61] and mouse podocytes [62] causes severe podocyte defects, including disorganization of podocyte foot process architecture, loss of slit diaphragms, and apically mislocalized nephrin expression [61]. Crumbs2 interacts with FERM (band 4.1/ezrin/radixin/moesin) and PDZ (PSD‐95/Discs large/ZO‐1) motifs [63, 64] and in podocytes, an interaction of CRB2 with nephrin, mediated by their extracellular domains, was described [62]. Phosphorylation of the crumbs2 cytoplasmic domain disrupts its binding to moesin and allows it to instead form a complex with pals1 [65]. In developing podocytes, crumbs2 tyrosine phosphorylation is linked to podocyte metabolism via the mammalian target of rapamycin complex (mTORC)1 pathway, which plays a fundamental role in protein synthesis and cell growth [66]. Furthermore, crumbs controls HIPPO pathway proteins such as merlin/NF2 and KIBRA (kidney brain protein that binds to the multi‐PDZ domain adaptor protein patj, as part of the crumbs complex). KIBRA was not only found to be required for the directed migration of cultured podocytes [67] but also for the control of the HIPPO pathway in non‐podocyte epithelial cells, suggesting that crumbs may act as a direct or indirect mechano‐sensor of the HIPPO pathway [57]. In favor of such a mechanism, deficiency of the pals1 ortholog in *Drosophila* nephrocytes causes alterations in slit‐diaphragm‐like structures, and pals1 haploinsufficiency in mouse kidneys is associated with the upregulation of HIPPO pathway target genes as well as of genes of transforming growth factor (TGF)‐β signaling [68]. It will, therefore, be interesting to further elucidate how these proteins functionally interact with the PAR complex in the developing and mature podocyte.

Scribble (SCRIB) is a large cytoplasmic scaffold protein, which in polarized renal epithelial cells localizes to the adherens junctions and the lateral membrane in an E‐cadherin dependent manner [69]. The basolateral scribble complex includes discs large (DLG) and lethal giant larvae (LGL) proteins among others [57]. Similarly to the podocyte loss of par3A, podocyte loss of scribble does not result in a developmental or functional glomerular phenotype, and no susceptibility to glomerular stress [47]. This indicates that basolateral polarity signaling via scribble is dispensable for podocyte function. Hence, apical membrane expansions of podocytes are dominated by apical polarity complexes rather than by basolateral polarity signaling [47].

### 2.4 Adhesion Complex

**FIGURE 5:**
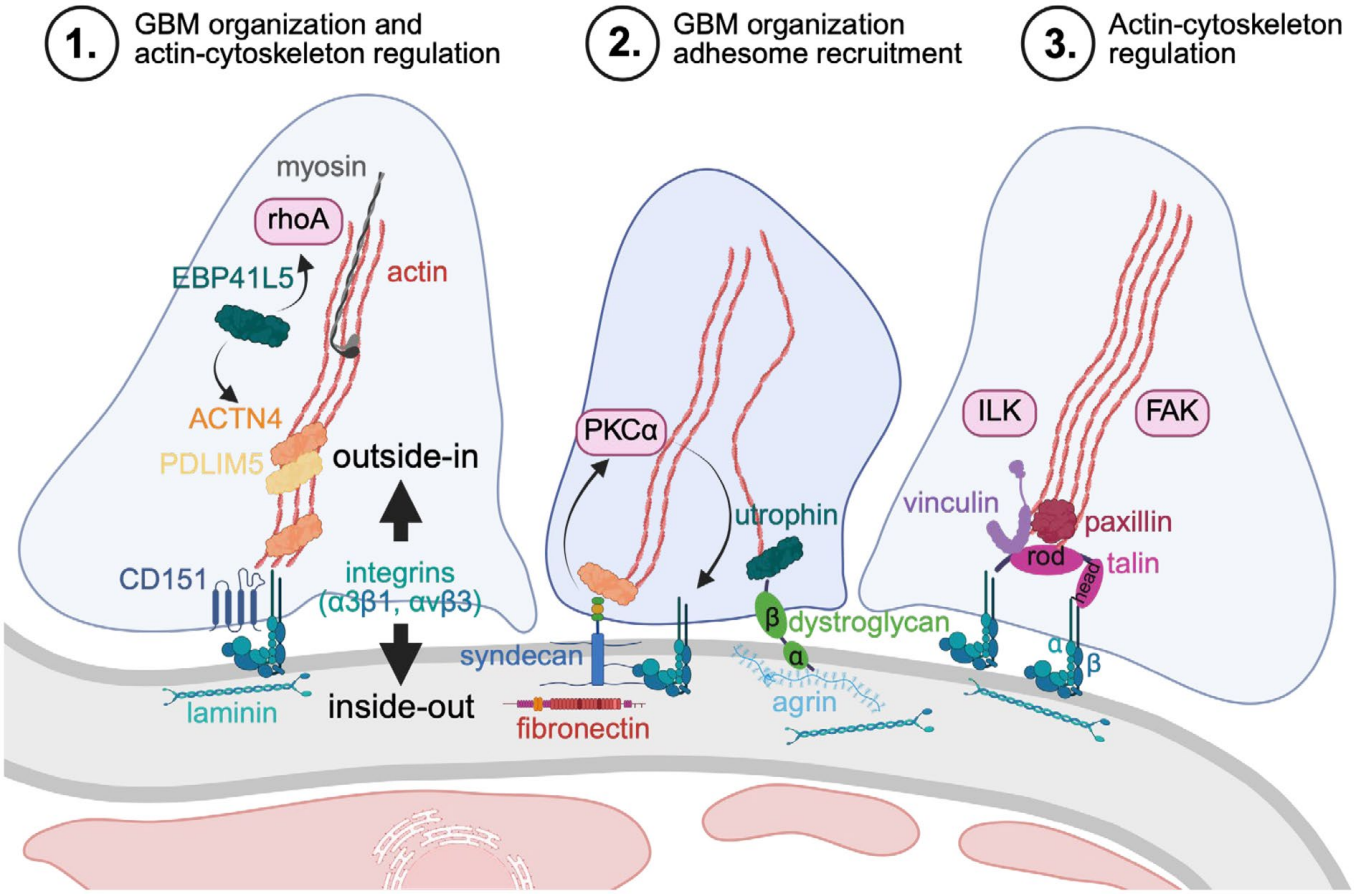
Foot process adhesion complex. On the molecular level, podocyte foot processes adhere to the underlying GBM by means of adhesion receptors, including integrins, syndecans, vinculin, talin, and dystroglycan. These molecules affect the organization of the GBM (inside‐out signaling) and/or the actincytoskeleton (outside‐in signaling) to different extents. ACTN4 = α‐actinin‐4; EBP41L5 = erythrocyte membrane protein band 4.1 like 5; FAK = focal adhesion kinase; ILK = integrin linked kinase; PDLIM5 = PDZ and LIM domain protein 5, PKC = protein kinase C.

Due to the constant exposure of podocytes to filtration forces, a tight adherence to the GBM is required to prevent their detachment, a major contributing factor to the progression of glomerular and chronic kidney disease [70]. On the molecular level, foot processes attach podocytes to the underlying extracellular matrix (ECM) of the GBM by specific proteins such as adhesion receptors, including integrins, syndecans, vinculin, talin, and dystroglycan [13]. These proteins exert their effects by mediating the regulation of the foot process actin‐cytoskeleton–like talin and vinculin, or by influencing the GBM organization like syndecans or dystroglycans. Integrins, as heterodimeric transmembrane receptors that connect the ECM with the actin cytoskeleton, are essential for the podocyte‐GBM interaction and influence both the actin cytoskeleton signaling and the GBM organization (Figure 5).

As in all cells, integrins are linked to an intracellular multiprotein complex (integrin adhesome) in podocytes that constitutes various adaptor proteins, GTPases, kinases, and phosphatases [71]. Based on the molecular repertoire, integrin adhesion complexes enable bidirectional signaling (also called inside‐out and outside‐in signaling), thus influencing intracellular processes as well as the interaction with the extracellular milieu. To date, 24 distinct integrins are differentiated, which consist of 1 of 18 alpha subunits and 1 of 8 beta subunits. Integrins are classified depending on their ligand preferences. The laminin‐binding integrin alpha3beta1 is the principal integrin at podocyte foot processes. As such, mutations in the *ITGA3* gene cause glomerular and skin disease in affected patients [72]. Like human mutations, *Itga3* deletion in mice results in severe proteinuria and a disorganized GBM [72]. The latter is thought to be due to the interaction of integrin alpha3beta1 with CD151, a surface molecule belonging to the tetraspanin family. The association of CD151 with integrin alpha3beta1 strengthens the foot process adhesion to laminin [73] and deletion of CD151 was shown to lead to glomerular dysfunction with a high degree of GBM disorganization [74]. In podocytes, integrin alpha3beta1 also establishes a tight linkage to the actin‐cytoskeleton (e.g., via talin‐1, kindlin‐2 [FERMT2] and alpha‐actinin‐4 [ACTN4]). Other adhesome components, such as integrin beta1 [75], integrin‐linked kinase (ILK) [76], and the FERM‐domain protein EPB41L5 [77] were also shown to be critical for podocyte maintenance. The list of critical adhesome proteins will most likely grow in the future, as a recent study suggests that the podocyte adhesome contains up to 182 different proteins [77]. Of the recently identified proteins, EPB41L5 represents a highly selective podocyte adhesome component, which influences podocyte spreading and adhesion via recruitment of ARHGEF18 (rho/rac guanine nucleotide exchange factor 18) and activation of rhoA‐rock1/2‐actomyosin signaling under dynamic conditions [77]. EBP41L5 also controls a localized adaptive module of the podocyte adhesome by recruiting PDZ and LIM domain protein 5 (PDLIM5) and alpha‐actinin‐4 (ACTN4) to integrin adhesion complexes, ultimately to regulate ECM assembly. As such, podocyte‐specific EBP41L5 deletion results in a shift in ECM composition characterized by diminished deposition of core GBM components, such as laminin alpha5 [78].

Integrins are linked to the foot process actin fibers at the adhesome by talin and vinculin. Talin is fundamental for the assembly of focal adhesions [79] and provides an intimate link between integrins at the GBM and the actin cytoskeleton. Talin comprises a talin rod and an atypical N‐terminal FERM domain (talin head). The talin head interacts with the integrin beta tails for integrin activation [80], as well as with phosphatidylinositol phosphate kinase gamma (PIPK) [81] and phosphoinositides [82]. The talin rod comprises a series of binding sites for integrins [83], actin [84] and cytoskeletal proteins such as vinculin [85]. In podocytes, talin is essential for GFB maintenance, and loss of talin‐1 leads to more severe podocyte alterations than the loss of integrin beta1 in mice [75]. Precisely how talin‐1 contributes to the formation and maintenance of podocyte foot processes remains to be established, as loss of talin‐1 in podocytes only causes a modest reduction in integrin beta1 activation, while causing a profound alteration of the actin cytoskeleton [86], possibly independent of integrin‐dependent functions or due to its interaction with integrin alphaVbeta3 rather than alpha3beta1 [87] at podocyte foot processes. Cleavage of talin‐1 between the head and rod regions by the protease calpain is essential for focal adhesion turnover and cell migration [88], and contributes to foot process effacement and can be counteracted by the endogenous calpain inhibitor calpastatin [86, 89]. Loss of other focal adhesion proteins in podocytes, such as focal adhesion kinase (FAK) or adapter molecule crk does not lead to a murine phenotype with proteinuria, even though in culture podocyte cell spreading is compromised [90, 91], underlining the intricacies of the podocyte adhesome.

Dystroglycan and syndecan act as key linkers between the podocyte cytoskeleton and ECM of the GBM. Of the surface (transmembrane) proteoglycans (syndecans), especially syndecan‐4 plays a crucial role in podocyte adhesion by interacting with ECM components (e.g., growth factors and fibronectin), an interaction that is vital for podocyte attachment, spreading, and migration [92]. Syndecan‐4 localizes to focal adhesions in podocytes [93] and heparan sulfate N‐sulfation of syndecan‐4 side chains plays an important role for podocyte–matrix interactions. As such, podocyte loss of bifunctional heparan sulfate N‐deacetylase/N‐sulfotransferase 1 (NDST1), a bifunctional enzyme that is ultimately responsible for N‐sulfation of heparan glycosaminoglycans produced by cells, results in a decreased clustering of syndecan‐4 and a decreased recruitment of PKCα, alpha‐actinin‐4, vinculin, and phosphorylated FAK to focal adhesions in mice [94]. Syndecan‐4 enhances podocyte matrix interactions through PKCα signaling, which results in the recruitment of adhesion proteins [93, 94]. For the recruitment of the adhesome, syndecan‐4 oligomerizes [95] and interacts with alpha‐actinin‐4 in an integrin beta‐independent manner [96].

Dystroglycan is synthesized as a complex of alpha‐ and beta‐subunits, with the beta‐subunit representing the intracellular and transmembrane portion of dystroglycan and the alpha‐subunit residing extracellularly on the basolateral and apical sides of podocytes [97, 98], suggesting a role in supporting the structural integrity of the slit diaphragm and overall podocyte architecture. Alpha‐dystroglycan regulates the positioning of GBM matrix proteins like laminin and agrin [99, 100], a function that requires a proper glycosylation of dystroglycan [98]. Beta‐dystroglycan interacts with utrophin and dystrophin [101] to connect to the podocyte foot process actin cytoskeleton.

### 2.5 Podocyte Cytoskeleton

**FIGURE 6:**
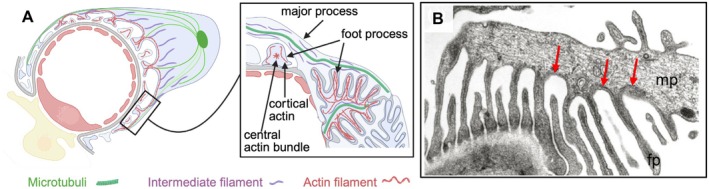
Podocyte cytoskeleton. Podocytes are pericyte‐like cells with a complex cytoarchitecture. (A) Scheme demonstrating the subcellular distribution of microtubuli, intermediate and actin filaments in podocytes. Under physiologic conditions at least two different actin networks are discernible in foot processes, a central Actin bundle within each foot process and the cortical actin network beneath the plasma membrane (also connected to adhesion receptors). (B) Electron microscopic micrograph depicting the origin of several foot processes (fp) from a major process (mp). The cytoskeleton of major processes predominantly consists of microtubules and intermediate filaments. Foot processes contain bundles of parallel actin filaments (red arrows) that connect neighboring foot processes in a clamp‐like fashion. Modified from C. Faul, et al. [103] TRENDS in Cell Biology 17, no. 9 (2007).

The podocyte cytoskeleton is highly elaborate in its architecture and regulating principles. Ultrastructurally, the podocyte cell body and major and foot processes contain vimentin‐rich intermediate filaments that assist in maintaining cell shape and rigidity. Large microtubules form organized structures along major processes, which are otherwise free of actin [102]. Actin is localized as a cortical net directly under the plasma membrane (for the maintenance of foot process morphology) and as long actin bundles in podocyte foot processes (for the generation of tensional forces), thus joining two neighboring foot processes in a clasp‐like fashion (Figure 6) [104, 105, 106]. While over the last decades substantial research has contributed to a detailed understanding of the specifics of the podocyte actin‐cytoskeletal regulation, our knowledge about the physiological function of the microtubular and intermediate filament network in podocytes is scarce (reviewed Ref. [106]).

Microtubules are highly dynamic, oriented structures composed of alpha/beta tubulin subunits, characterized by a fast‐growing (plus) end and a slow‐growing (minus) pole [107]. While genetic syndromes targeting the microtubular network frequently result in neurodevelopmental disorders, highlighting the strong dependency of neurons on a functioning microtubular network, a podocyte involvement is mostly absent [106]. To this end, the microtubular network is not essential for podocyte homeostasis and does not seem to be required for foot process formation as seen in a mouse model mimicking human TUBB2B mutations [108]. Different microtubule‐associated proteins (MAPs) are expressed by podocytes (e.g., MAP3, 4 and MAP1B) [109, 110, 111]; however, the constitutive knockout does not result in a podocyte phenotype [111]. Microtubular motor proteins, kinesin‐like protein KIF23 (CHO/MKLP1) and protein phosphatase 2A (PP2A) on the other hand, are (in culture) involved in podocyte process formation and orientation [112, 113]. Whether this also occurs in the in vivo setting is unknown.

Intermediate filaments are classified into six classes due to structural homologies. Podocytes homeostatically express the mesenchymal (class III) type intermediate filament vimentin [114], mostly within primary processes. Our knowledge of the role of class 3 intermediate filaments such as vimentin and desmin (the latter found in injured podocytes [115]) is limited, as their knockout in mice does not result in a podocyte phenotype [116, 117]. Podocytes also express nestin as a class VI intermediate filament [118]. When challenged, nestin might provide an anti‐apoptotic function in podocytes through an interaction with cyclin‐dependent kinase 5 (CDK5) [119]. Based on our current knowledge, intermediate filaments do not appear to convey a cytoskeletal stabilizing function to podocytes but might rather be involved in signaling functions or intracellular sorting/transport processes [106].

**FIGURE 7:**
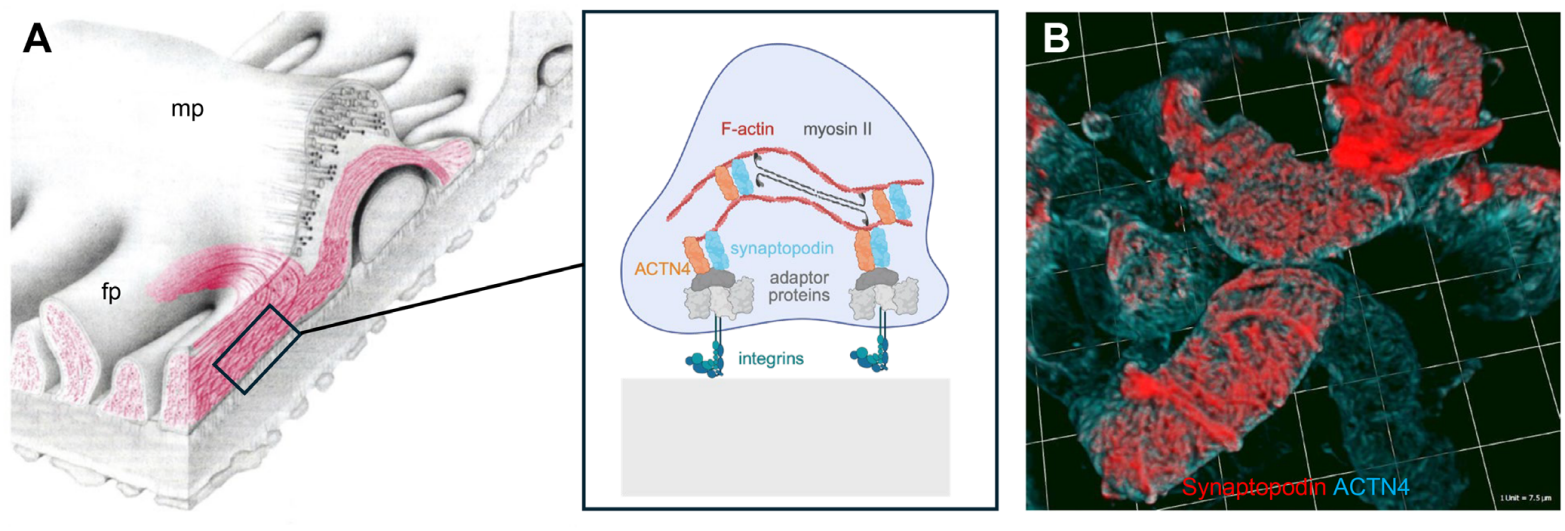
The podocyte foot process actin cytoskeleton. Contrasting podocyte major processes (mp), foot processes (fp) exhibit an elaborate actin cytoskeleton. (A) Scheme demonstrating the subcellular distribution and general molecular setup of the actomyosin filaments in foot processes, modified from C. Faul et al. [103] TRENDS in Cell Biology 17, no. 9 (2007). (B) 3D Confocal micrograph of a healthy capillary wall showing synaptopodin and alpha‐actinin‐4 (ACTN4) colocalization in major processes and interdigitating foot processes of healthy podocytes, courtesy Hani Suleiman.

In contrast to microtubules and intermediate filaments, the actin cytoskeleton constantly undergoes polymerization and severing within foot processes. Actin filaments are composed of globular actin monomers, which polymerize via ATP usage to filamentous (F‐)actin [120]. This process is heavily orchestrated by polymerizing and severing proteins (reviewed in [106, 120]). The actin‐related protein (Arp)2/3 complex was identified in its function as nucleators of actin polymerization, thereby determining the integrity of foot processes [121]. Further, Arp2/3 is responsible for the introduction of new branches on already existing actin filaments, a process required for the formation of lamellipodia [106]. Actin, alpha‐actinin‐4, and myosin form a contractile system in foot processes, which is regulated by the interplay of the actin binding proteins synaptopodin [122] and alpha‐actinin‐4 with rho GTPases. Alpha‐actinin‐4, as a non‐sarcomeric actinin, is needed to assemble large protein complexes [123]. As such, mutations in *ACTN4* result in decreased protein stability, subcellular mislocalizations, and increased actin binding capacity [106]. The well‐orchestrated actin and microtubule cytoskeleton of podocytes ensures a high plasticity of its process network [124, 125]. Healthy podocytes express different myosins, such as myosin IIA and IIB, myosin 1e [126], and myosin VI and IX, which are crucial for maintaining the integrity of the filtration barrier through cytoskeletal rearrangement and through regulation of the endocytic turnover of membrane components (Figure 7). Recently, sarcomere‐like structures with periodically spaced myosin IIA and synaptopodin were described in injured podocytes in vivo and in vitro [127, 128] (see Figure 14B), which are considered to represent mechanobiological mediators of podocyte recovery.

Extracellular cues are signaled to the actin cytoskeleton via focal adhesions and the slit diaphragm. At focal adhesions, the interplay of myosin and integrins is of special importance [129], especially to withstand shear stress forces at the filtration barrier [130]. Integrins are activated and linked to the actin cytoskeleton by the cytoplasmic protein talin‐1, a major component of focal adhesions [131] (see Section 2.4). Further, the slit diaphragm regulates podocyte dynamics by connecting signaling receptors like nephrin and podocin with the actin cytoskeleton [130]. Tyrosine phosphorylation of nephrin mediates lamellipodia formation and actin polymer extension in vitro and is further important for the maintenance of the slit diaphragm in vivo [132, 133]. Nephrin and podocin can both bind to the adaptor protein CD2‐associated protein (CD2AP), which then directly interacts with actin and synaptopodin [134, 135, 136]. Nephrin phosphorylation transmits the podocytes' reaction to mechanical stress and cytoskeletal dynamics via the cytoplasmic adaptor proteins nck1 and nck2 [133]. As nicely reviewed [137], the signaling pathways induced by nephrin tyrosine phosphorylation can be loosely defined based on their dependence on two different sets of residues. Group A tyrosines include Y1114 and Y1138/9, while group B tyrosines encompass Y1176, Y1193, and Y1217 (human nephrin numbering system). These tyrosines often coordinate signal propagation independently of each other, but at times, can also work in concert. Both group A and B tyrosines can be phosphorylated by src family kinases, including src, fyn, lyn, and yes [138], although each residue may not be phosphorylated to the same extent [137]. Phosphorylation of group A tyrosines induces binding of phosphatidylinositol 3‐kinase (p85/PI3K), ultimately leading to activation of protein kinase B (Akt) and rac1 and the recruitment of cofilin [132, 139]. Group A tyrosine phosphorylation seems to be especially involved in the lamellipodia formation seen in pathogenic foot process effacement, whereas group B tyrosine phosphorylation entails the formation of actin tails (growth of actin polymers at foot processes) believed to laterally stabilize foot process ultrastructure [137]. Phosphorylation of group B tyrosine sites results in the recruitment of nck, 1‐phosphatidylinositol 4,5‐bisphosphate phosphodiesterase gamma‐1 (PLC‐γ1), and SHC‐transforming protein 1 (ShcA). Nck subsequently recruits both serine/threonine‐protein kinase Pak [140, 141] and actin nucleation‐promoting factor N‐WASp [142], allowing for actin polymerization at nephrin.

**FIGURE 8:**
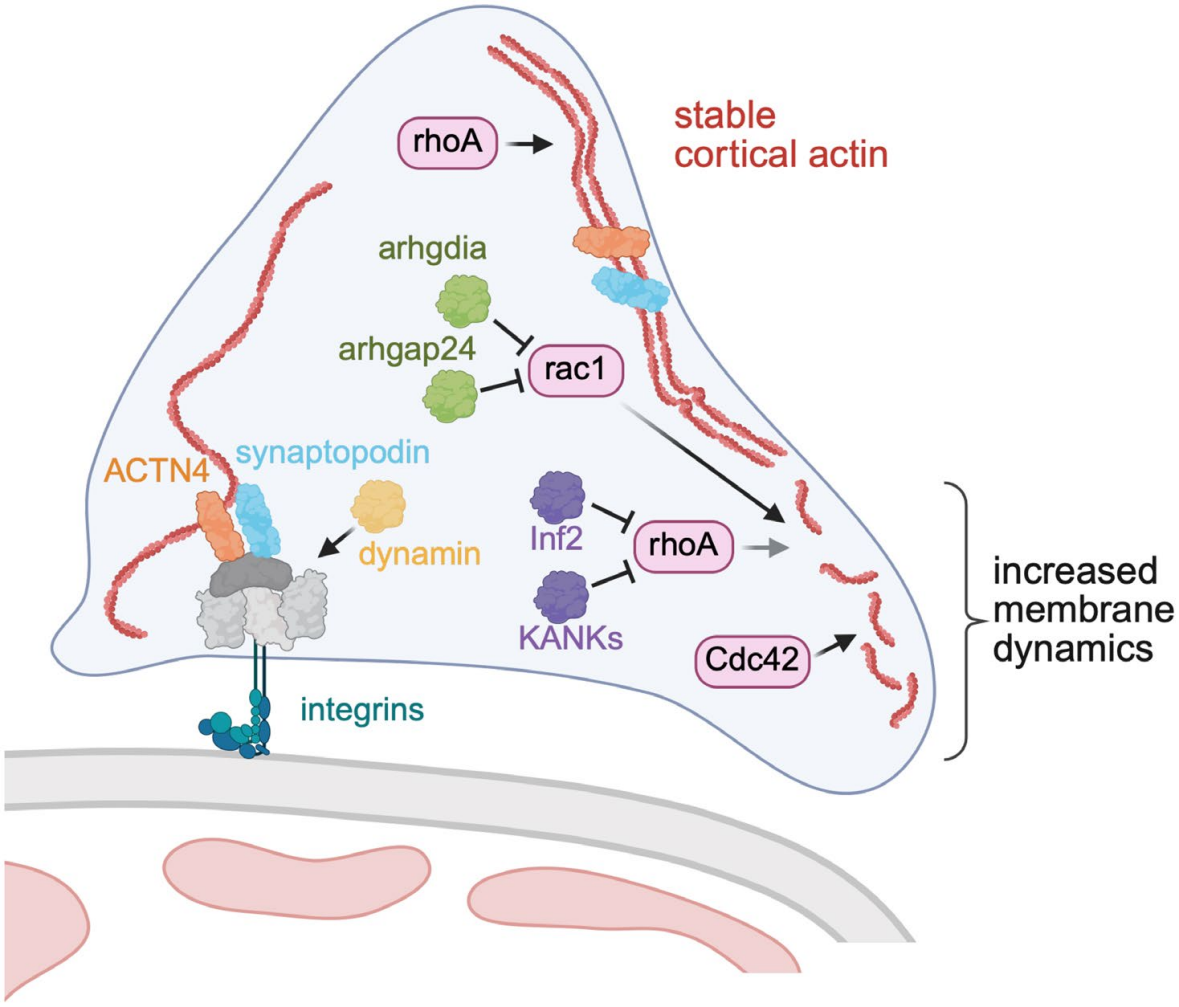
Podocyte foot process integrity depends on balanced actin‐cytoskeletal dynamics. The GTPases rhoA, rac1, and Cdc42 and their regulators the rho GDP‐dissociation inhibitor 1 (arhgdia), rho GTPase‐activating protein 24 (arhgap24), inverted formin (Inf)2 and KN motif and ankyrin repeat domain‐containing proteins (KANKs) define foot process cytoskeletal stability, ACTN4 = alpha‐actinin‐4. Modified from Schell et al. [106].

In general, actin dynamics and organization of the podocyte cytoskeleton are regulated by the ras homology (rho) family GTPases, with the three prototypic members rhoA, ras‐related C3 botulinum toxin substrate (rac1) and cell division control protein 42 (Cdc42) [143] and their regulators the rho GDP‐dissociation inhibitor 1 (Arhgdia), rho GTPase‐activating protein 24 (Arhgap24), inverted formin (Inf)2 and KN motif and ankyrin repeat domain‐containing proteins (KANKs). In culture, podocytes migrate by the formation of lamellipodia and filopodia, processes promoted by rac1 and Cdc42, respectively. In contrast, rhoA promotes formation of actin and myosin‐containing stress fibers in the cell body and backside of the cell [130]. By specifically deleting each GTPase, it was demonstrated that only Cdc42 loss resulted in a profound podocyte phenotype, characterized by massive proteinuria and dramatic ultrastructural abnormalities. On the contrary, loss of rhoA and rac1 does not result in an overt phenotype, at least under physiologic conditions [144, 145]. It is now widely accepted that the balance of rhoA to rac1/Cdc42 activity is central for the podocyte actin cytoskeleton: increased rhoA activity results in a stationary phenotype and intact foot processes, while rac1/Cdc42 activation promotes podocyte motility and effacement (Figure 8). This line of thought is substantiated in nephrotic patients, where cytoskeleton‐associated mutations (e.g., in *ACTN4* [146], *INF2* [147], or *ARHGDIA* [148]) relate to steroid‐resistant nephrotic syndrome or focal segmental glomerulosclerosis phenotypes (see [106] for details). ARHGDIA belongs to the rho GDI (GDP dissociation inhibitor) family and ensures that rho family members are kept in their inactive GDP‐bound form within the cytosol. In *Arhgdia* null mice, inhibition of rac1 overactivation suppressed albuminuria [149], emphasizing the importance of balanced cytoskeletal dynamics for podocyte integrity. This process can be regulated by different factors, for example, by calcium influx through transient receptor potential cation channel subfamily C member 6 (TRPC6) (summarized in [130]). TRPC6 forms complexes with rhoA at the slit diaphragm, and its activity is modulated by nephrin and podocin [150, 151]. TRPC6 plays several roles in activation of rhoA and rac1 in response to mechanical stress, thereby influencing the function of synaptopodin, nephrin, and podocin via regulation of intracellular calcium levels (reviewed in [152, 153]). Dynamin, a large and multi‐domain GTPase important for focal adhesion function, colocalizes with actin, thereby contributing to filopodia formation and *de novo* actin polymerization [130, 153].

Podocytes physiologically react to changes in glomerular capillary pressure (mechanical stress) and increased glomerular filtration rate by changing their morphology and filter function through actin cytoskeletal rearrangements [154, 155]. However, up to date, little is known about how those changes influence the functional connection with the GBM and the impact on the integrity of the glomerular filtration barrier in reaction to mechanical stress. In response to shear stress by filtrate flow across the filtration barrier, podocytes in vitro reorganize their actin cytoskeleton in a way that stress fibers are lost and vinculin expression at focal adhesions is lost. Vinculin is an adaptor protein present in focal adhesions linking integrins with the actin cytoskeleton, and loss leads to alterations in focal adhesion size as well as in increased cell migration [156]. Another family of actin‐binding proteins that link integrins to the actin filaments are the filamins. Filamins are necessary for branching of the actin cytoskeleton and can bind many signaling molecules by their 24 Ig‐like domains, thereby playing an important role in mechano‐sensing and signaling [157, 158]. Filamin A, as the main family member in cultured podocytes, is upregulated upon mechanical stress, and knockdown of filamin A results in alteration in focal adhesion and cytoskeleton morphology leading to increased motility in culture [159]. This very likely relates to an enhanced flexibility of podocyte foot processes to react to pressure alterations within the glomerular capillaries. Very recently, the zinc‐binding phosphoprotein zyxin was discovered to represent another major player in the mechanical stress response of podocytes: upon mechanical stress, zyxin is upregulated and relocalizes from focal adhesions to actin filaments and to the actin‐rich center (ARC), a structure observed in stretched podocytes, thereby supporting the maintenance of the blood filtration upon mechanical stress [160].

In summary, actin cytoskeletal dynamics in podocytes can be regulated on many different levels, which emphasizes its important function in podocyte health. For more in‐depth details on the complexity of the podocyte actin cytoskeletal regulation, please refer to the mentioned reviews.

### 2.6 Molecular Specialities

**FIGURE 9:**
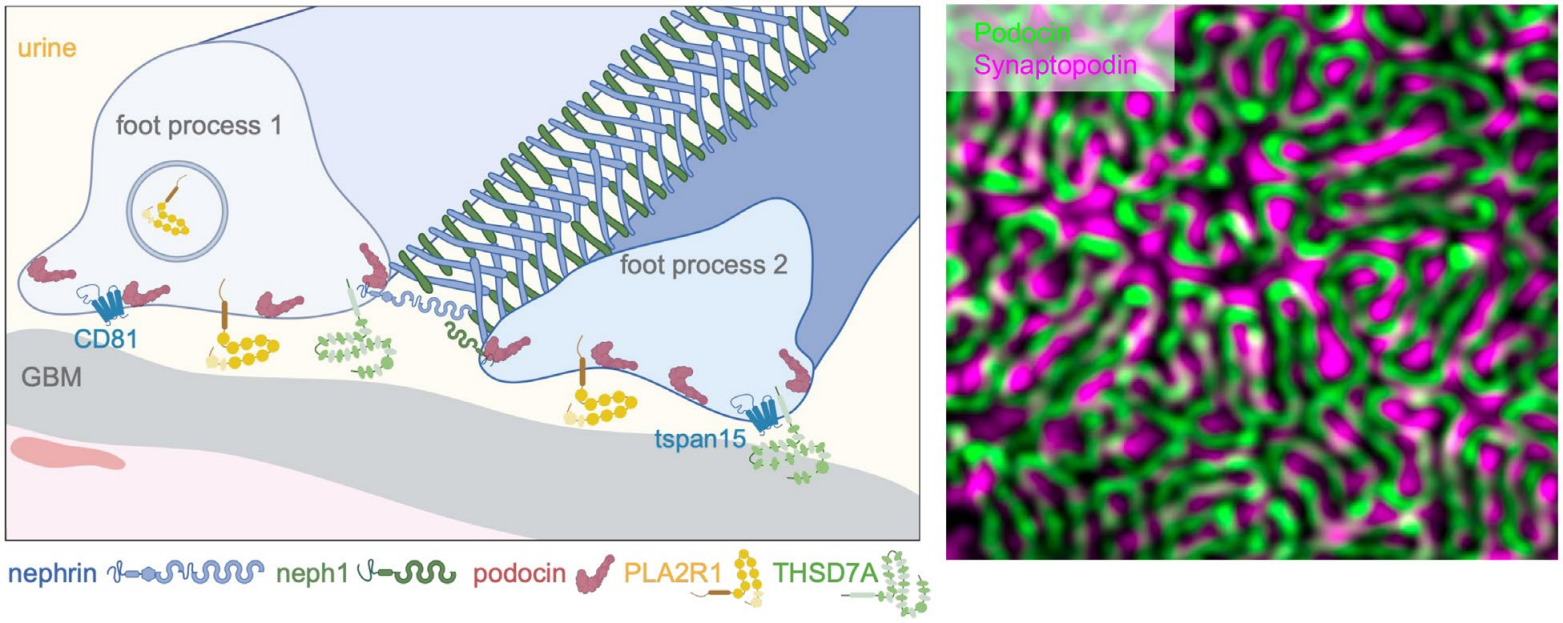
Podocytes express unique proteins. Scheme depicts the localization of the proteins highlighted in this chapter at foot processes. Micrograph of podocin and synaptopodin expression in foot processes covering the glomerular capillary: courtesy: Nicole Endlich. GBM = glomerular basement membrane; PLA2R1 = phospholipase A2 receptor 1; THSD7A = thrombospondin type‐1 domain‐containing protein 7A; Tspan = tetraspanin.

Within the kidney, podocytes selectively express an essential set of proteins, such as the central slit diaphragm proteins nephrin, neph1, and podocin, as well as the podocyte foot process proteins PLA2R1 and THSD7A (Figure 9) that in the recent two decades have been identified due to their target function in membranous nephropathy, an autoimmune podocyte injury. Due to their importance for podocyte function and disease development, these selected proteins will be highlighted in this paragraph.

#### 2.6.1 Nephrin

The podocyte foot process transmembrane protein nephrin [161] is essential for developing and maintaining slit diaphragms, as functional loss in humans results in heavy proteinuria [162]. Nephrin belongs to the immunoglobulin superfamily of cell adhesion molecules and spans the filtration slit with its large extracellular domain in interaction with neph1, thus forming the last barrier to protein of the kidney filter. Nephrin's extracellular domain contains one fibronectin‐like III (FNIII) motif and eight immunoglobulin (IgG)‐like regions, which allow for homophilic interactions of nephrin molecules in trans as well as heterophilic interactions with neph1. Nephrin localization to the slit diaphragm is dependent on its cytosolic interaction with podocin [163] at its R1160 residue, which directs nephrin localization to lipid rafts at the cell surface [136]. On the intracellular side, nephrin and neph1 interact with a multitude of intracellular signaling proteins within a macro‐molecular protein assembly [15] to finally regulate podocyte actin‐cytoskeletal dynamics, adhesion, barrier turnover, and survival. As a recent study showed, the interactome of murine nephrin and neph1 includes about 29 bona fide proteins, which comprise type 1 transmembrane proteins and soluble proteins attached to membrane proteins via lipid anchors or via WW or PDZ domain(s) [15]. Some interactors exhibit extended extracellular domains comprising Ig‐ or Ig‐like domains implicated in transcellular protein–protein interactions [15]. Nephrin is heavily phosphorylated in its steady state [15]. At least 6–10 tyrosine residues are phosphorylated, thus establishing a signaling platform [137] to regulate nephrin endocytosis, the podocytes reaction to mechanical stress and cytoskeletal dynamics [133] (also see Section 2.5). Levels of nephrin phosphorylation depend on src homology 2 domain‐containing protein tyrosine phosphatase 2 (SHP‐2) [164]. Nephrin is believed to be dynamically endocytosed, a process thought to clean the slit diaphragm from serum proteins. Nephrin endocytosis is mediated by its interaction with beta‐arrestin in a clathrin [165] and dynamin‐dependent [166] fashion. Besides beta‐arrestin, nephrin endocytosis is triggered by protein kinase C (PKC)α and CIN85/Ruk_L_ (Cbl‐interacting protein of 85 kDa), the paralog of CD2AP [167, 168]. Additionally, nephrin turnover and plasma membrane levels are regulated by SUMOylation, a post‐translational modification with SUMO (small ubiquitin‐like modifier) [169]. Recently, a connection between nephrin clustering and endocytosis was described, which appears to be regulated by threshold levels of nephrin tyrosine phosphorylation and nck SH3 domain signaling with the actin nucleation‐promoting factor N‐WASp and dynamin [170]. While abundant nephrin endocytosis is described in Drosophila nephrocytes [171], the extent of nephrin endocytosis in mammals is unclear. In mice, nephrin half‐life is long, as an inducible podocyte knock‐out of nephrin in adults results in the development of proteinuria only 8 weeks after induction, whereas inducible podocyte knock‐out of neph1 or podocin in adult mice results in rapid onset of proteinuria after 2 weeks [15]. Recently, nephrin was identified as an autoimmune target in a large number of patients with minimal change disease [172, 173], with anti‐nephrin antibodies sufficient to cause proteinuria in a rabbit [173], positioning the podocyte further center stage of autoimmune targeting [174].

#### 2.6.2 Neph1

Kin of IRRE‐like protein 1 (Kirrel 1), also known as neph1 is a type I transmembrane protein of the immunoglobulin superfamily that, unlike nephrin, is not only expressed in podocytes but also in other renal cell types such as proximal tubular cells [175], suggesting that it could play other roles than nephrin. The extracellular domain of neph1 forms a cis‐ and trans‐interacting complex with nephrin. The coordinated localization of neph1 (and nephrin) at the podocyte slit diaphragm depends on the exocyst complex, a highly conserved multi‐protein trafficking complex that plays a crucial role in the targeting and tethering of secretory vesicles to specific plasma membrane sites [176]. Despite the common idea that neph1 works in tandem with nephrin, neph1 appears to be of particular importance for the structural stability of the slit diaphragm [15]. Mice lacking neph1 are proteinuric and reveal effacement of podocyte foot processes in the first postnatal days [175]. Neph1 is involved in the rearrangement of the actin cytoskeleton as a signaling mediator, emphasizing its essential role in the organization and functional assembly of the slit diaphragm. For this, neph1 is tyrosine phosphorylated at its intracellular domain. Primarily, the phosphorylation of its residues 637 and 638 is important for the recruitment of several proteins, including growth factor receptor–bound protein 2 (Grb2), tyrosine protein kinase Csk, and ZO‐1 [177, 178]. Recently, neph1 (and nephrin) was identified as a receptor protein for hepatocyte growth factor (HGF), suggesting receptor‐based functions of the protein as part of outside‐in signaling properties. HGF binding to neph1 resulted in its phosphorylation, a process involved in podocyte recovery from injury [164].

#### 2.6.3 Podocin

Podocin is a member of the stomatin family, which consists of hairpin‐like integral membrane proteins with intracellular NH(2)‐ and COOH‐termini. This 42 kDa small podocyte protein is a lipid‐raft associated component of the slit diaphragm [31], whose expression is first seen in the capillary loop stage of the developing podocyte. As podocin is present in high‐order oligomers in mature podocytes, it serves a scaffolding function essential for the structural organization of the slit diaphragm and the regulation of its filtration. The scaffolding function of podocin is likely to occur through its interaction with scaffolding proteins such as the tetraspanin CD81 [15]. Due to its interactions with nephrin and the cytoplasmic adaptor protein CD2AP [31], podocin is involved in signaling pathways ultimately affecting the podocyte actin cytoskeleton. Within the podocin‐lipid complex, the calcium channel TRPC6, which is a sensor of mechanically and osmotically induced membrane stretch, is clustered and regulated, thus potentially translating mechanical tension to ion channel action within the slit diaphragm [33]. Podocin is not exclusively localized to the slit diaphragm but rather displays a broader distribution over the plasma membrane of podocyte foot processes [15]. The recently established podocin interactome indicates that this protein may serve further diverse functions in podocytes. In addition to its established role as an anchor of the slit diaphragm, it may act as a cytoplasmic interactor/linker for a number of solute carriers of the SLC‐type (Na(+)/dicarboxylate cotransporter 3 [S13A3], sodium‐independent organic anion transporter S22AJ, sodium‐dependent neutral amino acid transporter B(0)AT1 [S6A19], sodium/myo‐inositol cotransporter 2 [SC5AB], or amino acid transporter heavy chain SLC3A1 [SLC31]) and other transporters (ATP‐binding cassette [ABC] transporter‐A9, lysosomal dipeptide transporter MFSD1) in the plasma membrane of podocytes [15]. The individual transporters identified differ in their substrate(s) ranging from organic anions/cations to amino acids and vitamins, transport systems not directly related to podocyte function yet. Podocin turnover in podocytes is regulated, like nephrin, by the adaptor protein CIN85/Ruk_L_. Podocin binds to the coiled‐coil domain of CIN85/Ruk_L_ in a functional competition with CD2AP [167]. This interaction is associated with the ubiquitination and endocytosis of podocin and nephrin [167]. Podocin endocytosis was also shown to involve an interaction with sorting nexin 9 upon podocyte injury [179]. Inducible deletion of podocin in adult mice results in the rapid development of proteinuria within less than 14 days, turning nephrotic at day 21, underscoring the central role of this protein for podocyte function. A 2.5‐kb fragment of the human podocin (*Nphs2*) promoter was the first promoter construct used for the generation of podocyte‐specific transgenic mouse models [180] and is widely used in podocyte research.

#### 2.6.4 PLA2R1

M‐type phospholipase A_2_ receptor 1 (PLA2R1 or PLA_2_R1) was discovered 2009 as a new podocyte foot process protein targeted by autoimmunity in membranous nephropathy [181], which in the kidney is only expressed by podocytes. Besides on podocytes, PLA2R1 is also present in the lungs and on leukocytes [182, 183]. This type‐1 transmembrane protein is composed of an N‐terminal cysteine‐rich domain, a fibronectin type II domain and eight C‐type lectin domains, a transmembrane domain and a short intracellular C‐terminal tail and represents one of four mammalian members of the mannose‐receptor family [184, 185]. Members of the mannose‐receptor family exist in both extended and bent conformations that confer distinct ligand binding and multimerization capacities [186]. A shorter, alternatively spliced PLA2R1 transcript encodes for a soluble form of the receptor, which in the kidney is less expressed at the mRNA level than the membrane‐bound PLA2R1 [187]. PLA2R1 undergoes clathrin‐coated pit mediated endocytic recycling, which is rapid and ligand‐independent [188]. Functionally, PLA2R1 serves as a binding protein for secreted phospholipase A_2_, a group of small 13–18 kDa phospholipid‐hydrolyzing enzymes, that are secreted by certain species in different types [189]. The normal endogenous ligands of PLA2R1 are probably the pancreatic‐type sPLA_2_ as well as inflammatory‐type sPLA_2_. Pancreatic‐type sPLA_2_ are implicated in digestive functions, fertilization, cell proliferation, and contraction of vascular and airway smooth muscles, whereas inflammatory type sPLAs are associated with propagation of inflammation and hyper sensibilization of the organism [190]. The biological responses induced by sPLA_2_s are based on their ability to bind to heparan sulfate‐containing proteoglycans of cell membranes [191, 192], to perturb plasma membrane symmetry by a scramblase‐dependent mechanism [193] and finally by their interaction with specific membrane receptors [185], such as PLA2R1. In non‐kidney cells, PLA2R1 signals through the tyrosine protein kinase Janus kinase 2 (Jak2), thus controlling cell growth and senescence, protecting from tumor initiation [194]. The physiological function of PLA2R1 in podocytes is still unclear, mostly related to the fact that rodent podocytes do not express the receptor, hampering investigations. Based on podocyte cell–culture experiments, PLA2R1 is thought to influence podocyte autophagy downstream of sPLA_2_‐IB binding [195], suggesting that ligand‐receptor functions could be involved in podocyte injury. Additionally, the interaction of the extracellular PLA2R1 domain with collagen through its fibronectin type II domain was suggested to be required for the internalization of collagen [196]. As detailed biochemical analyses were unable to substantiate an involvement of human PLA2R1 in binding or internalization of type 1 or type 4 collagen [197], further investigations are warranted in podocytes. Recently, surface cleavage of PLA2R1 was described by A disintegrin and metalloproteinase (ADAM) proteases 10/17 (see also Section 13.2) in human podocytes [198, 199], the biologic consequences of this cleavage event for podocyte biology is not clear.

#### 2.6.5 THSD7A

Thrombospondin type‐1 domain‐containing 7A (THSD7A) is a large transmembrane glycoprotein like PLA2R1, and again like PLA2R1 was recently identified as a novel podocyte foot process protein targeted by autoimmunity in membranous nephropathy [200]. The extracellular topology of THSD7A is best described as a tandem string of 21 thrombospondin type 1 domains [201]. THSD7A strictly localizes to podocyte foot processes of humans and rodents alike [202] and in podocyte development is first detected at the capillary loop stage, when the basal podocyte membrane interdigitates for the formation of foot processes [202]. Super‐resolution microscopy suggests that in mature podocytes THSD7A localizes basally to the slit diaphragm bridging proteins nephrin and neph1 and resides in direct neighborhood to integrin beta1. In cultured podocytes, THSD7A expression is associated with increased cell size, enhanced adhesion, and reduced detachment, and finally with a decreased migration ability [202], suggesting that THSD7A is involved in stabilizing membrane dynamics and thus maintaining the integrity of the GFB. Parts of these functions in podocytes might be related to an interaction of THSD7A with integrins, especially with integrin beta3 as demonstrated in human endothelial cell culture systems [203]. Recent findings demonstrate that THSD7A in conjunction with tetraspanin (tspan)15 is an important stabilizer of the surface protein sheddases ADAM10 at the podocyte surface and a strong inducer of filopodogenesis in the absence of ADAM10 [199], suggesting a role for uncleaved, surface‐expressed THSD7A in the organization of functional foot process domains and membrane dynamics.

### 2.7 Similarities to Neurons

**FIGURE 10:**
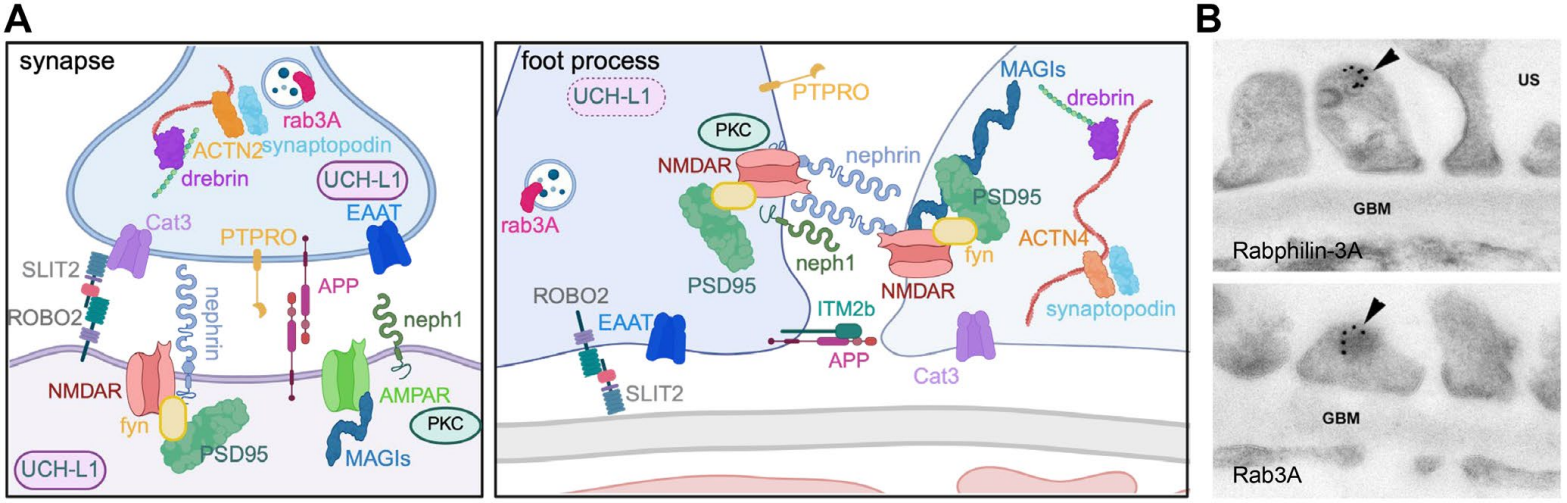
Molecular communalities between neurons and podocytes. (A) Scheme depicting the common molecular players expressed in neurons at synapses and podocytes at foot processes. (B) Immunogold EM to rabphilin‐3A and ras related protein rab3A in a normal rat kidney. Note the gold particles roundly distributed along vesicles contained in podocyte foot processes (arrowheads). US, urinary space; GBM, glomerular basement membrane. Original magnification ×36 000, 10‐nm gold particles, modified from Rastaldi et al. [204]. ACTN = alpha‐actinin; AMPAR = alpha‐amino‐3‐hydroxy‐5‐methyl‐4‐isoxazolepropionic acid receptor; APP = amyloid precursor protein; Cat3 = amino acid transporter SLC7A3; EEAT = amino acid transporter SLC1A3; ITM2B = integral membrane protein 2B, MAGIs = membrane‐associated guanylate kinase, WW and PDZ domain‐containing protein; NMDAR = *N*‐methyl‐D‐aspartate receptor; PKC = protein kinase C; PSD95 = disks large homolog 4; PTPRO = protein tyrosine phosphatase receptor type O (GLEPP1); ROBO2 = roundabout guidance receptor 2; SLIT2 = slit homolog 2 protein; UCH‐L1 = ubiquitin C‐terminal hydrolase L1.

Comparative analyses between podocytes and neurons help unravel related potential biological principles of podocytes. Both cell types are unique in our body; nonetheless, they share several similarities at the morphological and molecular level (Figure 10) substantiating the existence of a brain‐kidney axis [205]. Both cell types are postmitotic and develop long microtubule‐based protrusions coupled with short actin‐based foot processes or dendritic spines, respectively. Proteins expressed predominantly or even exclusively by both cell types regulate their function and morphology. The post‐synaptic density (PSD) of neurons and the cytoplasmic side of the glomerular slit diaphragm are regions of electron‐dense material [206, 207] and share common characteristics. In the PSD, neurotransmitter receptors and ion channels are expressed and linked to synaptic cell adhesion molecules (CAMs) and connected proteins. Podocytes and neurons show restricted expression of the same essential proteins, such as nephrin, neph1, neph2, synaptopodin, protein tyrosine phosphatase receptor type O (PTPRO/GLEPP1), the amino acid transporters Cat3 (SLC7A3), EAAT (SLC1A3), and the GTPase ras‐related protein rab3A, among others, reflecting similar characteristics and functions in those highly specialized cells [208]. Addressing all commonalities of podocytes and neurons is under the scope of this review; however, they are nicely summarized elsewhere [208]. A few commonalities will be highlighted in the following paragraphs.

In the developing brain, the podocyte slit diaphragm protein nephrin is expressed in several regions, like spinal cord, hippocampus, olfactory bulb, and cerebellum by radial glial cells, which give rise to neurons, astrocytes, and oligodendrocytes [209] with yet unknown function. It is believed that loss of nephrin during brain development is compensated by other proteins, as *Nphs1*‐knockout mice do not show any morphological brain alterations until postnatal day 1. However, complete loss of nephrin is lethal in the first 24 h after birth due to massive proteinuria precluding further brain analysis [210]. In the adult rodent brain, nephrin is expressed in the corpus callosum, the pons, and cerebellum (in granule as well as Purkinje cells) in proximity to synaptic proteins. There, it interacts with the tyrosine kinase fyn, N‐methyl‐D‐aspartate receptors (NMDAR), and the scaffolding protein discs large homolog 4 (DLG4/PSD95) [211], thus taking part in synapse maturation, glutamate exocytosis [212] and remodeling of the actin cytoskeleton [213]. The tyrosine protein kinase fyn regulates nephrin signaling by phosphorylating its intracellular domain. Mice lacking fyn function show podocyte foot process effacement and proteinuria, as well as impaired long‐term potentiation (LTP) and spatial learning [212]. At the neuronal postsynaptic site, fyn phosphorylates and thereby activates NMDAR, leading to action potential conduction and actin cytoskeleton remodeling, both processes induced by the neurotransmitter glutamate [214]. The secretory machinery of glutamate also contributes to the integrity of the glomerular filtration barrier. Applied NMDAR antagonists lead to cytoskeletal remodeling and rearrangement of nephrin in cultured podocytes, which is reversible by applying NMDAR agonist [215]. Furthermore, the dysfunction of rab3A, a small GTPase regulating glutamate exocytosis, results in altered synaptic vesicle release in neurons [216]. In podocytes, impaired rab3A functionality also results in enhanced exocytosis leading to increased proteinuria. Moreover, blocking NMDAR function by ketamine in general anesthesia applications leads to albuminuria in humans [204, 215]. These findings suggest an important role of glutamatergic signaling in podocytes. Neph1 and neph2 were also found to be expressed in neurons and podocytes. In the brain, both proteins are involved in neuronal synapse formation and cell–cell recognition. Studies in the nematode *C. elegans* reveal that the orthologues SYG‐1 and SYG‐2 are important for proper localization of synapses [217]. Mouse experiments confirm this finding and further reveal that neph1 and neph2 act via the calmodulin‐associated serine/threonine kinase (CASK) [218], a kinase also identified in podocytes and found to be associated with focal segmental glomerulosclerosis (FSGS) upon secretion [219].

Synaptopodin, a proline‐rich protein, is crucial for the identity of the actin‐based protrusions in both podocytes and neurons. To fulfill this function, synaptopodin is differentially modified at the post‐translational level with a 100 kDa isoform in neurons and a 110 kDa isoform in podocytes. Synaptopodin regulates dendritic spine stability in neurons by binding to alpha‐actinin‐2, and in podocytes, it stabilizes foot processes by binding to alpha‐actinin‐4 [135, 220], both actin‐binding proteins. Synaptopodin's function is regulated by protein kinase C (PKC), which is highly enriched at the postsynaptic density of neurons, as well as in foot processes [221]. Another protein involved in the formation and motility of neuronal or podocyte processes is the actin‐binding protein drebrin (developmentally regulated brain protein), which links the actin cytoskeleton to the microtubule network [222]. After neuronal differentiation, drebrin becomes important for neuronal migration and neurite extension [223]. Species differences can be observed in kidneys: while drebrin expression in rodent podocytes is restricted to early developmental stages, it is highly enriched in adult human podocytes [224] with yet unknown function.

The protein tyrosine phosphatase receptor type O (PTPRO/GLEPP1) is important in the development of dendritic spines and foot processes as well as for assembling the cytoskeleton, leading to abnormal cellular morphology when depleted in both cell types [225, 226, 227]. Forming and maintaining cellular protrusions, like foot processes and dendritic spines, requires the fresh amino acid (AA) transport to their cellular destination. Podocytes and neurons share common amino acid transporters (AATs) like Cat3 for cationic AA like arginine or EAATs for anionic AA like glutamate for that purpose [228]. Roundabout guidance receptor 2 (ROBO2) and its ligand slit homolog 2 protein (SLIT2) regulate axonal guidance and migration across the midline of the developing neural tube [229]. Both proteins are also responsible for proper renal development, showing high expression in early stages while being restricted to podocytes when the uretic bud and nephrons have formed [230]. Membrane‐associated guanylate kinase, WW and PDZ domain‐containing protein 2 (MAGI‐2), a scaffolding protein, is highly expressed in neuronal synapses as well as in podocytes. In the post‐synapse, MAGI‐2 links the alpha‐amino‐3‐hydroxy‐5‐methyl‐4‐isoxazolepropionic acid (AMPA)‐receptor complex to a variety of proteins and pathways [231], whereas in podocytes it maintains the filter through nephrin signaling complex stabilization [232] and recessive mutations of MAGI‐2 have been identified in patients with steroid‐resistant nephrotic syndrome [233]. Recent interactome studies of the slit diaphragm confirmed that PTPRO, ROBO2, and MAGI‐2 are interaction partners of nephrin and neph1 [15]. Furthermore, this study also identified amyloid precursor protein A4 (APP) and its interactor integral membrane protein 2B (ITM2B) as proteins expressed at the slit diaphragm [15]. In the brain, both proteins are involved in the processing of APP to amyloid‐beta, which aggregates to plaques, one typical hallmark of Alzheimer's disease [234]. The function in podocytes still needs to be clarified.

The deubiquitinating enzyme ubiquitin C‐terminal hydrolase‐L1 (UCH‐L1) is one of the most abundant brain proteins and a key modulator of ubiquitin modification in neurons. Depletion of this enzyme results in neuronal strain and progressive loss of neuronal function, underlining its function in the maintenance of axonal integrity [235, 236]. Upon kidney injury, UCH‐L1 is *de novo* expressed in podocytes and correlates with podocyte ubiquitin content as well as with the internalization of nephrin [237]. UCH‐L1 protein modification in the setting of oxidative stress in podocytes aggravates injury [238]. In summary, podocytes and neurons express many proteins that are specifically found in these two cell types in a high abundance, which frequently fulfill similar functions in the biological context of both cell types, substantiating the power of such comparative analyses for extending our understanding of podocyte biology.

## 3 Developmental Aspects

**FIGURE 11:**
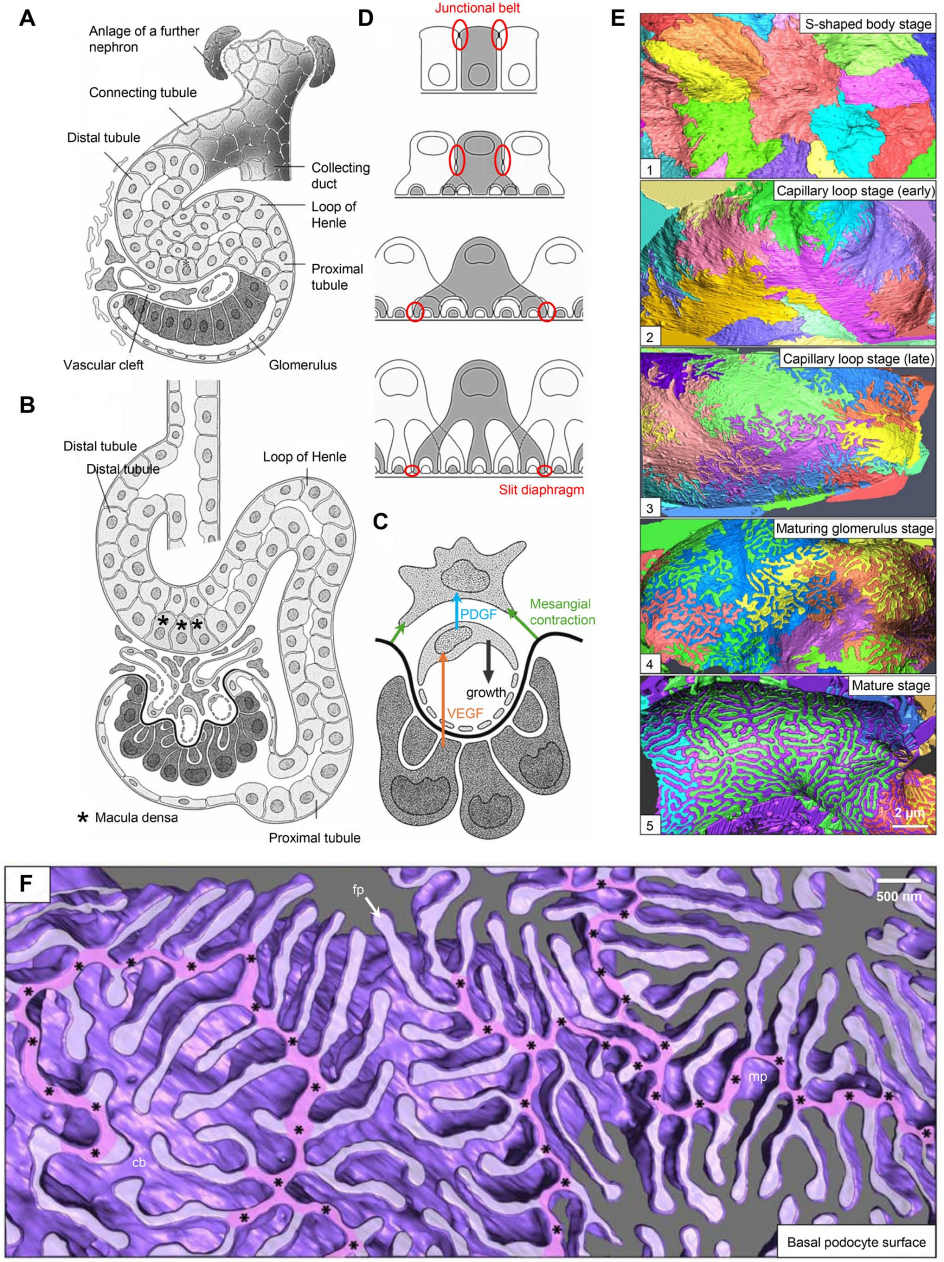
Stages of podocyte development. (A–D) Scheme modified from Kriz [241]: Stages in the development of a renal glomerulus and filtration barrier. (A) The S‐shaped body stage after formation of a distal cleft within the nephron primordium (nephronanlage). The cleft separates the presumptive podocytes from the presumptive macula densa cells and is filled by endothelial and mesangial cells. (B) The early capillary loop stage with beginning formation of a glomerular tuft; the central mechanism in this process. (C) The podocyte endothelial cell‐mesangial cell axis. (D) The four sketches show consecutive stages in the development of podocytes, which start as simple polygonal epithelial cells connected by an apical junctional belt (encircled) and terminate as complexly shaped cells with interdigitating foot processes that are connected by a slit diaphragm. (E, F) Reconstructed serial FIB‐SEM images from rat podocytes, figure modified from Koichiro Ichimura et al. [12]. (E) Basal surface of podocytes. Individual podocytes are shown in different colors and are viewed from the basal side. In the immature glomerulus, it seems that the number of podocytes covering a certain surface area of GBM is almost constant (panels 1–4). In the completely mature glomerulus, the number of podocytes observed in the same surface area is much smaller (panel 5) because the surface area of GBM is extremely enlarged and each podocyte covers a larger area of the GBM. (Panels 1–4) Neonatal rats and (panel 5) adult rats. A similar magnification was used to acquire all images. (F) A single reconstructed podocyte shows the relation between ridge‐like prominences (asterisks) and foot processes (fp). The ridge‐like prominences are observed on the undersurface of the major process (mp) and cell body (cb).

Podocytes originate from the metanephric mesenchymal cells, which undergo several differentiation steps to become podocyte precursors. Traditionally, the development of the glomerulus is morphologically divided into several stages in the mammalian metanephric kidney, including (i) the condensation to generate the nephron primordium (nephronanlage), followed by stages centered around glomerular development, namely (ii) renal vesicular, (iii) comma‐shaped body, (iv) S‐shaped body, (v) capillary loop, (vi) maturing glomerulus, and (vii) mature stages [8, 239, 240] (Figure 11). In the condensation stage, nephrons of the permanent kidney develop from the mesenchymal metanephric blastema by induction through the ureteric bud. The tips of the branching ureteric (collecting) ducts induce the clustering of individual mesenchymal cell aggregates that convert from a mesenchymal to an epithelial phenotype. These cell aggregates undergo many mitotic cycles and differentiation stages, connect with the duct to subsequently generate a nephron.

At the beginning of glomerular development, the first epithelial structure that can be appreciated is the vesicle consisting of polarized cells that are surrounded by a basement membrane. On one side, the vesicle joins with the ureteric bud, and a continuous lumen is formed between the vesicle and the duct. On the opposite side, a cleft appears within the growing nephronanlage, producing a comma‐shaped or S‐shaped body (depending on the section plane, Figure 11A). Here, podocyte precursor cells are first found. These proliferating, simple, polygonal cells form a line along the immature glomerular basement membrane on their basal side, whereas their apical sides are connected to each other through apical intercellular junctions, which resemble tight junctions due to the expression of desmosomal proteins and the first appearance of zonula occludens (ZO)1 [242]. Podocytes now enter the capillary loop stage (Figure 11B), where they begin to establish their characteristic complex cell architecture.

The structural prominence of the podocyte in early nephrogenesis is emphasized by findings pointing to a central role of this cell type in regulating the development of the entire renal corpuscle [8] using essential players such as angiogenic factors in this process (Figure 11C). The development of the glomerular capillaries and mesangium starts from cells that are found within the cleft above the podocyte layer in the comma‐shaped body, which are derived from surrounding mesenchymal cells including sprouts from existing vessels [243]. Several signaling systems are involved in this recruitment and differentiation process, especially the vascular epidermal growth factor (VEGF)‐flk‐1 axis. VEGF is expressed in the podocyte precursor cells of the comma‐shaped body, and its receptor flk‐1 (VEGFR2) is found on endothelial cells in the cleft and adjacent mesenchyme [244, 245]. VEGF not only initiates the penetration of endothelial sprouts into the cleft but is also indispensable for glomerular capillary growth [244, 246], as well as for the maintenance of endothelial fenestrae in adult glomeruli [247]. Furthermore, experiments in podocyte‐specific VEGF‐A knockout mice demonstrated a key role for podocyte VEGF‐A production not only for endothelial development and homeostasis but also for mesangial cell survival and differentiation [248]. Podocytes themselves do not express VEGFR1 or VEGFR2 receptors, but they express neuropilin‐I, a potential coreceptor for the VEGFR2 receptor, which may bind VEGF, thus potentially establishing an autocrine loop or VEGF sequestration [249]. Further podocyte‐secreted regulators of early glomerular capillary development are besides the eph/ephrin family of membrane receptors and counterreceptors (i.e., ephrin‐B2 from podocytes and the counterreceptor eph‐B4 expressed on endothelial cells [250]), the angiopoietins that bind to the endothelial expressed tie‐1 receptor [251]. Podocytes secrete angiopoietin 1, which (together with mesangial cell derived angiopoietin 2) binds to tie‐1. Tie‐1 activation results mainly through angiopoietin 1, hence mesangial angiopoietin 2 is considered an inhibitor of the process [251, 252, 253]. The recruited endothelial cells then commence to produce platelet‐derived growth factor (PDGF)‐BB, which binds to the PDGF receptor expressed by mesangial precursor cells, a signaling axis required for proliferation and assembly of glomerular capillaries and mesangium [254, 255]. Among other functions, the extraglomerular mesangium serves as the ‘punctum fixum’ (anchor), for their contraction permits them to pull on the GBM in a centripetal direction [256]. This creates invaginations of the GBM in between the contact points of capillaries to the GBM, leading to the formation of capillary loops that bulge into the urinary space. The centripetal pull of mesangial cells is most probably regulated by PDGF‐B. Thus, the coordinated stimulation by podocyte‐derived VEGF A and PDGF‐B may represent the key mechanism in development, maintenance and repair of glomerular tuft structure [241].

After the establishment of a glomerular vasculature, signaling events in the opposite direction appear necessary for the final podocyte maturation. Production of GBM components by podocytes and their maturation are marked by the replacement of laminin‐1 with laminin‐11 (consisting of ‐5/‐2/‐1 chains) as well as by the replacement of ‐1, ‐2 chains of type IV collagen by ‐3, ‐4, and ‐5 (type IV collagen) chains characteristic for the mature GBM [257, 258]. Grafting experiments suggest that factors emerging from endothelial cells mediate the switch to laminin‐11 [and possibly also to collagen ‐3, ‐4, ‐5 (type IV)] production in podocytes [259]. The contact site between the capillaries and the podocyte precursor cells represents the anlage for the filtration barrier. At the beginning, the two structures are separated by two basement membranes which later fuse, thus establishing the early three‐layered GBM. During the capillary loop stage, immature podocytes begin to interdigitate with each other at major processes on the basal side, followed by the formation of foot processes. Achieving this basolateral interdigitation of foot processes requires the dramatic growth and extension of the basal portions of immature podocytes (Figure 11D,E) [12]. In the process of interdigitation, the junctional belt continues its movement down along the interdigitating cell processes, and as an important consequence, the apical parts of the interdigitating cell processes change from a basolateral into an apicolateral position, only leaving the sole plates of the foot processes in a basolateral position beneath the junctional belt. Apical membranes thus have free surfaces, which allows them to develop individually into processes of different shapes emerging from the cell body and extending in individual ways towards the foot processes (Figure 11D,E). This mechanism may explain the origin of the different podocyte cell processes, their basal connection as ridge‐like prominences to the GBM (Figure 11F), as well as the lifting of the cell bodies from the GBM, which change into a floating position within the urinary space (see Figure 1C). It is still poorly understood how the splitting of the basal cell portions into processes of increasing delicacy together with the lengthening of the junctional belt is coordinated, as well as how its dramatic change in composition and architecture into a unique membrane‐like intercellular junction, namely the slit diaphragm, is coordinated.

The foot process and slit diaphragm formation at the S‐shaped body stage is characterized by a disappearance of desmosomal proteins [260] and an apical to basal migration of ZO‐1 where the slit diaphragm develops, with the appearance of nephrin [261] and of the major surface protein podocalyxin above the level of the junctional complexes that connect the cells [262]. In conjunction with the appearance of nephrin, the slit membrane‐associated proteins podocin [263] and CD2AP [264] are expressed. With this phenotypic conversion, a loss of mitotic activity is present [265], accompanied by the expression of several other podocyte‐specific proteins, including synaptopodin [221] and GLEPP1/PTPRO [266], and the final intermediate filament protein vimentin [265]. At the capillary loop stage, further podocyte‐specific foot process proteins are starting to be expressed such as THSD7A [202] (see Section 2.6.5).

The developmental process of podocytes is orchestrated by many transcription factor genes which play a major role in early ureteric bud branching [267], as well as genes essential for the conversion of metanephric mesenchymal cells toward renal vesicles such as pax‐2, a mammalian homeobox gene. Renal vesicles are not formed in mouse kidney organ cultures when using antisense oligonucleotides to pax‐2 [268]. Additionally, the further differentiation of these early epithelial cells and their maturation to podocytes correlates with the decrease of pax‐2 and the rise of Wilm's tumor (WT)‐1 expression [269]. The downregulation of pax‐2 appears as a prerequisite to allow podocyte differentiation governed by WT‐1 from the vesicle stage on. This transcription factor remains a specific marker of podocytes during the entire ontogeny and in the adult [270]. Podocyte precursor cells of the S‐shaped body start to express lmx‐1b, a lim homeobox gene whose expression is maintained throughout nephrogenesis and has an essential role for glomerular development, as lmx‐1b knockout mice have podocyte and GBM abnormalities at birth [271]. Pod‐1, a basic helix–loop–helix protein like WT‐1, is expressed in podocytes during glomerular development and appears to be involved in the differentiation of this cell type [272].

## 4 Terminal Differentiation

In contrast to most other renal and non‐renal epithelial cells, the adult podocyte is terminally differentiated and thus unable to proliferate [273]. Several mechanisms underlying this inability have been reported, including factors related to the cellular structure and cell cycle regulatory mechanisms of podocytes. Mature podocytes exhibit an increased expression of cell cycle inhibitors (such as the cyclin kinase inhibitors p27 [274, 275] and p57 [275, 276]), which prevent cell proliferation by inhibiting cyclin‐CDK complexes. These cell cycle inhibitors maintain podocytes in a quiescent state, preventing them from re‐entering the cell cycle. In line, mature podocytes are low in the expression of proliferation markers such as Ki‐67 [277]. Structural constraints originating from the complex podocyte actin cytoskeleton also limit the ability of podocytes to undergo mitosis. As such, it was proposed that a forced re‐entry of podocytes into the cell cycle results in a phenomenon known as mitotic catastrophe [278] (see Section 15).

Terminal podocyte differentiation also originates from a permanent cell cycle exit, that is, by the expression of specific transcription factors and regulators that maintain the specialized function and structure of podocytes. As such, cyclin‐dependent kinase 5 (CDK5), which is involved in maintaining podocyte differentiation and morphology [279], or histone demethylase KDM6B, which is thought to bind the promoter region of Wilms' Tumor 1 (WT1) and reduce the histone H3K27 methylation [280], have been involved in this process. WT1 is crucial for maintaining podocytes in a terminally differentiated state by ensuring the structural and functional integrity of the GFB through its action as a master regulator of podocyte‐specific gene programs by interacting with other transcription factors like foxC1/2 and notch signaling components [281]. WT1 binds nearly all genes known to be crucial for GFB maintenance, that is, key genes involved in podocyte differentiation, such as podocalyxin [282] and nephrin [283] to name a few. Additionally, WT1 modulates key podocyte signaling pathways, including VEGFA and fibroblast growth factor (FGF)2 signaling, by increasing the expression of the 6‐O‐endosulfatases sulf1 and sulf2, which remodel the heparan sulfate 6‐O‐sulfation pattern in the ECM and thus assure the bioavailability of growth factors required for structural/functional stability [284]. WT‐1 is epigenetically regulated. Downregulation of WT1 through microRNA‐193a results in podocyte loss and FSGS in mice [283] and knock‐down of microRNA‐193a in human parietal epithelial cells (and hence upregulation of WT1) results in a transdifferentiation of these proliferative PECs into a non‐proliferative podocyte‐like phenotype [285], together substantiating the central role of this transcription factor for podocyte terminal differentiation.

Finally besides a central involvement of WT1, a further prerequisite for terminal podocyte differentiation is the downregulation of specific transcription factors and regulators that are essential for podocyte development (e.g., notch2, snail, WNT4 [277]). A detailed review of these factors is beyond the scope of this article.

## 5 Podocyte Regeneration

**FIGURE 12:**
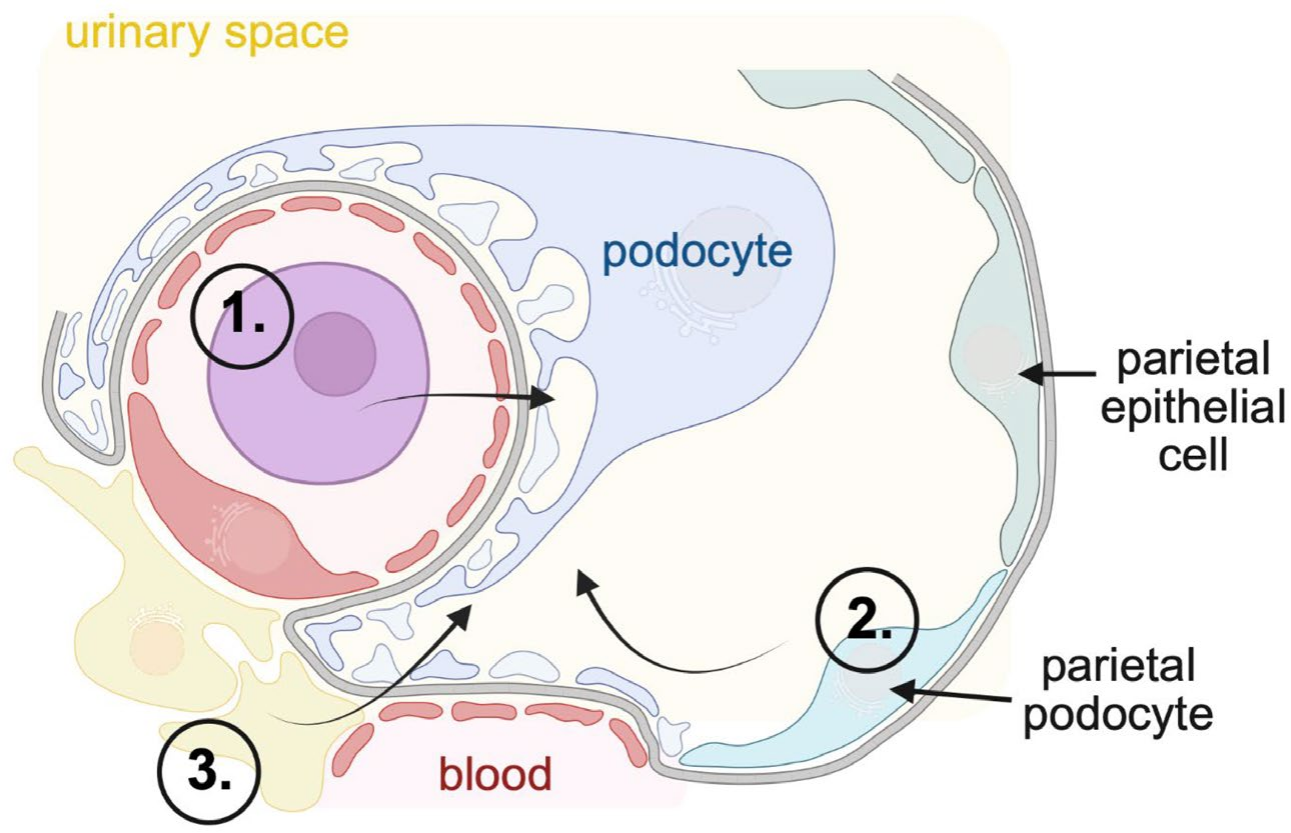
Proposed progenitor niches for podocyte regeneration. Podocyte regeneration in mature kidneys is very limited. Regeneration has been discussed to occur from (1) circulating bone‐marrow derived stem cells, (2) parietal epithelial cells, or (3) cells of renin lineage.

Whether podocytes have a physiologic regenerative capacity and, if so, from which progenitor niche podocyte regeneration might occur is the matter of a longstanding debate. The embryonic progenitor cell that gives rise to podocytes during development does not persist into adulthood in mammalian kidneys. Therefore, podocyte regeneration after organogenesis would need to originate from a different pool of cells [286]. Over the years, circulating bone marrow‐derived cells, parietal epithelial cells (PECs), and cells of renin lineage have been discussed as potential sources for podocyte regeneration (Figure 12; reviewed in [286, 287]). To this end, an involvement of circulating bone marrow‐derived cells could not be substantiated for podocyte regeneration [287, 288].

Experimental approaches propose a role for PECs in podocyte regeneration. The beta‐catenin/Wnt signaling pathway is important during the late stages of nephrogenesis and for the lineage specification of PECs. Deletion of beta‐catenin in tubular cells under the control of the pax8 Cre driver, results in mice in the formation of well differentiated podocytes that replace PECs in Bowman's capsule, substantiating the potential of a PEC‐to‐podocyte conversion [289]. In humans, PECs at the vascular pole of glomeruli express podocyte proteins but do not exhibit the typical ultrastructural features of podocytes and were therefore termed ‘parietal podocytes’ [2]. To investigate, whether PECs contributed to podocyte regeneration, an elegant parietal epithelial cell reporter mouse line for genetic tracing of PECs was introduced in 2009. In this model, β‐galactose was expressed in triple transgenic mice (pPECrtTA/LC1/R26R) in an inducible manner under the control of 3 kb of the human podocalyxin (hPODXL1) flanking region and 0.3 kb of the rabbit Podxl1 untranslated region. Using this mouse a replenishment of podocytes from PECs in the developing mouse kidney could be appreciated [290], but not in the adult kidney [291, 292]. In line, podocyte regeneration in aging murine kidneys could not be detected by combining fluorescent genetic fate mapping (genetically labeled PECs with membrane‐tagged enhanced green fluorescent protein (mG) in inducible hPODXL1.rtTA;tetO.Cre;mT/mG mice) with highly efficient podocyte isolation protocols to precisely quantify podocyte number [293]. So despite the fact that a subset of PECs express progenitor markers CD133 and CD24 [294], and despite the fact that PECs express podocyte transcripts [295] and can differentiate into podocytes in vitro and in vivo [285], a physiologic replacement in adults is most likely limited [286]. During adolescence, PECs could be responsible for the replacement of 10% of podocytes in mice [293, 296], which fits well with human studies showing that the number of podocytes increases by up to 20% early in life [297].

Cells of renin lineage locate alongside glomerular capillaries and have been identified as progenitors capable of differentiating into podocytes and PECs after podocyte injury in mice [286, 298]. These cells are restricted to the juxta‐glomerular compartment in adults, are derived from foxD1‐positive cells [299] and produce (or once produced) renin [300]. Cells of renin lineage have a marked plasticity and can transdifferentiate into smooth muscle cells, mesangial cells and possibly pericytes, and upon *VHL* (von Hippel Lindau) deletion into erythropoietin‐producing cells (reviewed in [286]). Constitutive and inducible reporter mice that permanently tag renin‐producing cells using Cre/loxP somatic DNA recombination demonstrated that cells of renin lineage could serve as adult podocyte stem/progenitors in mice with glomerular injury, especially as they were oftentimes found to co‐express podocyte proteins such as WT1, nephrin, podocin, or synaptopodin in the setting of injury‐induced podocyte depletion [298, 301], and in physiologically aged mice [302]. The replenishment of podocytes by cells of renin lineage was substantiated in reporter mice that allowed to simultaneously fate‐map cells of renin lineage together with podocytes. How cells of renin lineage reach the podocyte location in mice is not clear, as they would need to traverse the glomerular filtration barrier.

In summary, experimental investigations propose a very limited physiologic podocyte replacement in rodents, especially in adolescence; whether this occurs in humans needs to be established.

## 6 How Many Podocytes Do We Have?

Based on seminal work, there is a strong causal relationship between podocyte depletion and glomerulosclerosis, as podocytes have a very low potential for regeneration. In this podocyte depletion hypothesis, the occurrence of absolute podocyte depletion (decrease in the total number of podocytes per glomerulus) as well as relative podocyte depletion (a decrease in the number of podocytes per unit volume of glomerulus) is a direct cause of glomerulosclerosis. In line with this, it was demonstrated that targeted depletion of 0%–20% of podocytes in rats leads to transient proteinuria, mesangial expansion, and normal renal function. Depletion of 21% to 40% of podocytes results in mild persistent proteinuria, mesangial expansion, capsular adhesions (synechiae), focal segmental glomerulosclerosis, and normal renal function. Depletion of over 40% of podocytes results in segmental to global glomerulosclerosis with sustained high‐grade proteinuria and reduced renal function [70]. In this study, targeted podocyte depletion was achieved using a transgenic rat strain in which the human diphtheria toxin receptor is specifically expressed in podocytes. Since the rodent homolog does not act as a diphtheria toxin receptor, rodents are resistant to diphtheria toxin. Injection of diphtheria toxin into transgenic rats but not wild‐type rats results in dose‐dependent podocyte depletion from glomeruli [70].

But what is the “normal” number of podocytes per glomerulus, from where podocyte depletion starts off? To be able to count podocytes in an accurate (hence unbiased) and precise (low variance) manner has proven to be surprisingly difficult and controversial [303]. Over the last 30 years, different methods to assess podocyte number have been implemented [303], ranging from counting podocyte number per glomerular cross section [304], to model‐based stereology [305], FACS‐based podocyte counting [293], or design‐based stereology [297]. The latter (very time‐consuming) approach is applicable in every species and determines podocyte density through the combination of immunohistochemistry, confocal microscopy, and design‐based stereology, thus enabling the assessment of individual glomerular volume and the corresponding podocyte numbers, among other parameters. By using this approach, it could be demonstrated in human autopsy kidneys that glomeruli from children were small and contained around 450 podocytes per glomerulus, whereas adult glomeruli were larger and contained significantly more (around 550, range 263–983) podocytes per glomerulus [297], raising questions about the postnatal origin of these additional podocytes. Despite an increased number of podocytes, large adult glomeruli had lower podocyte densities than smaller glomeruli, indicating that these large glomeruli had relative podocyte depletion, possibly placing them at greater risk of subsequent pathological change [297]. The number of podocytes per glomerulus is significantly lower in mice and ranges from 70 to 80 podocytes per glomerulus, as determined by using an approach that combines immunofluorescence, optical clearing, confocal microscopy, and three‐dimensional analysis [306]. Since glomerular volume is also lower in mice compared to humans, podocyte density does not differ between both species.

## 7 Podocyte Motility

**FIGURE 13:**
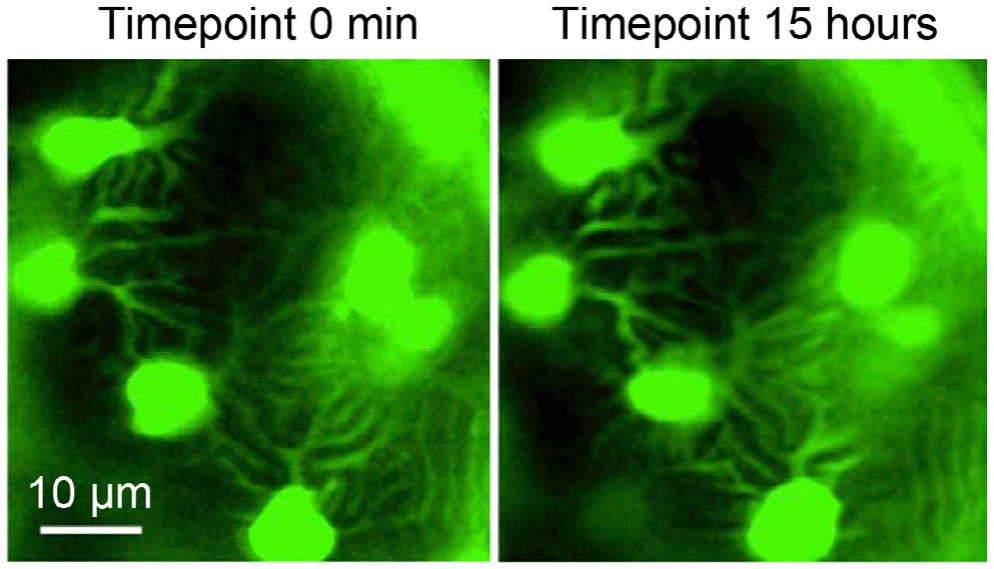
Podocyte processes can be visualized in vivo over time in zebrafish larva. No dynamics of podocyte processes are present in eGFP‐labeled podocytes recorded in translucent zebrafish larva for 15 h via two‐photon microscopy. Images were taken every 30 min. No notable changes in the position of the primary and secondary processes were observed. Modified from Endlich et al. [125].

As detailed in the paragraphs above, podocytes in vivo and in vitro dispose of all necessary molecular components for movement, that is, an actin cytoskeleton associated with actin polymerizing and nucleating factors, as well as actin cytoskeleton regulating enzymes such as the small rho GTPases rho and rac [307]. It has long been discussed whether healthy podocytes are motile in vivo and/or whether they can become motile after exposure to specific stimuli. If podocytes were to be motile, which kind of motility would they be able to perform? Would they be able to migrate substantially along the glomerular capillaries in the x‐, y‐ and z‐direction (translocative motility), or would motility be reduced to small, confined movements of foot processes (stationary motility)? With the implementation of novel microscopic techniques like multiphoton microscopy, several groups have set out to answer the question of podocyte motility [308]. Initial observations by Peti‐Peterdi and Sipos in intravital two‐photon microscopy suggested that most podocytes in healthy mice do not show translocative motility. However, some selected podocytes exhibited the propensity to move on the glomerular tuft in the setting of injury [309]. Whether these moving podocytes represented activated podocytes due to some injurious signal was not dissectible in the approach, as the observed motile podocytes could only be identified through negative staining within Bowman's space following the intravenous injection of fluorescent tracers that reached Bowman's space through free filtration and subsequently initiated a positive labeling of the lumen of Bowman's capsule. Long‐term in vivo multiphoton microscopy of eGFP‐labeled podocytes in the zebra fish model by the Endlich group substantiated the notion that podocytes are static cells and show no translocative or stationary motility (Figure 13). As such, no changes in the branching pattern of podocyte major processes were discernible over the continuous imaging time course of up to 23 h in fish larvae, and no migratory movement of podocytes was seen [125]. These findings were further substantiated by intravital and kidney slice two‐photon imaging of the three‐dimensional structure of mouse podocytes, which demonstrated that also in the mouse uninjured podocytes remained nonmotile and maintained a canopy‐shaped structure over time [124]. Intriguingly, upon expression of constitutively active rac1, podocytes, however, can change shape by retracting processes and exhibit domains of increased membrane activity. Constitutive activation of rac1 also leads to podocyte detachment from the glomerular basement membrane, and detached podocytes were found to crawl along the surface of the tubular epithelium and to transmigrate into the interstitium [124]. Summarizing the findings of the past years, unchallenged podocytes that cover the capillary network with their elaborate cytoskeleton represent cells with little to no motility. They, however, have the propensity for stationary as well as translocative motility in pathophysiologic settings resulting in foot process effacement and apical lammelipodia generation (i.e., in response to proheparin‐binding EGF‐like growth factor (HB‐EGF) [310]), up to detachment from the GBM and loss.

## 8 Podocyte Function

**FIGURE 14:**
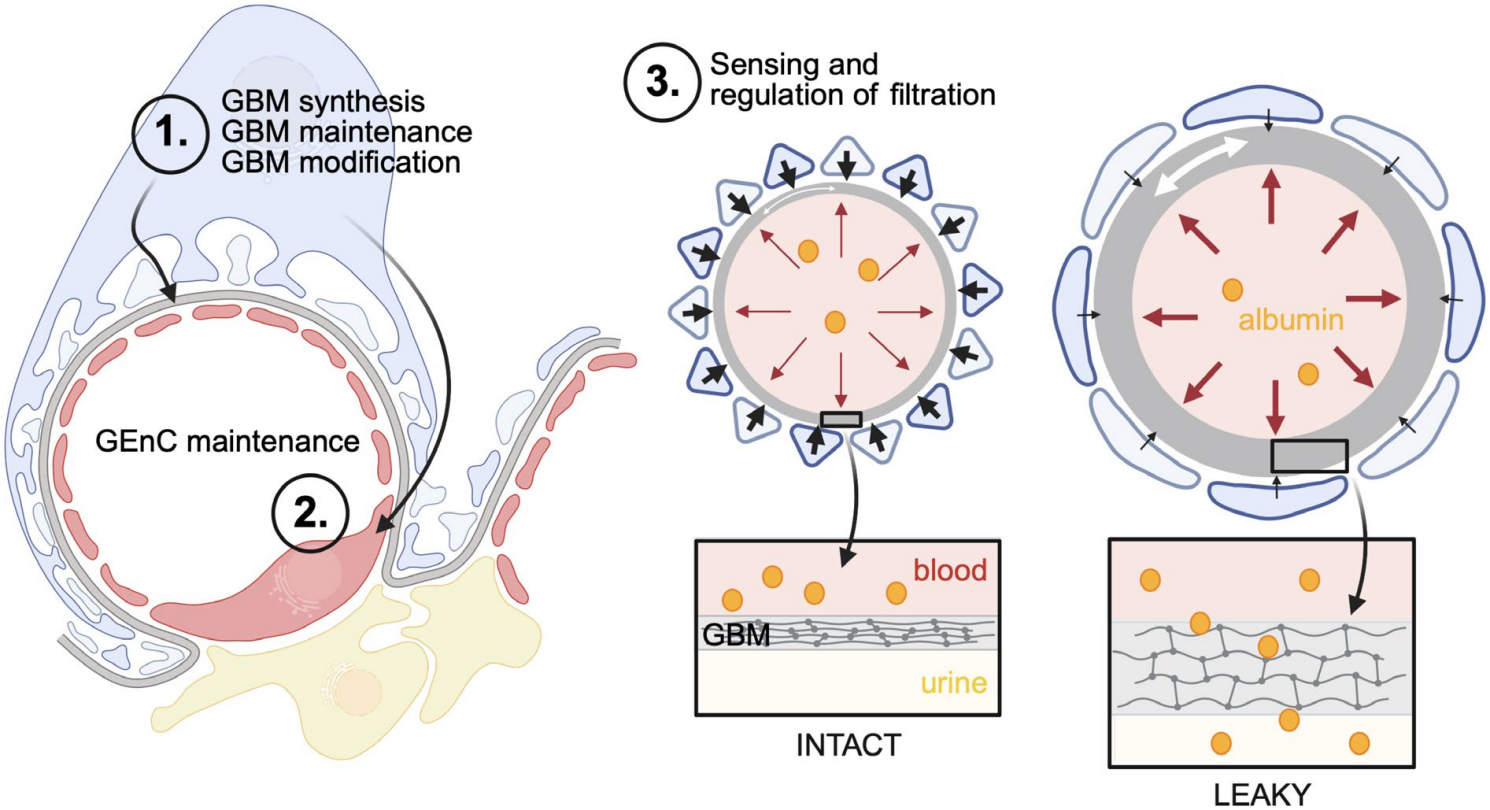
Podocyte function. Scheme depicts the main functions of podocytes in (1.) the synthesis of the GBM in cross‐talk with the glomerular endothelial cells (GEnCs), and in the maintenance and modification of the GBM; in (2.) the maintenance of GEnCs through the secretion of, that is, VEGF; and in (3.) the sensing and regulation of filtration.

The mature podocyte is essential for the homeostasis of the glomerular basement membrane and glomerular endothelial cells and for the sensing and regulation of filtration (Figure 14).

### 8.1 Synthesis and Maintenance of Glomerular Basement Membrane

The GBM between endothelial cells and podocytes not only provides a scaffold for the capillaries, it is also an essential part of the glomerular filtration barrier (GFB). In the mature kidney, this three‐layered barrier is mainly built, maintained, and regulated by podocytes. The GFB is important for retaining macromolecules and cells within the circulation; however, it enables the passage of water and small molecules into the urine. Dysfunction of the GFB results in proteinuria and hematuria [311]. Compared to other basement membranes, with a width of 330–460 nm in humans [312] and 50–300 nm in rodents [313, 314], the GBM is rather thick, and its composition was a mystery for a long time. Mass spectrometry‐based proteomics revealed as major components type IV collagen, collagen I, and laminin isoforms [315], secreted by podocytes and endothelial cells.

The synthesis of the GBM requires the cross‐talk of podocytes with endothelial cells [316] and varies depending on its developmental stage (see Section 3). In mature glomeruli, the final laminin 521 isoform is secreted by both cell types, whereas only podocytes produce type IV collagen alpha3alpha4alpha5 of the fully mature GBM [311, 317]. Further components of the GBM are nidogens and heparan sulfate proteoglycans (HSPGs), both linking the laminin and collagen network. The HSPGs agrin, perlecan, and type XVIII collagen are further considered to charge the GBM negatively [318]. Agrin, the only HSPG secreted by mature podocytes, stabilizes the GBM by binding to laminin‐gamma1 and enables cell‐matrix adhesion and signaling by binding to cell‐surface receptors like integrin alphavbeta1 [319], substantiating the importance of post‐developmental GBM synthesis by podocytes.

Besides persistent GBM synthesis, adult podocytes modify the GBM. It was recently shown that type IV collagen is post‐translationally modified by enzyme prolyl 3‐hydroxylase 2 (P3H2), which catalyzes 3′ hydroxylation of proline residues of type IV collagen leading to network stability [320]. Mice with podocyte‐specific knockout of P3H2 develop thin basement membrane nephropathy, which finally leads to focal segmental glomerulosclerosis confirming observations of patients harboring *P3H2* mutations [321] and suggesting a permanent activity of podocytes in GBM modification. Another interesting aspect is the permanent regulation of the GFB by the intrinsic circadian clock.

Podocytes stabilize the GBM through a combination of strong cell‐matrix adhesion, structural support via contractile foot processes, and dynamic interactions with the GBM. Adherence to the GBM occurs mostly through integrins of the adhesome (see also Section 2.4), which connect the ECM to the foot process actin cytoskeleton and by this provide mechanical stability. Specifically, the interaction of integrin alpha3beta1 with laminin alpha5 is important for the assembly of laminin sheets, in which the N‐terminal arms of the laminins enable polymerization [311]. This laminin polymerization is dependent on laminin beta2 (encoded by *LAMB2*) [322]. Involvement of integrin alphavbeta3 as an inducer of the urokinase receptor (uPar) signaling leads to increased podocyte motility and activation of GTPases. However, by abnormal integrin activation, uPar can be a pathogenic mediator of FSGS [323]. Further cell‐adhesion receptors essential for GBM stabilization are the integrins alpha2beta1, alpha‐dystroglycan, syndecan‐4, and type XVII collagen [92], as mutations in these proteins have serious consequences for the stability and integrity of the GBM. Besides the above‐mentioned adhesion receptors, GBM stabilization is also obtained by the contractile foot process elements that stabilize the GBM by counteracting local elastic distension (see also Section 2.5, Figure 14 point 3). The interaction of podocytes with the GBM is dynamic, assuring an adaptation to stress conditions such as fluid shear stress by modulating the expression of proteins like fibronectin, which contribute to podocyte stability [324]. Especially, the synthesis of minor type IV collagen by podocytes is essential for GBM stability as it assembles into large structures (supercoils) that contribute to the mechanical stability of the GBM collagen network as a whole [325]. In support, different human mutations in type IV collagen alpha3alpha4alpha5 lead to their absence, resulting in Alport's disease, which is characterized by a failure of the mature GBM network to properly assemble with persistence of type IV collagen alpha1alpha2alpha1. Consequently, the GBM is multilayered, resulting in proteinuria [326, 327].

Transcriptomic analysis at six different timepoints across one circadian cycle of isolated glomeruli with podocyte‐specific inactivation of intrinsic circadian clock genes revealed altered expression of several proteins important for podocyte function. On the other hand, proteins regulating podocyte and GBM architecture like cathepsin L [328] and G protein‐coupled receptor class C group 5 member A (Gprc5a) [329] or proteins involved in the communication between podocytes and GEnCs like sulfatase 2 [284], show an upregulated transcription. Bulk‐sequencing analysis revealed 82 rhythmically expressed genes important for GBM function and structure like collagens, proteoglycans, and secreted factors [330]. However, it still needs to be determined if this rhythmic transcription mentioned above also results in rhythmic expression of proteins. In 2019, a dependence of the glomerular filtration rate (GFR) on circadian rhythmicity in podocytes could be shown by using a podocyte‐specific BMAL1 (brain and muscle arnt‐like protein‐1) deficient mouse model. BMAL1 is an important component of the mammalian circadian clock, which results in arrhythmia when dysfunctional [331]. Further, podocytes exhibit circadian‐dependent altered interaction with the GBM and significantly changed protein secretion [332]. Taken together, the GBM is a complex cellular matrix with highly dynamic proteins to guarantee proper blood filtration [13].

### 8.2 Maintenance of The Glomerular Endothelial Cells

Podocytes maintain the GFB by secreting different survival factors especially for the endothelial cells residing ‘vis‐à‐vis’, some of those will be discussed in the following (see also Sections 3 and 10.1). Angiopoietin‐1 (ang‐1) binds to the tyrosine kinase tie‐2, which is expressed by glomerular endothelial cells (GEnC) and mesangial cells, thereby enhancing survival and cell–cell stabilization [333]. Together with VEGF‐A, which binds to VEGFR2 on GEnCs [334], ang‐1 is not only essential for the proper development but also for maintenance of adult vasculature [335]. Glomerular expression of ang‐1 was shown to be reduced in a model of glomerulonephritis, while expression of ang‐2 was increased, proofing the natural antagonism of ang‐2 on ang‐1. This ang‐1/ang‐2 balance correlated with GEnC apoptosis and downregulation of VEGF‐A [336, 337]. Another podocyte‐secreted factor of potential importance for the survival of GEnCs in mature glomeruli is plasma angiopoietin‐like 4 (angptl4). Normosialylated angptl4 binds to integrin alphaVbeta5 on GEnC and was shown to thus decrease the risk of proteinuria [338]. Knockdown of angptl4 in association with high fat diet was shown to ameliorate hyperlipidemia‐induced renal injury. This observation could be explained by an increased podocyte expression of alpha‐actinin‐4 (ACTN4) [339]. The homeostatic chemokine CXCL12, also known as Stromal derived factor 1 (SDF1), is secreted by podocytes adjacent to endothelial cells expressing its receptor CXCR4. The CXCL12/CXCR4 axis regulates vascular development in the kidney [340], and contributes to glomerular endothelial injury when this axis is dysfunctional mature kidneys [341].

### 8.3 Sensing and Regulation of Filtration

Comparative analyses to *C. elegans* suggest that podocytes can regulate glomerular filtration by sensing the glomerular filtration pressure through a podocin‐based mechanoreceptor complex situated at the slit diaphragm [5]. In *C. elegans* , the protein MEC‐2 is part of a multiprotein channel complex that transduces sensation of gentle touch [342]. MEC‐2 is the closest homolog of podocin and both podocin and MEC‐2 are PHB‐domain proteins with cholesterol‐binding properties [33]. In *C. elegans* , this sterol binding of MEC‐2 is required for touch sensitivity [33], suggesting that similar processes are at work at the podocyte slit diaphragm. In line, the interaction of podocin with TRPC6 could be shown in Xenopus oocytes, an interaction that affects the activity of the TRPC6 channel, a sensor of mechanically and osmotically induced membrane stretch [343]. In Xenopus oocytes, TRPC6 activity is dependent on the cholesterol binding capacity of podocin [33]. While this concept is compelling, in vivo proof of podocyte mechanosensation is challenging.

Podocytes are thought to regulate glomerular filtration. Based on the dependence of solute partitioning and filtration on different extracellular matrices, the compression of the GBM as a ‘gel‐like’ structure is thought to represent a mechanism that reduces the permeability of the filtration barrier to macromolecules [4]. These findings suggest a physical mechanism that couples podocyte structure to permeability characteristics of the GBM. Recent morphometric super‐resolution data of podocyte foot process morphology prior to and after onset of albuminuria in mice expressing human relevant podocin mutations combined with mathematical modeling support this concept [23]. As such, podocytes might achieve GBM compression (also called buttress force) by podocyte foot process adhesion to the GBM and through the tensile forces of their foot process cytoskeleton [3]. Supporting, filtration pressure increases induced through angiotensin‐2 infusion could not be counteracted in mice expressing mutant podocin in podocytes (hence podocytes with effaced foot processes), whereas wild type mice with intact foot processes (hence intact buttress forces) could counteract angiotensin‐2 related filtration pressure rises [23]. This buttress force represents rather a mechanical stability than a contractile force provided by foot processes.

## 9 Podocytes and the Immune System

Evidence is emerging that podocytes play a role in innate and adaptive immunity. Especially recent investigations in *Drosophila* nephrocytes suggest an immune modulatory propensity of podocytes [344, 345].

### 9.1 Podocytes in Innate Immunity

Innate immunity is the first line of defense against pathogens, which most organisms possess from their origin, and encompasses mechanical barriers (such as skin, mucus) as well as the complement system, neutrophil response, and cell communication through various cytokines. Innate immunity relies on the recognition of pathogens through different receptors, which then triggers a response to destroy the pathogen (reviewed in [346, 347]). Multiple ways have been described by which an organism can sense danger: by receptors recognizing molecular patterns or pathogen‐associated molecular patterns as well as danger/damage‐associated molecular patterns [346, 347]. Toll‐like receptors (TLRs), as part of the pattern‐recognition receptor family, in general recognize microbial and viral membrane components and nucleic acids. Podocytes have been found to express RIG1‐like helicase (RLH) as well as TLRs. Especially TLR4, which can recognize bacterial lipopolysaccharides, but also TLR3, which is responsible for the recognition of viral dsRNA, are expressed by podocytes, suggesting that podocytes play a role in innate immunity [348, 349] and can combat at least microbial and viral enemies. Further hints to a role of podocytes in innate immunity are the expression of functional chemokine receptors in cultured human podocytes [350]. Chemokines are small cytokines that are released by innate immune cells. Chemokines play a substantial role in inflammation and immune cell recruitment by guiding circulating leukocytes to inflamed or damaged sites. Podocytes in culture as well as histologically within kidney biopsies from patients with primary membranous nephropathy express the chemokine receptors CXCR1, CXCR3, and CXCR5 [ 350]. Innate immune signaling pathways are present and can be activated in podocytes. Whether the receptor‐mediated innate immune response furthers podocyte health or causes more harm to podocytes is debatable. It has been described that the innate immune response, when activated in podocytes, leads to injury [351]. Reiser et al. suggest that podocytes sense the bacterial lipopolysaccharide (LPS) by TLR4, which induces the costimulatory molecule B7‐1 (CD80), leading to proteinuria [352], while TLR3 and RLH signaling in podocytes leads to structural and functional changes of the filtration barrier [353]. Recently, in the discovery frenzy caused by the COVID pandemics 2020–2023, an interaction of SARS‐CoV2 with the soluble urokinase receptor (suPAR) as part of innate immunity was described, potentially representing an explanation for viral response proteinuria [354].

Another integral part of the innate immune response is the complement system, whose activation leads to opsonization and lysis of pathogens [355]. Complement activation can be achieved via three different pathways: the classical, lectin, and alternative pathways. The classical pathway is initiated by binding of C1q to antigen–antibody complexes, leading to cleavage of C2 and C4, which enables assembly of the classical/lectin C3 convertase C4bC2b. The lectin pathway is activated by pattern recognition molecules such as mannan‐binding lectin (MBL), which bind to MBL‐associated serine proteases (MASPs). This subsequently induces cleavage of C2 and C4 and the formation of the classical/lectin C3 convertase C4bC2b. The alternative pathway is initiated by slow hydrolysis of C3, which in the presence of complement factors B and D, leads to the formation of the alternative C3 convertase C3bBb. All pathways result in the subsequent cleavage of C3, which leads to the tagging of the foreign pathogen, enabling specific pathogen clearance [356]. The cleavage of C3 can then either lead to an amplification of C3 cleavage or to inactivation of the cleaved product. Complement activation therefore participates in the clearance of apoptotic cells and immune complexes [357]. Podocytes are both the source and the target of complement‐mediated injury. Podocytes express multiple complement components, such as complement receptor type 1 (CR1) and type 2 (CR2) as well as complement regulators like CD56, CD55, and CD59 [358]. Podocytes also produce complement proteins such as complement component 3 (C3) and complement factor H (CFH) [359]. These studies suggest that podocytes can activate and regulate a local glomerular complement system. Podocytes are, however, also targets of the complement system by expression of CR1 and CR2, as well as C3a and C5a receptors [358].

### 9.2 Podocytes in Adaptive Immunity

The adaptive immune system enables specific antigen recognition and the development of an immunologic memory. Adaptive immunity relies on the regulated interplay of antigen presenting cells (APCs), T‐, and B‐lymphocytes [360]. The adaptive immune response is initiated by the stimulation of naïve T‐lymphocytes by APCs [361]. APCs present peptides bound to the surface of APCs via major histocompatibility complexes (MHC). The two main subsets of T‐lymphocytes associated with adaptive immunity are cytotoxic T cells and T helper cells. Cytotoxic CD8+ T cells can be activated by the presentation of antigens derived from the products of intracellular pathogens or of self‐proteins bound to MHC class I molecules. CD4+ helper T cells can be activated by the presentation of peptide fragments derived from extracellular proteins (internalized by endocytosis and subsequently degraded intracellularly) bound to MHC class II. Alternatively, antigens derived from endocytic uptake can also be presented on MHC class I (cross‐presentation) to activate CD8+ T cells [362]. Activated T‐cells either directly kill infected target cells or enhance the immune response by triggering cytokine release and thereby stimulating a T and B cell response [360]. Whether and in which way podocytes play a role in adaptive immunity is a matter of debate. Under inflammatory conditions podocytes upregulate the expression of MHC class II, which suggests that podocytes have the capability to present antigens to the immune system. The group further showed in vitro and in vivo that podocytes actively participate in adaptive immunity by firstly expressing MHC class I and II and by being able to activate specific T cell responses [363]. That podocytes take an active role in immunity is highly controversial, but as immunosuppression is the standard therapy for a multitude of renal diseases, it is possible that infiltrating immune cells attracted by podocyte‐released factors or extracellular vesicles are involved in disease development.

Various kidney diseases are associated with periglomerular immune cell infiltrates such as macrophages, DCs, and T cells [364, 365, 366] and for many decades it was not clear whether podocytes could be targeted by immune cells. In an elegant study, the impact and access of CD8+ T cells to podocyte targeting was investigated [367]. In the mouse model used, CD8+ T cells expressed a T cell receptor that specifically recognized EGFP (Jedi mouse). Thereby, podocyte‐specific EGFP‐expressing mice injected with Jedi T cells should exhibit podocyte targeting by podocyte‐specific cytotoxic T cells. Under physiologic conditions, however, podocytes were protected from the Jedi T cells by Bowman's capsule. As soon as Bowman's capsule was disrupted by the additional application of nephrotoxic serum, injected Jedi T cells were able to access the glomerular tuft and target EGFP‐expressing podocytes, ultimately leading to massive podocyte loss [367]. These investigations substantiate that podocytes reside within an immunologic protected niche and hence only directly interact with T lymphocytes and the immune system if Bowman's capsule is disrupted.

The current state of investigations suggests that podocytes can influence and alter the immune system and response in a multitude of ways, whether directly or indirectly still requires investigation. Many discoveries in this area were made in cultured podocytes; therefore, further studies are necessary to elucidate in what way podocytes influence the immune system under physiological and pathophysiological conditions.

## 10 Podocyte Cross‐Talk

**FIGURE 15:**
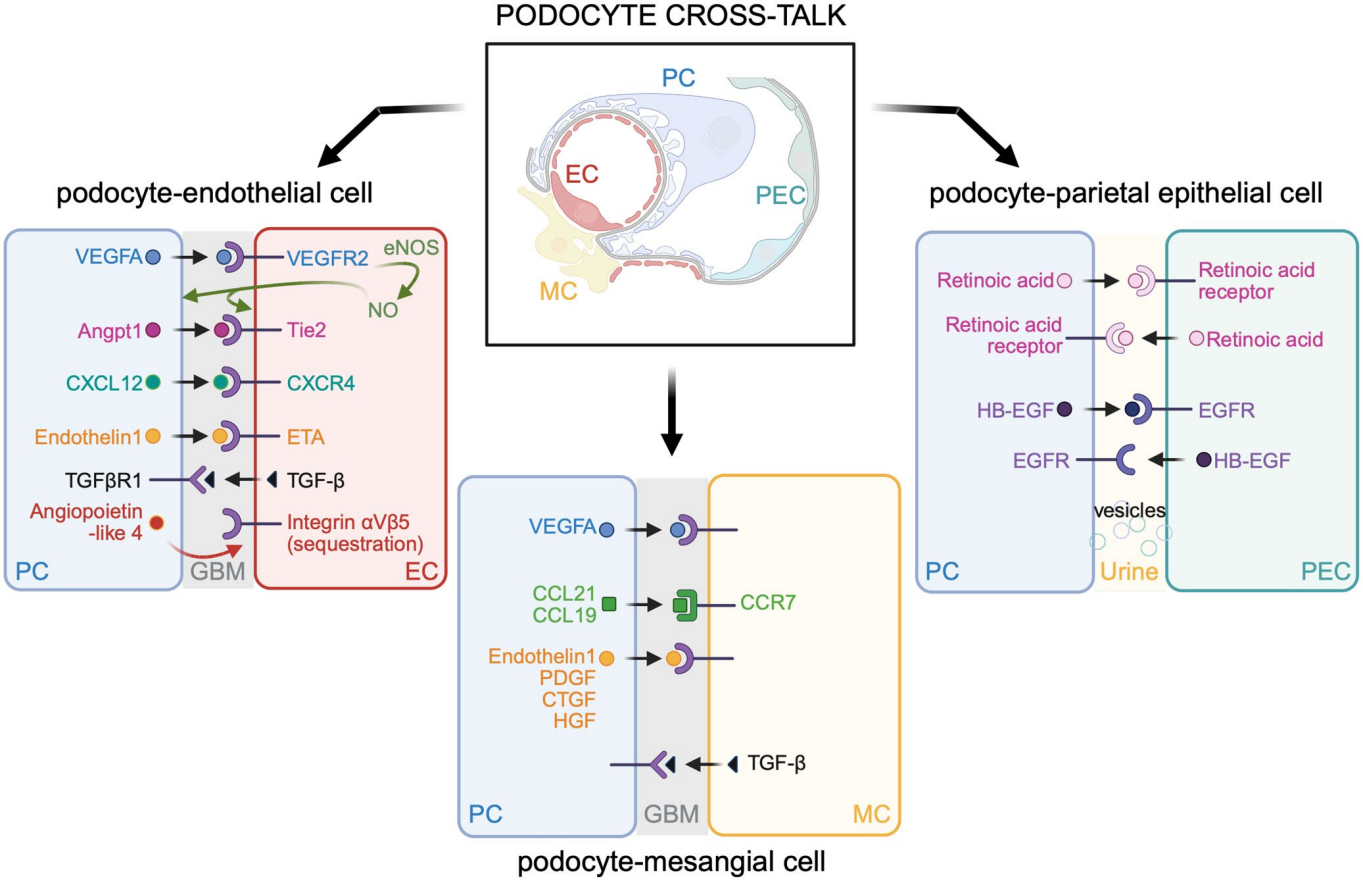
Podocyte cross‐talk. Scheme depicting the molecular cross‐talk routes described for podocytes (PC) with glomerular endothelial cells (EC), parietal epithelial cells (PECs) and mesangial cells (MC). The ligand and receptor pairs are highlighted with the arrows indicating the direction of signaling. Angpt1 = angiopoietin 1; CCL21/19 = CC motif chemokine ligand 21/19; CCR7 = CC motif chemokine receptor 7; CTGF = connective tissue growth factor; CXCL12 = Stromal Cell‐derived Factor 1 (SDF‐1); CXCR4 = C‐X‐C chemokine receptor type 4; EGFR = epidermal growth factor receptor; eNOS = endothelial nitric oxide synthase; ETA = Endothelin receptor A; GBM = glomerular basement membrane; HB‐EGF = heparin‐binding EGF‐like growth factor; HGF = hepatocyte growth factor; NO = nitric oxide; PDGF = platelet‐derived growth factor; TGF‐β = transforming growth factor β; TGFβR1 = transforming growth factor β receptor 1; tie2 = tyrosine‐protein kinase receptor; VEGFA = vascular endothelial growth factor A; VEGFR2 = vascular endothelial growth factor receptor 2 (Flk‐1).

For a long‐time, isolated cell‐type based research in glomerular biology was in focus; lately, this has shifted to a more systemic approach acknowledging the fact that proper glomerular cell function relies on the interplay of the individual cell types. It is notable that primary injury of one glomerular cell type affects the other glomerular cell types, and that cross‐talk of these cells is instrumental for normal glomerular development and health. Podocytes have been shown to communicate with all resident cells of the glomerulus (Figure 15), as summarized in the following.

### 10.1 Podocytes and Glomerular Endothelial Cells

The first glomerular cell cross‐talk identified was between podocytes and glomerular endothelial cells through the secretion of the vascular growth factor VEGFA by podocytes, which binds to its receptor VEGFR2 expressed on GEnCs. This communication was shown to be imperative for the health of GEnCs [368]. Following this seminal discovery, further cross‐talk axes between podocytes and GEnCs were described. The secretion of other podocyte proteins such as the transcription factor 1 (Pod1/Tcf21) [369] or the transforming growth factor (TGF)‐beta activated kinase 1 (Tak1) [370] have also been shown to be essential for GEnC development and health, but it remains to be determined if this is a direct effect or an indirect effect of altered VEGFA levels. Another important podocyte‐GEnC communication system is the nitric oxide (NO) stimulated pathway, which not only plays an important role in blood pressure regulation and calcium homeostasis but was also shown to regulate SIRT1‐AMPK signaling and glucose uptake in podocytes [371, 372]. GEnCs synthesize NO through endothelial nitric oxide synthase (eNOS), a pathway stimulated not only by shear stress but also downstream of podocyte‐derived VEGFA binding to the VEGFR2 on GEnCs. Altered eNOS activation in GEnCs results in lower NO production and subsequent GEnC injury [373], which also affects podocytes. Further, GEnC‐derived NO feeds back to podocytes, influencing slit diaphragm integrity and actin cytoskeleton stability. As such, exposure of cultured podocytes to conditioned medium derived from GEnCs deficient in eNOS and exposed to high glucose and angiotensin II causes RhoA activation and structural changes [374]. Therefore, the control of VEGFA levels is important for GFB function. The expression of another vascular growth factor, ephrin B2, by podocyte progenitor cells could contribute to the expression of ephB4 receptor on GEnC, thereby affecting their development and health [250]. Podocytes (as well as mesangial cells) express angiopoietin‐1 (angpt1) which binds to the tyrosine‐protein kinase receptor (tie2/tek) expressed on GEnC, leading to a stabilization of the glomerular capillaries. Mice with an induced deletion of angpt1 at embryonic day 10.5 show dilated capillary loops and disrupted GBM structures and reduced levels of mesangial cells, while podocytes appear intact [375]. Podocytes also secrete the chemokine CXCL12 (SDF1), which binds to its receptor CXCR4 on GEnC, a cross‐talk important for the formation of glomerular capillaries [340]. Podocytes are thought to protect GEnCs from oxidative injury through the secretion of angiopoetin‐like‐4 (angptl4). Angptl4 is structurally related to angiopoietins but does not signal over tie2. Angptl4 protects GEnC from oxidative injury in nephrotic syndrome by binding to integrins alpha5beta5 [338]. The secretion of vasohibin by GEnCs in turn protects podocytes by counteracting VEGFA signaling in situations of pathologically increased VEGFA levels such as were observed in diabetic nephropathy [376].

In addition to the importance of podocyte‐derived signals for the development and maintenance of GEnCs, podocytes also secrete signals that exacerbate or attenuate GEnC injury in disease settings. Endothelin‐1 binds in a paracrine way to the endothelin receptor A (ETA) on GEnCs. Enhanced podocyte expression and subsequent circulation of endothelin‐1 mediates mitochondrial oxidative stress and dysfunction in adjacent GEnC [377, 378]. In diabetic nephropathy, the endothelin‐1 dependent release of heparanase by podocytes, an enzyme that degrades the glycocalyx of GEnCs, contributes to GEnC injury [379]. In the high glucose environment of diabetic disease, GEnCs in turn cause podocyte dysfunction through increases in TGF‐β1 expression and activation of the Wnt/beta‐catenin signaling pathway [380]. The leucine‐rich alpha‐2‐glycoprotein 1 (LRG‐1) mediates protein interaction and signal transduction [381]. LRG‐1 is mainly expressed in GEnCs and not in podocytes and promotes the progression of diabetic kidney disease by enhancing angiogenesis through TGF‐β/ALK1 [382]. The ablation of LRG‐1 in diabetic kidney disease reduces not only glomerular angiogenesis but also attenuates foot process effacement, podocyte loss, and mesangial expansion, suggesting a role of LRG‐1 in the cross‐talk between GEnCs and podocytes [383]. The aforementioned CXCL12/CXCR4 cross‐talk enhances GEnC injury in diabetic nephropathy [384] and in shiga toxin‐associated hemolytic uremic syndrome [385]. A disease promoting expression of angiopoietins by podocytes is mostly the protein angiopoietin‐2, which leads to GEnC apoptosis without podocyte damage [386]. The effect of metabolic challenges, such as hyperglycemia or exposure to methylglyoxal as happens in diabetes, on podocyte and GEnC gene expression was analyzed in a podocyte and GEnC coculture experiment. These experiments revealed that metabolic challenges change gene expression in cocultured podocytes and GEnC differentially when compared to monocultured cells. Interestingly, different gene families react to metabolic challenges in the different cell types [387].

As more and more knowledge about the role of extracellular vesicles in cellular communication becomes available, the communication between podocytes and glomerular endothelial cells via EV‐microRNA could be shown in an in vitro co‐culture approach. Podocytes take up GEnC derived EVs upon LPS stimulation, and the amount of GEnC derived micro‐RNA in podocytes increased after LPS stimulation. Alterations in podocyte gene expression were observed after treatment with GEnC derived EVs [388].

### 10.2 Podocytes and Mesangial Cells

There is a multitude of evidence supporting communication between mesangial cells and GEnCs, especially under metabolic challenges, while information regarding podocyte–mesangial cell communication is scarce. So far, only indirect evidence exists suggesting that podocytes and mesangial cells influence each other's well‐being. In patients with congenital nephrotic syndrome of the Finnish type, caused by mutations in the podocyte slit diaphragm protein nephrin, evidence of mesangial dysfunction is present, such as mesangial expansion [389]. Furthermore, mutations in other podocyte genes, such as the transcription factor POD1/TCF21 [390], phospholipase Cε1 [391], laminin α5 [392], and Wilms' tumor antigen [393], result in a failure of mesangial cells to migrate into glomeruli during glomerular development. Another factor suggesting relevant podocyte–mesangial cell cross‐talk in glomerular development is that decreased podocyte VEGFA secretion results in mesangiolysis [248], while podocyte‐specific overexpression of VEGF also leads to mesangial cell loss, possibly through affected PDGF receptor beta‐mediated signaling [394]. Podocyte‐derived signaling also seems to affect mesangial cell adhesion and signaling at the GBM, as the podocyte‐specific deletion of collagen type 4 alpha3 (Alport mouse) leads to increased expression of integrin alpha1 in MCs [395]. Another indicator that podocyte‐derived signaling regulates mesangial cell migration and adherence to the GBM is the generation of the chemokines CCL19 and CCL21 by podocytes, which bind to CCR7 on mesangial cells [396]. Under pathological conditions in a podocyte‐specific CCN2 transgenic diabetes mouse model, podocyte‐produced CCN2 (also known as CTGF or connective tissue growth factor) inhibits the degradation of the extracellular matrix formed by mesangial cells, subsequently leading to the accumulation of mesangial matrix and glomerulosclerosis [383]. In vitro experiments with podocytes and mesangial cells also hint at a cross‐talk, as mesangial cells cultured under high glucose conditions suppress the endoplasmic reticulum‐associated protein degradation (ERAD) pathway in podocytes and induce podocyte apoptosis [397]. A multitude of pathways might be involved in cross‐talk between podocytes and mesangial cells in pathologic conditions, such as endothelin 1, PDGF, CTGF, HGF, and TGF‐beta [398]. Whether or not these pathways are affected by direct podocyte–mesangial cell cross‐talk or if mesangial cells are indirectly affected by altered PDGF‐B levels resulting from podocyte‐dependent GEnC signaling is unclear. Further investigations are necessary to determine the extent and impact of podocyte–mesangial cell cross‐talk.

### 10.3 Podocytes and Parietal Epithelial Cells

Parietal epithelial cells (PECs) line the Bowman's capsule and are a diverse cell population with a multitude of subpopulations. During glomerulogenesis, podocytes and PECs are both derived from the metanephric mesenchyme and initially also share a phenotype. Physiologically, podocytes can influence PECs across the Bowman's space through the primary filtrate. Podocytes release exosomes into the urine, which could be used to influence PECs [399, 400], as well as certain podocyte‐derived proteins that could also be taken up by PECs from the primary urine [364]. Further communication is possible at the vascular pole where podocytes and PECs are in close proximity under physiologic conditions [2]. Certain factors hint at a dependence of PECs and podocytes on each other. Podocyte loss or depletion is associated with PEC hyperplasia [401], and PEC depletion results in transient proteinuria with focal podocyte foot process effacement [402]. Further, comprehensive single‐cell RNA‐sequencing suggests that depending on the maturity of podocytes, different feedback mechanisms towards PECs seem to exist [403]. In adult mice, for example, it has been shown that podocyte‐derived CXCL12 inhibits PEC activation [404], however, this feedback loop seems to only exist in adult mice and not in juveniles [403].

Under pathophysiologic conditions, communication between podocytes and PECs can originate from bridges formed between the capillary tuft and Bowman's capsule, which put podocytes in close contact with PECs [405]. The same is true within intraglomerular crescents, which include podocytes and PECs in direct neighborhood [406, 407]. Experimental data and mathematical 3D multiscale modeling studies [408] show that the communication between podocytes and PECs might regulate the proliferation of both cell types and possibly the regeneration of podocytes from PECs. As such, in rapidly progressive glomerulonephritis, the heparin‐binding epidermal growth factor‐like growth factor (HB‐EGF) as well as the EGF receptor are *de novo* expressed by podocytes and PECs. The proliferation of the cells and crescent formation depends on this expression [310]. Lineage tracing experiments suggest that in glomerulonephritis, podocyte regeneration occurs from renal progenitor cells located in Bowman's capsule [409], a process that can be enhanced by retinoic acid [410]. The synthesis of retinoic acid in glomeruli is thought to promote PECs (as potential renal progenitor cells, see also Section 5) to differentiate towards a podocyte phenotype [296]. Further experiments in experimental models, including lineage tracing experiments, showed that a podocyte‐specific knockdown of Krüppel‐like factor 4 (*Klf4*) causes podocyte loss and leads to pathologic PEC proliferation and FSGS [411, 412]. In these studies, Klf4 regulates transcripts involved in cell–cell communication, proliferation, migration, and ECM signaling through the STAT3 pathway. Single cell transcriptomic analysis suggests that the podocyte‐specific knockdown of Klf4 triggers the transition of PECs from the quiescent to the activated state, rather than inducing a transdifferentiation of podocytes to PECs or of PECs to podocytes [412]. Further single cell analyses in an experimental model of anti‐glomerular basement membrane identified a transient high podocyte expression of ligands, with their corresponding receptors expressed in PECs. Such podocyte‐expressed ligands included macrophage migration inhibitory factor (Mif), interleukin 34, CXCL12, colony stimulating factor 1 (Csf1), and TGF‐beta2. Substantiating these podocyte‐expressed ligands as potential cross‐talk routes with PECs, the inhibition of Mif or Csf1 improved kidney function and reduced the crescent amount [403], substantiating a podocyte‐PEC cross‐talk in the disease setting.

All the above‐mentioned studies strongly suggest that specific crosstalk between podocytes and PECs exists, which especially targets the proliferative properties of the cells. Further studies are needed to really understand how the different glomerular cell types influence each other in health.

## 11 Podocyte Metabolism

### 11.1 Overview

**FIGURE 16:**
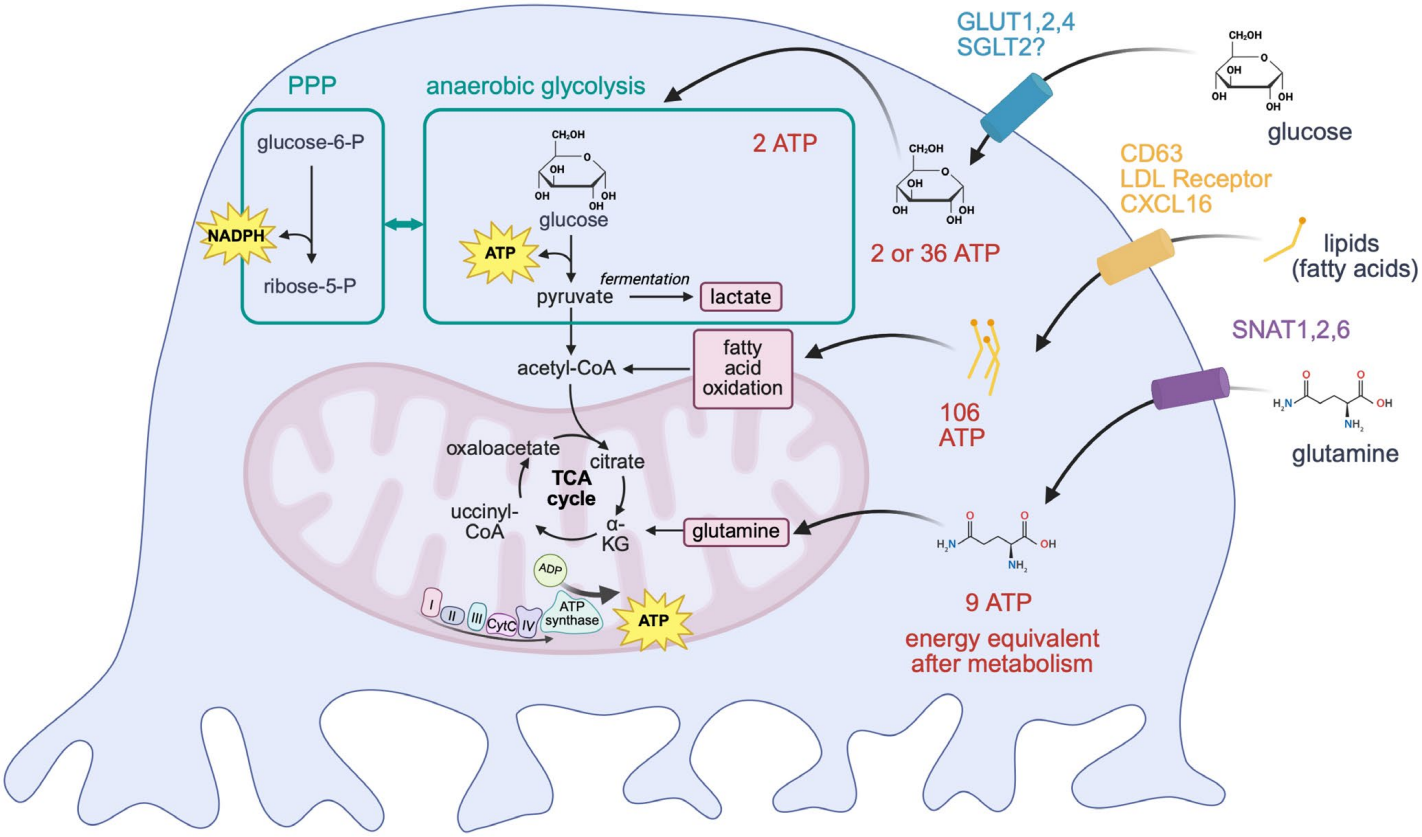
Podocyte metabolism. Our knowledge about what podocytes metabolize for their homeostatic energy provision is scarce. The scheme provides an overview of the different energy sources, uptake pathways, and our current knowledge of key podocyte metabolic principles. Immortalized human podocytes show a high expression of genes essential for the pentose phosphate pathway (PPP), which would represent an addition to anaerobic glycolysis [413] for energy production from glucose as fuel. ADP = adenosine diphosphate; ATP = adenosine triphosphate; CD63 = tetraspanin; CoA = coenzyme A; CXCL16 = SR‐PSOX (scavenger receptor that binds phosphatidylserine and oxidized lipoprotein); glucose‐6‐*P* = glucose‐6‐phosphate; GLUT = SLC2A type glucose transporter; LDL = low‐density lipoprotein; ribose‐5‐*P* = ribose‐5‐phosphate; SGLT2 = sodium/glucose cotransporter 2; SNAT = sodium‐coupled neutral amino acid transporter; TCA = tricarboxylic acid cycle, also known as the Krebs cycle or the citric acid cycle.

It is likely that the physiologic energy availability required by podocytes as terminally differentiated, quiescent cells is substantial to assure the maintenance of their cellular functions. But how do podocytes manage their energy metabolism? In general, the two major cellular pathways to produce energy are glycolysis and oxidative phosphorylation within mitochondria, and most cells can switch between these pathways to react to changing energy supply and demand. In the glycolytic pathway, energy is produced by the conversion of glucose into pyruvate, which is then either further processed into carbon dioxide (CO_2_) and water (H_2_O) in mitochondria (aerobic) or into lactate (anaerobic) in the cytoplasm. Depending on the NADPH demands of a cell, glucose is processed into glucose 6‐phosphate by glycolysis and then further processed by the pentose phosphate pathway (PPP) to generate nicotinamide adenine dinucleotide phosphate (NADPH) or catalyze the nonoxidative conversion of multicarbon sugars. Besides aerobic glycolysis, mitochondria can generate energy by synthesizing adenosine triphosphate (ATP) through the oxidative phosphorylation of fatty acids and glutamine (Figure 16). In brief, the mitochondrial respiratory chain required for oxidative phosphorylation is composed of four protein complexes. Complex I is the nicotinamide adenine dinucleotide hydride (NADH) dehydrogenase, complex II is the cytochrome c reductase, complex III is the cytochrome c oxidase, and complex IV is cytochrome c. This transfer of electrons and protons across the inner mitochondrial membrane generates the electrochemical gradient for ATP synthesis in complex V. Some oxygen molecules are not reduced into water during oxidative phosphorylation but form reactive oxygen species that can be converted into highly reactive radicals [414]. Mitochondrial respiration accounts for 75% of the total cellular respiration in primary podocytes [415]. Inhibition of glycolysis and of oxidative phosphorylation reduces ATP levels in podocytes, leading to the conclusion that podocytes have a very limited ability to increase oxidative phosphorylation or glycolysis, making them susceptible to energy supply dysfunctions [416].

It is scarcely investigated which precise metabolic principles are used by podocytes to ensure a sufficient energy supply, and most data are derived from either immortalized podocyte cell lines or primary cell culture podocytes.

### 11.2 Glucose Metabolism

Podocytes take up glucose through glucose transporters (GLUTs) 1, 2, and 4 [417, 418] upon insulin stimulation. Recent investigations in human immortalized podocytes suggest that podocytes also express the glucose transporter SGLT2 [419, 420] as a putative glucose uptake mechanism. The glycolytic enzyme pyruvate kinase M2 is broadly present in podocytes [421, 422], supporting the current interpretation that podocytes strongly depend on glucose for energy generation. Based on a recent study, podocytes mainly metabolize glucose through anaerobic glycolysis [423] to NADH and pyruvate, which is further processed by fermentation to lactate. Podocytes might possibly prevent the ensuing cellular acidification that originates from the accumulation of lactate during anaerobic glycolysis by the secretion of lactate into the primary urine [423]. Anaerobic glycolysis leads to an equivalent of 2 ATP per glucose molecule. Depending on the presence of oxygen, pyruvate can escape fermentation and be utilized in aerobic glycolysis in mitochondria via the tricarboxylic acid cycle (TCA, also known as the Krebs cycle or the citric acid cycle) to an equivalent of 36 ATP per glucose molecule. Podocytes increase their glycolytic activity upon exposure to glucose. However, further analysis substantiates that the cellular ATP production is significantly reduced following the inhibition of anaerobic glycolysis, while the inhibition of oxygen (O_2_)‐dependent ATP generating pathways, including fatty acid oxidation, did not reduce ATP content [423]. An addition to glycolysis is the pentose phosphate pathway (PPP), but information on how podocytes utilize the PPP is not clear. In different in vivo podocyte injury models, a strong expression of genes related to the pentose phosphate pathway is observed; the physiologic significance is, however, not clear [413], as well as why podocytes physiologically mainly retrieve their energy through anaerobic glycolysis.

The density of mitochondria, which are required for aerobic glycolysis, is lower in podocytes than in proximal tubular cells [423]. In an elegant mouse model, which allows the isolation of mitochondria from naive podocytes in situ, it could be shown that although podocytes contain fewer mitochondria than tubular cells do, podocyte mitochondria average twice the respiratory capacity of tubule mitochondria [424]. Additionally, an age‐related decline in respiration was described only in podocyte mitochondria isolated from male but not female mice [424]. Adding to the peculiarity of rodent podocytes, they appear to be little susceptible to a decrease of aerobic glycolysis as a source of ATP, as specific deletions of genes involved in mitochondrial biogenesis (peroxisome proliferator‐activated receptor‐γ coactivator 1‐alpha [Pgc‐1a]‐knockout), mitochondrial fusion and fission (dynamin‐related protein 1 [Drp1]‐knockout), or of mitochondrial (mt)DNA stability and transcription (mitochondrial transcription factor A [Tfam]‐knockout) do not result in an overt phenotype under physiological conditions [423]. In this context, it is of interest to note that in patients, mutations of mitochondrial genes result in podocyte injury, especially mutations due to a m.3243A>G mutation in tRNALeu gene [425] of the coenzyme Q10 (CoQ10) pathway [426]. The lipid molecule CoQ10, also known as ubiquinone, is present in all cell membranes and has a variety of biological functions. Besides its function as an electron carrier within the mitochondrial energy‐generating system, CoQ10 is involved in the beta‐oxidation of fatty acids, pyrimidine synthesis, detoxification of hydrogen sulfide, and protection from reactive oxygen species (ROS). CoQ10 is present in the normal diet, but at insufficient levels to supply mitochondria. Therefore, *de novo* CoQ10 biosynthesis in mitochondria is needed, a complex pathway that involves several proteins encoded by COQ genes. Currently, mutations in 10 different genes involved in the CoQ10 pathway have been reported resulting in human podocyte injury. The mechanism behind this is not clear, but is probably different from that of tubular dysfunction in patients with mitochondrial cytopathies [426]. Strikingly, primary mitochondrial cytopathies mainly affect the central nervous system and skeletal muscle, while renal involvement is limited to tubular dysfunction, most likely because the tubular system has a high energy demand to maintain electrolyte transport.

### 11.3 Amino Acid Metabolism

Comparison of the metabolite content between the glomerular and tubular kidney compartments demonstrated that high levels of amino acids and low levels of carbohydrates and lipids are present in glomeruli, while tubuli contain high levels of carbohydrates [423]. Podocytes require a constant supply of amino acids to maintain their foot processes [427, 428]. This suggests that amino acid metabolism could play an essential role for podocyte homeostasis among the other glomerular cell types. Amino acids are not only important as substrates for protein synthesis but also function as regulators of fluxes through major metabolic pathways. As such, the transport of amino acids across plasma membranes regulates the flow of nutrients into or from cells and participates in interorgan amino acid nutrition, facilitating the amino acid distribution in different cells enabling protein synthesis [429]. The sodium (Na^+^) independent system L (LAT) is a major transporter to provide cells with branched chain amino acids. Scarce evidence exists that LAT2 (SLC7A8) and LAT3 (SLC43A1) are expressed in both mouse and human glomeruli and localize to the apical membrane of podocytes [430, 431], suggesting these as possible amino acid transporters in podocytes in addition to glutamine through SNAT1 (SLC7A5), SNAT2 (SLC38A2), and SNAT6 (SLC38A6) [432].

Podocytes metabolize the amino acid glutamine by a process called glutaminolysis, which is related to the glucogenic amino acid uptake (i.e., the uptake rate of all amino acids except leucine and lysine). The metabolism of one glutamine results in the net generation of 9 ATP. The main transporters for glutamine in podocytes are the sodium‐coupled neutral amino acid transporters (SNAT)1, 2, or 6, and the uptake increases in stressed conditions through induction of SNAT3 [432]. Metabolomic studies have shown that impaired amino acid metabolism is associated with diabetic kidney disease development. Glutamine is a versatile amino acid involved in energy generation, metabolism homeostasis, cell proliferation, and apoptosis and seems to be of homeostatic importance for podocytes. As such, the liver receptor homolog‐1 (LRH‐1) is involved in amino acid metabolism as well as in lipid and glucose metabolism. LRH‐1 was shown to alleviate diabetes‐induced podocyte injury by enhancing glutaminases 2‐dependent glutamine mobilization and utilization in mitochondria [433].

### 11.4 Lipid Metabolism

Lipids are a key structural component of our biological membranes, which organize the localization and function of plasma membrane proteins, play key roles in our energy metabolism, and are potent signal transduction molecules, orchestrating intra‐ and extracellular signaling pathways. The term “lipids” comprises a plethora of 8 different categories of macromolecules, including fatty acids, glycerolipids (triglycerides as one prominent subclass), and sterols such as cholesterol and its derivatives [434]. Podocytes import various lipids, including cholesterol, sphingolipids, and free fatty acids (FFAs), which are crucial for their function. As the podocyte slit‐diaphragm is a lipid‐raft‐like structure in which essential podocyte proteins are organized in a multiprotein complex [434, 435], cellular lipid/cholesterol imbalances perturb the podocyte slit diaphragm. Especially, the import of cholesterol and sphingolipids is crucial for maintaining the structure and function of lipid rafts near the slit diaphragm [436]. In podocytes, circulating unoxidized or oxidized low‐density lipoprotein (LDL) is the major source for cholesterol uptake into cells via the LDL receptor or C‐X‐C motif ligand 16, a scavenger receptor. LDL and its receptor complexes are internalized by endocytosis and transported to the lysosome for degradation, resulting in the release of free cholesterol to the cytoplasm. Of note, a balanced cholesterol metabolism is essential for podocyte homeostasis, especially as cholesterol ester accumulation is detrimental to podocyte health. As such, the genetic loss of the endoplasmic reticulum (ER) enzyme sterol‐O‐acetyltransferase‐1 (SOAT1/ACAT1) that converts free cholesterol to cholesterol esters for storage in lipid droplets ameliorates glomerular injury. In cultured SOAT1‐deficient podocytes, protection was shown to result from decreased amounts of cholesterol esters and thereby lipid droplets, as the efflux of the accumulating free cholesterol was increased through the transmembrane protein ATP‐binding cassette transporter A1 (ABCA1) [437]. ABCA1 also mediates the efflux of free phospholipids in an ATP‐dependent manner, and ABCA1 deficiency in podocytes leads to cardiolipin (a mitochondrial‐specific phospholipid) accumulation and reduced oxygen consumption capabilities associated with alterations in the oxidative phosphorylation complexes [438]. In line, overexpression of ABCA1 or cholesterol depletion is protective in podocyte injury [439].

Podocyte lipotoxicity does not only involve the accumulation of cholesterol esters but also triglycerides [440]. Podocytes can take up triglyceride‐rich very‐low density lipoprotein (VLDL), which can lead to triglyceride accumulation, apoptosis, and glomerulosclerosis [435]. Cellular free FAs are commonly esterified to form 3 main classes of esters: triglycerides, phospholipids, and cholesterol esters, or they are transported into mitochondria for beta‐oxidation and ATP production. Generally, the metabolism of FFA through beta‐oxidation results in the *net* generation of 109 ATP per fatty acid. It is thought that podocytes metabolize FFAs mostly in the setting of metabolic reprogramming from anaerobic metabolism toward an enhanced beta‐oxidation of FAs [441]. Fatty acids are essential to form the phospholipid bilayers of the cell membranes and act as phospholipid messengers, transmitting vital intracellular signals. Podocytes import FFAs through the main receptor CD36, with saturated and monounsaturated FFAs having different effects on podocyte health [442]. Nonesterified FAs together with monounsaturated FAs account for 70%–80% of plasma free FAs. Treatment of podocytes with palmitic acid (a nonesterified saturated FA) produces an intracellular accumulation of lipid droplets and abnormal glucose and lipid metabolism in culture [440]. Contrasting the high susceptibility of podocytes to nonesterified FAs, monosaturated FAs can attenuate palmitic acid‐induced lipotoxicity. In line with this, stearoyl‐CoA desaturase, which converts nonesterified FAs into monounsaturated FAs, is upregulated in podocytes in biopsy samples from patients with diabetic kidney disease and ameliorates ER stress and podocyte injury [443]. In pathology, the intracellular accumulation of sphingolipids or metabolites in the form of ceramide, sphingosine, sphingosine 1‐phosphate (S1P), sphinganine, sphingomyelin, ceramide‐1‐phosphate (C1P) and glycosphingolipids is also observed to exacerbate podocyte dysfunction [444, 445, 446], mostly through the induction of mitochondrial damage and ROS production [435].

Podocytes form lipid droplets, which are generally regarded as a cytoprotective mechanism [434]. However, lipid droplet accumulation is frequently observed in renal biopsy samples in coincidence with oxidative stress markers [434] which lead to the suggestion that lipid droplets in podocytes might not be a sign of protection. As dynamic organelles, lipid droplets not only regulate the storage and homeostasis of cholesterol but also of triglycerides and other neutral lipids. Lipid droplets are tightly coupled to cellular metabolism and are critical to buffer the levels of toxic lipid species; they further can act as a protective reservoir for unfolded proteins and toxic aggregates. Lipid droplets therefore have the potential to attenuate cytotoxic podocyte injury by scavenging and storing the disease‐associated apolipoprotein L1 (APOL1) risk variants G1 and G2 [447]. Lipid droplets further protect mitochondria by sequestering fatty acids and thus prevent an aberrant flux of fatty acids into acylcarnitine, which is toxic to mitochondria. Acylcarnitine is a fatty acid conjugate generated at the outer membrane of mitochondria and is required for mitochondrial fatty acid uptake for beta‐oxidation. Cellular lipid droplet content also increases in conditions of high autophagic flux, where lipid droplets also function as lipid buffers to decrease lipotoxicity [434].

Podocytes regulate their lipid metabolism through the junctional adhesion molecule (JAM)‐like protein (JAML), localized at the plasma membrane of cell–cell junctions by regulating sterol regulatory element‐binding transcription factor 1 (SREBP‐1) and its target genes involved in fatty acid and cholesterol synthesis [448]. Epigenetic processes are involved in this regulation. Sirtuin 1 (SIRT1) is one member of SIRTs (class III histone deacetylase) that are highly conserved NAD^+^‐dependent deacetylases. SIRT1 not only serves as an important energy status sensor but also protects cells against metabolic stresses. SIRT1 links JAML to SREBP‐1 signaling in podocytes, regulating SREBP‐1 expression and activity [448].

Taken together, the current investigations suggest that the podocyte metabolism primarily depends on glucose, which is utilized through anaerobic glycolysis, with glutamine (supporting mitochondrial function) and lipids (influencing disease progression) also serving as important energy sources. As most of the investigations presented here were performed in cultured (primary) podocytes, further investigations are, however, required to shed light on the homeostatic energy requirements of a podocyte residing on the glomerular capillary within the primary urinary filtrate.

## 12 Podocyte Protein Degradation and Waste Management Principles

### 12.1 Overview

**FIGURE 17:**
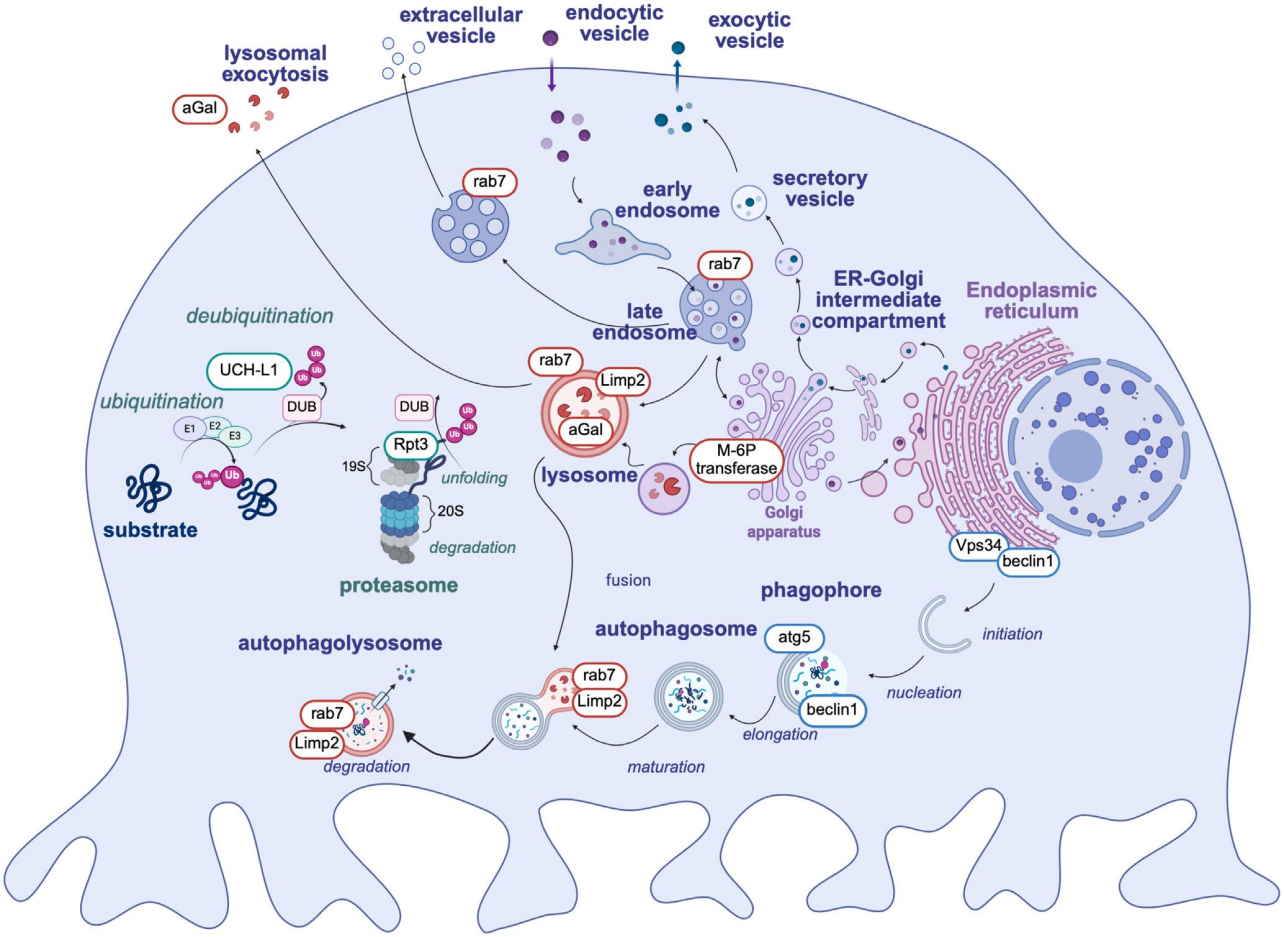
Podocyte degradation principles. Protein degradation is mainly provided by the ubiquitin proteasome system (UPS) and the autophagosomal‐lysosomal degradation pathway (ALP). Scheme localizes players of the UPS (green emboxed proteins) and ALP (red emboxed proteins for lysosomal pathway, blue emboxed proteins for autophagosomal pathway), which in genetic studies have been mutated in a podocyte‐specific or constitutive manner and exhibited a role in podocyte waste removal and homeostasis. 19S = regulatory cap; 20S = degradative core particle; aGal = alpha‐Galactosidase; atg5 = autophagy protein 5; DUB = deubiquitinating enzyme; ER = endoplasmic reticulum; Limp2 = lysosomal integral membrane protein‐2; M‐6P transferase = mannose‐6‐phosphate transferase; rab7 = ras related protein rab7; Rpt3 = regulatory particle triple‐A protein; Ub = ubiquitin; UCH‐L1 = ubiquitin C‐terminal hydrolase L1; Vps34 = vacuolar protein sorting 34.

As podocytes are terminally differentiated cells [293], they require mechanisms to remove unneeded and/or flawed proteins as well as cellular debris to prevent their accumulation during their lifetime. But how do podocytes remove their waste? The removal of proteins and of other unwanted materials is mainly mediated by two major degradation machineries, namely the autophagosome‐lysosome pathway and the ubiquitin‐proteasome system (Figure 17). The autophagosome‐lysosome pathway (ALP) primarily degrades bulk materials like proteins, polysaccharides, and complex lipids into smaller molecules [449]. Degradation in lysosomes is mediated by more than 60 different hydrolases, among them lipases, proteases, and glycosidases. Lysosomes can receive their cargo in different ways: they receive intracellular substrates through autophagy and cell surface/extracellular substrates through endocytosis [449]. Cytoplasmic components are degraded through autophagy by the formation of autophagosomes (double‐membrane vesicles) which engulf parts of the cytoplasm. These autophagosomes then fuse with lysosomes and form autolysosomes in which degradation ultimately takes place [450]. The degradation of extracellular and membrane‐bound proteins is mediated via clathrin‐mediated or clathrin‐independent endocytosis, processes involving the invagination of the plasma membrane to generate cellular uptake intermediates that then fuse with endosomes. The cargo engulfed in these endosomes is then transferred back to the plasma membrane for recycling or to lysosomes for degradation [451]. The ubiquitin‐proteasome system (UPS) on the other hand, is mainly involved in the regulation of the cell cycle, gene transcription and translation, cell survival, apoptosis, cell metabolism, protein quality control, and inflammation [452] partly by the degradation of intracellular proteins such as short‐lived regulatory proteins, as well as misfolded or simply unnecessary proteins. The UPS comprises ubiquitination, deubiquitination, and proteasomal degradation reactions. Ubiquitination/deubiquitination of target proteins via an enzyme cascade of E1, E2, E3 ligases or via deubiquitinating enzymes determines the modality and length of ubiquitin tags that then translate to protein fate, including its cellular localization, activity, or degradation. Degradation is mediated by the proteasome, a versatile structure consisting of one or two 19S caps, which recognize, bind, and deubiquitinate the target protein, and of the 20S core particle which ultimately degrades the unfolded protein into peptides through its proteolytic active beta‐subunits [452]. Over 80% of cellular proteins are thought to be degraded by the proteasome, which exists in different constitutions depending on the regulatory caps and the integrated proteolytic active beta‐subunits: compared to the constitutive proteasome, the immunoproteasome differs in its cleavage specificities as well as in its proteolytic processivity [453]. To dissect to what degree podocytes depend on which degradation system is challenging since the UPS and ALP are both tightly interconnected, visible, for example, by the compensatory upregulation of the other degradation system if one becomes impaired [454].

Within the resident cells of the glomerular convolute, podocytes in vivo express the highest levels of proteasome proteins and ubiquitin [455], which contrast with the strong lysosomal abundance exhibited by podocytes in culture [456]. Especially, the constitutive proteasome is highly abundant in podocytes [457]. Undermining the strong dependence of podocytes on the proteasomal degradation system are findings of an early and massive impact of the podocyte‐specific knockout of the 19S regulatory cap protein Rpt3 (regulatory particle triple‐A protein 3) in mice (Rpt3Δpod) on podocyte health. As such, Rpt3Δpod mice develop proteinuria at the age of 4 weeks, which is associated with podocyte loss and a decreased survival rate in comparison to littermate controls [458]. Interestingly, these mice exhibit a blockage of autophagy in podocytes of unknown origin [458]. Further, inhibition of the proteasomal system leads to a morphological disruption of the GFB, foot process broadening, and impaired endocytosis, which does not occur upon inhibition of the lysosomal system [457]. Many podocyte alterations are associated with an impairment of the UPS [238, 452].

The impact of the ALP for podocyte health is also not fully understood, despite a multitude of publications showing an involvement of autophagy [459, 460, 461] and of lysosomes [454, 462] in podocyte injury. In vivo, electron microscopic evaluations demonstrate that the podocyte cell body contains prominent lysosomes, whereas podocyte processes contain only a few organelles [462]. In some cases of ALP impairment, podocytes exhibit severe injury and loss, while in other cases podocyte injury is almost nonexistent. Investigations using a GFP‐LC3 (microtubule‐associated protein 1A/1B‐light chain 3) reporter mouse in which autophagosomes are genetically tagged suggest that podocytes exhibit high levels of autophagy [463]. Autophagy in podocytes is mainly controlled by AMP‐activated protein kinase (AMPK) and ULK1 (unc‐51 like kinase 1), rather than by mammalian target of rapamycin (mTOR). This cell‐specific mechanism of autophagy regulation is proposed to permit concurrent mTOR activity as well as high basal autophagy rates in podocytes [464]. As podocytes are sensitive to autophagosomal inhibition, it is conceived that autophagy is essential for podocyte waste removal [465]. However, the prevention of autophagosome formation through knockout of autophagy protein 5 (Atg5) in podocytes leads to a late onset phenotype with proteinuria, loss of podocytes, and age‐related glomerulosclerosis [463], suggesting a role for autophagy in podocyte aging. Interestingly, Atg5‐deficient podocytes exhibit a compensatory upregulation of the proteasome system before the onset of symptoms, whereas the proteasome system is impaired with the onset of symptoms at 8–12 months of age. These results suggest that podocyte protein maintenance relies on at least both major protein degradation machineries and that the proteasome system partly compensates for autophagy impairment.

Autophagy is directly coupled to lysosome function as one arm of the endocytic pathway but does not necessarily mirror lysosome function in glomerular cells per se, as autophagy can be altered in a lysosome‐independent way, that is, in the case of failure to form autophagosomes [466]. In line, mice with a podocyte‐specific Vps34 (vacuolar protein sorting 34) deficiency, which has been implicated in the regulation of autophagy [467], develop early onset of severe glomerulosclerosis with enlarged vacuoles and increased autophagosomes in podocytes. This phenotype seems to be the result of direct lysosomal impairment and not of defective autophagy [468]. Disruption of the final common pathway of endosomal and autophagic processing through podocyte‐specific deletion of ras‐related protein rab7 also results in early onset and severe podocytopathy [469]. Rab7 is a highly conserved GTPase that controls the homeostasis of late endo‐lysosomal and autophagic processes. Drosophila nephrocytes and cultured podocytes exhibit an accumulation of diverse vesicular structures resembling multivesicular bodies (MVBs), autophagosomes, and autoendolysosomes, as well as a reduction of slit diaphragms [469] in rab7 deficiency, suggesting an importance of the late endosomal processes in podocyte biology, especially in MVB generation. In contrast to those findings are mice displaying the lysosomal storage disorder Mucolipidosis (ML) type II and III, which do not exhibit a renal phenotype, despite severe lysosomal dysfunction due to a missorting of a plethora of lysosomal enzymes to the extracellular space instead of to lysosomes [470, 471]. Depending on the severity of lysosomal enzyme missorting (MLII is more severe than MLIII) in podocytes, different compensatory pathways are initiated. In MLIII mice, an upregulation of the UPS is thought to abrogate podocyte injury [472], similarly to Atg5Δpod mice [463]. In MLII mice, severe lysosomal impairment is counterbalanced by a downregulation of protein synthesis through the integrated stress response [472].

In summary, podocyte health strongly depends on balanced waste removal through both the UPS and the ALP; however, further analyses are required to understand the intricacies of these systems for podocyte biology. In general, the proteasome system plays a central role in homeostatic podocyte proteome regulation, especially in its function to compensate for ALP disturbances. Autophagy is regulated in a podocyte cell‐specific manner and is strongly involved in balancing proteostasis of the aging podocyte. The late endosomal/lysosomal pathway exhibits varying effects on podocyte biology. Of note, while attempting to integrate the findings of genetic ALP manipulations in mice to unravel the significance of the ALP for podocyte biology, it needs to be warranted that phenotypic ALP alterations in mice and humans are often discrepant and might not be readily translatable. For example, mutations of *SCARB2* (lysosomal integral membrane protein 2, LIMP2) in patients lead to podocyte injury and FSGS [473], whereas podocytes of mice with *Scarb2* deficiency are unaffected [474]. Another prominent example is Fabry's disease, a monogenic lysosomal storage disorder caused by mutations in the alpha‐galactosidase A (*GLA*) gene [475], which in humans relates to severe podocyte injury in part through alpha‐synuclein accumulation [476] and notch1‐mediated inflammatory and fibrogenic responses [477], but in mice does not affect podocyte biology significantly [478]. The limitations in translation are most likely due to species‐based differences in podocyte cell metabolism [479, 480].

### 12.2 Recycling

Human podocyte foot processes exhibit a high abundance of clathrin‐coated pits and multivesicular bodies [481] suggesting a high endocytic activity [482]. The podocytes propensity for endocytosis becomes prevalent during podocyte maturation and is later essential to maintain slit diaphragm integrity, as many of the involved proteins are recycled through endocytosis [483], especially nephrin and podocin [179]. Investigations of human diseases affecting the glomerular filtration barrier, as well as genetic mouse models, further emphasize the dependence of podocyte integrity on endocytosis [179]. Depending on the phosphorylation status and clustering [170], nephrin is either endocytosed via clathrin‐ or caveolin‐mediated endocytosis [484]. Using the Drosophila nephrocyte model, it was recently shown that nephrin endocytosis requires TBC1D8B, a GTPase‐activating protein for rab11 (RAB11‐GAP) of which variants cause nephrotic syndrome in patients [485]. TBC1D8B predominantly localizes to mature early and late endosomes and is required for endocytic cargo processing and degradation and for endosomal maturation [486]. Other genetic variants associated with nephrotic syndrome also impair podocyte endocytosis, such as clavesin‐1 (CLVS1), which affects clathrin‐mediated endocytosis [487], or GAPVD1 (GTPase activating protein and VPS9 domains 1) and ANKFY1 (ankyrin repeat and FYVE domain‐containing protein 1) which interact with the endosomal regulator ras‐related protein rab5 [488]. The vertebrate endocytic receptor CUBAM, which consists of three cubulin monomers complexed with a single amnionless molecule, regulates the balance between endocytosis and exocytosis in Drosophila nephrocytes [489], a process that might be conserved in murine and human podocytes, as they also express cubulin and amnionless [490, 491]. Interestingly, the exocyst complex [492], as well as apical‐basal [493] and basolateral polarity proteins [494] exhibit a strong involvement in Drosophila nephrocyte endocytosis. In some patients with nephrotic syndrome, deletions of exocyst 4 have been found, and subsequent studies in podocyte‐specific exocyst knockout mice confirmed the central role of exocyst for podocyte slit diaphragm integrity, as neph1 and nephrin were not properly localized in these mice [176]. This suggests a role of exocyst in vertebrate podocytes.

Podocyte endocytosis is an essential process in filtration barrier maintenance. In murine primary culture podocytes, a recent proteomic resolution of cargo proteins endocytosed by clathrin‐mediated endocytosis identified unique cargo proteins in murine podocytes compared to other cell types. As such, mainly receptors (transferrin receptor, cation dependent mannose‐6‐phosphate receptor), transmembrane proteins, and ECM proteins (in particular thrombospondin‐1 and fibronectin) were identified [495]. Additionally, podocytes can manage the fate of select filtered proteins through endocytosis. Global and podocyte‐specific deficiency of the neonatal Fc receptor FcRN, which mediates albumin and IgG recycling, results in subepithelial IgG accumulation in aging mice [496, 497]. In contrast to the subepithelial IgG accumulation, FcRnΔpod mice exhibit normal filtration barrier function and no glomerular albumin accumulations [497], suggesting that albumin passes the filtration barrier through podocyte transcytosis [498], as was also described for the complement membrane attack complex in autoantibody‐induced podocyte injury [499]. In line with this, using intravital microscopy, it was shown that rat podocytes endocytose albumin in a megalin‐dependent manner, while angiotensin II enhances the migration of albumin‐containing vesicles from the glomerular capillary to the apical aspect of the podocyte, which is then later released into the urinary space [498]. In cultured podocytes, albumin enters podocytes through caveolae together with the FcRN, moves along actin, and reaches the early endosome, where albumin is partly sorted for lysosomal degradation or directly transported outside the cells through exocytosis [500]. The ability of podocytes to internalize and recycle extracellular proteins through endocytosis is of potential interest for the targeted delivery of drugs to podocytes, that is, through coupling to albumin [501]. Podocyte endocytic activity seems to depend on the intracellular propensity to deal with the endocytosed cargo. As such, mice with impaired proteasome degradative capacity exhibited a decreased IgG clearance from the glomerular filtration barrier, a process partly attributed to reduced membrane dynamics leading to altered FcRN and even nephrin turnover [457].

### 12.3 Podocyte Secretory Function

The secretory function of podocytes in vivo is not thoroughly defined, and the molecular mechanisms underlying podocyte secretory function are unclear, despite our long‐lasting knowledge of an essential secretory function of podocytes for the maintenance of the glomerular endothelium via secretion of VEGF [368] or for the maintenance of the GBM via secretion of ECM proteins [502, 503, 504]. Recent work now demonstrated that beclin1 (Atg6 or becn1), a key protein in autophagy initiation, plays a role in anterograde Golgi trafficking in podocytes and VEGF secretion [505]. Podocyte‐specific deletion of beclin1 results in the display of aberrant vesicle formation in the trans‐Golgi network (TGN) of podocytes, leading to vesicle accumulation and complex disrupted patterns of intracellular vesicle trafficking and membrane dynamics. Besides VEGF, podocytes were shown to secrete active complement factor 3 (C3) and complement factor H (CFH) in culture [359], suggesting a local role for podocytes in complement regulation and immune modulation. Cultured podocytes were also shown to secrete nerve growth factor (NGF), which was proposed to play a role in the maintenance of their morphology similarly to the function of NGF in neurons [506]. Whether complement and NGF secretion from podocytes occurs in vivo and what the functionality of this secretory activity is, remains to be established.

Podocytes secrete extracellular vesicles (EVs) to the culture medium in vitro and to the urine in vivo, thus possibly contributing to intercellular communication and immune modulation. EVs are lipid bilayer membrane‐delimited, nano‐ to micro‐sized particles that appear to be released by all cell types. The term “exosome” refers to EVs from internal compartments of the cell that are released via the multivesicular body (MVB), while the term “ectosome” (also known as microvesicle, microparticle) refers to EVs from the cell surface. Numerous specialized terms have also been used to denote EVs that arise during specific cellular processes such as cell migration (“migrasomes”) or programmed cell death (‘apoptotic bodies’) [507]. Among the different types of EVs, podocytes in culture were shown to release exosomes, microvesicles, and migrasomes, based on the size of EVs detected and the expression of vesicular markers. The distinction of podocyte EVs from the totality of urinary EVs is challenging, however, EVs containing podocyte proteins such as PLA2R1 [508] WT‐1 [509], nephrin, TRPC6, INF2 [399] or podocyte‐enriched proteins such as CD35 (complement receptor 1, CR1, also present on immune cells) [510] or podocalyxin [511] (also expressed by parietal epithelial cells) have been described. Podocalyxin‐positive EVs originate from tip vesiculation of podocyte microvilli [511], whereas migrasomes represent a large EV‐type forming at tips and intersections of retraction fibers of migrating cells [512] and are positive for tetraspanin 4 in cultured podocytes [513]. It is thought that EVs derived from podocytes are involved in the transfer of proteins, mRNAs, microRNAs to recipient cells thus influencing their cellular functions [514, 515].

Recently, a novel form of podocyte microparticle was discovered, which is triggered by autoantibody binding to foot process proteins and was hence termed autoimmunoglobulin‐triggered extracellular vesicles (AIT‐EVs) (https://doi.org/10.1101/2024.04.04.588146). AIT‐EVs are formed by podocytes as a reaction to the formation of membrane‐bound immune complex aggregates, in an attempt to clear them from the subepithelial space. These vesicles not only represent an exit route for extracellular podocyte waste but also for intracellular waste, especially of proteotoxic stressed podocytes. Mechanistically, podocytes clear these membrane‐bound aggregates from the subepithelial space through translocation of basal (peroxidated) membranes with the aggregated immune complexes to urinary side foot process membranes. Following membrane budding and the formation of long (filamentous) membrane extensions, a process during which intracellular waste (dysfunctional organelles, protein aggregates) is incorporated, AIT‐EVs are released to the urine. As AIT‐EVs form at the level of foot processes but also translocate towards primary processes and the podocyte cell body, they exhibit a wide size range. AIT‐EV formation has many parallels to exopher formation in *C. elegans* neurons [516] and is likely a conserved mechanism of podocyte waste removal that links proteostasis and GFB maintenance with excretory membrane dynamics.

## 13 Podocyte Membrane Proteolysis

### 13.1 Overview

Podocytes are equipped with a unique set of surface proteins such as nephrin, neph1, podocalyxin, integrins, THSD7A, PLA2R1, and dystroglycan, to name a few, which are essential for their function. Therefore, their regulation by proteolysis is most likely of imminent importance for podocyte (patho)physiology. Ectodomain shedding is mediated by very specialized proteases, which are usually similar to their substrates, membrane‐anchored proteins. From the nearly 600 protease genes found in the human genome, several membrane‐embedded but also soluble proteases have been recognized to perform ectodomain shedding or/and subsequent cleavage of the released ectodomains. Ectodomain shedding is a key process in membrane biology, where the extracellular part of a transmembrane protein is cut off, impacting about 10% of all cell surface proteins [517]. Shedding occurs often 10–35 amino acids from the transmembrane domain [518]. Type I and type II transmembrane proteins, but also glycosylphosphatidylinositol (GPI)‐anchored proteins, are targets for ectodomain shedding [519]. Shedding is typically described to happen at the cell surface, but it may also occur in transit at the secretory route or even within endocytic vesicles, followed by the release of the ectodomain in the extracellular space. Shedding is irreversible and requires the synthesis of new proteins to replace those fragmented. Sheddases, specialized proteases, mediate this cleavage near the cell surface, releasing the extracellular domain and leaving a membrane stub that may undergo further processing. This process influences the expression and function of surface proteins and plays a role in cell adhesion, signaling, and various cellular functions like differentiation and proliferation and in diseases. The proteases involved vary, with both membrane‐bound and soluble enzymes recognized for the above‐mentioned functions. Post shedding, the membrane fragment often undergoes further cleavage by intramembrane proteases, leading to the release of cytoplasmic fragments that may be degraded or participate in signaling. This regulated intramembrane proteolysis (RIP) is a widespread and crucial cellular mechanism [520] and is mediated by gamma‐secretase or signal peptide peptidase‐like proteases [520, 521]. Our knowledge about the physiological function of podocyte‐expressed proteases that regulate podocyte membrane protein repertoire is slim. In the following, we present a brief overview of the involvement of A disintegrin and metalloproteases (ADAMs), meprins, and matrix metalloproteases (MMPs) in podocyte surface protein shedding as the most common proteases in this setting.

### 13.2 A Disintegrin and Metalloproteases (ADAM)s

**FIGURE 18:**
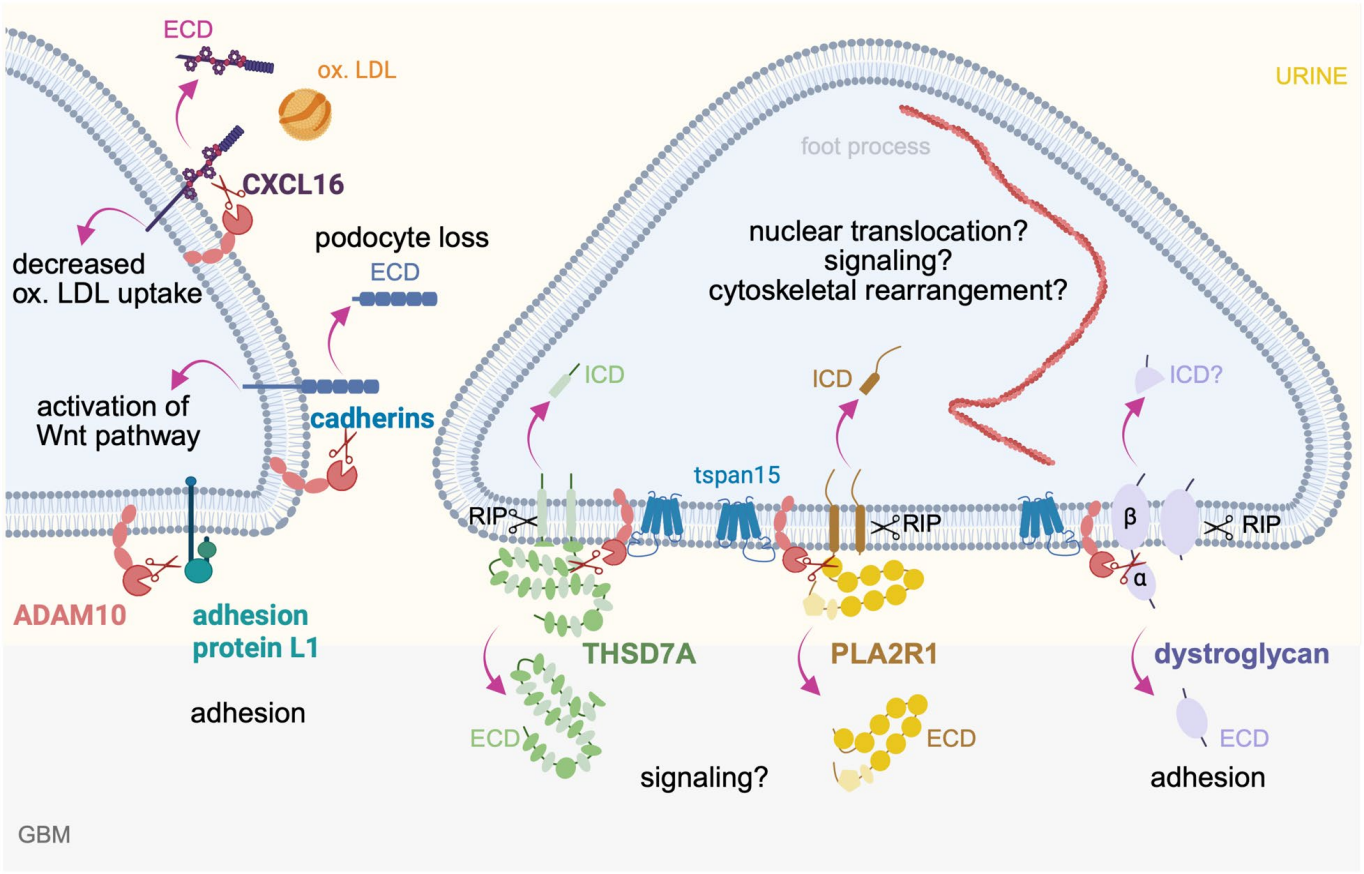
Podocyte surface proteolysis. Surface proteins of podocytes are modified by proteases of the ADAM, meprin, or MMP families. The scheme summarizes known podocyte expressed substrates of ADAM10 (and or ADAM17) and proposed sequels of surface shedding. ICD = intracellular domain; ECD = extracellular domain; ox. LDL = oxidated low‐density lipoprotein; RIP = regulated intramembrane proteolysis.

The “A disintegrin and metalloprotease” (ADAM) family of endopeptidases belong to the major players driving the proteolytic release of mainly type 1 or type 2 membrane‐embedded proteins such as growth factors, cell adhesion molecules and receptors [522, 523]. Proteomic analyses from glomerular membrane preparations identified the expression of ADAM10 and ADAM17, and to a lesser extent of ADAM15, ADAM22, ADAM9; ADAMTS1, ADAMTS5, and ADAMTSL4 in murine glomeruli. Whereas murine podocytes show a high abundance of ADAM10, ADAM17 is mainly expressed by glomerular endothelial and mesangial cells [524]. ADAM10 localizes at podocyte foot processes and to a lesser extent at the glomerular endothelium [524]. ADAM10, as a central sheddase of the notch receptor and thus activator of the notch‐dependent signaling pathways, plays a role in the development of the glomerular endothelium [525] but not in the development of podocytes [524]. In adult mice, ADAM10 is involved in the maintenance of the glomerular filtration barrier integrity (Figure 18). As such, a podocyte‐specific deletion of ADAM10 preserves the morphologic integrity of podocytes and attenuates the clinical course of disease in mice. Functionally, ADAM10‐related ectodomain shedding results in cleavage of the cell‐adhesion proteins N‐ and P‐cadherin on injured podocytes in vitro and in vivo, thus decreasing their injury‐related surface levels and favoring podocyte loss. The injury‐related expression of cadherins at the GFB is thought to compensate for nephrin loss and thus represent a “reactivation of an embryonal slit diaphragm program”, as cadherins (especially N‐cadherin) are expressed in developing podocytes [524]. In line, avians do not contain nephrin as a cell–cell adhesion protein of the mature slit diaphragm, but N‐cadherin, demonstrating, that cadherins can functionally compensate for nephrin [526]. Interestingly, loss of cadherins through ADAM10‐mediated shedding seems not only to favor podocyte loss but also result in the downstream activation of the Wnt/beta‐catenin signaling pathway, which is known to drive podocyte injury in other renal injury models [527, 528]. In line, an ADAM10‐dependent increase in Wnt‐responsive genes following antibody‐mediated podocyte injury are described in vitro and in vivo together with enhanced beta‐catenin levels and beta‐catenin translocation to podocyte nuclei, supporting a role of the Wnt‐signaling pathway in mediating podocyte cell adhesion during glomerulonephritis [528], downstream of ADAM10‐mediated N‐cadherin shedding [529]. In cultured human podocytes, ADAM10 is also involved in the shedding of CXCL16 as a scavenger of oxidized low‐density lipoprotein and in the shedding of adhesion protein L1, an important molecule for cell migration of neural and tumor cells [530]. Both shedding events are induced by exposure to proinflammatory cytokines (like Interferon‐gamma and tumor necrosis factor [TNF]‐α). Targeting of ADAM10‐mediated cleavage of CXCL16 in podocytes reduces oxidized low‐density lipoprotein uptake [531]. Recently, ADAM10 was identified as a central protease for the surface shedding of podocyte foot process proteins, including the PLA2R1 [198], THSD7A, and beta‐dystroglycan [199], substantiating a role of this protease in defining the podocyte membrane protein coverage. Considering the plethora of podocyte ADAM10 substrates, the question arises: How is the activity of this protease regulated at the podocyte surface, and is cleavage occurring homeostatically and involved/altered in podocyte injury? To this end, tetraspanins as transmembrane proteins are involved in shuttling ADAMs from the Golgi to the surface and in regulating transient interactions between molecules to promote an efficient assembly of surface proteins within specialized structures. In podocytes ADAM10 associates with tspan15 together with the foot process protein THSD7A [199]. This trimeric complex stabilizes ADAM10 at the podocyte surface and might be involved in the generation of membrane domains in which regulated surface proteolysis of podocyte proteins by ADAM10 occurs. Whether other tetraspanins play a role in orchestrating podocyte surface proteolysis is not clear, however an importance for CD151 [74, 532], and CD9 [533, 534] in podocyte physiology has been shown, partly in regulating podocyte adhesive properties [534], slowing migration and in the formation of thin arborized protrusions [532], effects that could in theory depend on ADAM [10] mediated cleavage of for example Integrins or THSD7A.

### 13.3 Meprins

Meprin is an oligomeric zinc endopeptidase of the astacin family, composed of alpha and beta subunits that are expressed separately or coordinately, either forming homo‐ or hetero‐oligomeric complexes [535, 536]. Meprin was originally identified on the apical brush‐border membrane of epithelial cells in the cortico‐medullary portion of proximal kidney tubules and the intestine, and in rat podocytes, meprin‐beta is found localized to foot processes [537]. Meprins are highly conserved among different species and are capable of cleaving a wide range of protein substrates in vitro, such as ECM components (collagen type IV, laminin, fibronectin, and nidogen [538, 539, 540]), cytokines (interleukin‐1 beta), protein kinases, growth factors, and peptide hormones [541, 542, 543]. Even though meprins act extracellularly by being either bound to the plasma membrane by transmembrane domains or secreted into the extracellular space [544, 545], no involvement in the shedding of podocyte membrane proteins has been shown till now.

### 13.4 Matrix Metallo Proteases (MMPs)

Members of the metzincin super‐family of metalloendopeptidases, notably the matrix metallo‐proteases (MMPs), are implicated in the processing of ECM proteins. In the kidney especially, MMP‐7, ‐9, and‐10 are involved in the setting of injury. The traditional classification of MMPs is based on their structure or ECM substrate specificity (reviewed in [546]) and experimental findings implementing a role of MMPs in the cleavage of podocyte surface proteins are scarce. MMP regulation and activation at the podocyte surface are complex. In cultured podocytes, this involves injury signals such as interactions with other proteins like growth factors (e.g., TGF‐β1 and basic fibroblast growth factor [FGF] [547]) and signaling pathways such as the CD40/CD154 pathway [548], a signaling pathway usually initiated by activated platelets. A physiologic role of MMPs for podocyte function still needs to be established, as they are mostly barely expressed by healthy podocytes. MMP‐7, minimally expressed by healthy podocytes, classifies as a matrilysin and degrades laminins, entactin, and other ECM components. Upregulated upon injury, secreted MMP‐7 plays (as a soluble protein) a role in the cleavage of the slit diaphragm protein nephrin [549]. Also injury‐related, the stromelysin MMP‐10 was shown to degrade ZO‐1 localized at the slit diaphragm [550] and to thus disturb podocyte integrity. MMP‐10 cannot degrade native collagen but degrades gelatins, collagen III, IV, V, elastin, fibronectin, proteoglycan, among others. Cleavage of pro‐HB‐EGF by MMP‐10 might play a role in podocyte‐parietal epithelial cell cross‐talk. The gelatinase B MMP‐9 is secreted by both mesangial cells and podocytes [551, 552] and can cleave gelatin, collagen IV, V, XI, and laminin among other ECM components. A direct podocyte target surface protein of MMP‐10 has not been identified so far. MT1‐MMP, a membrane‐anchored MMP, was shown to interact with furin and integrin alphaV at the podocyte surface, especially at the slit diaphragm. This interaction is suspected to facilitate the activation of proMT1‐MMP and thus the proteolysis of the glomerular basement membrane [553].

## 14 Podocyte Stress

### 14.1 Overview

**FIGURE 19:**
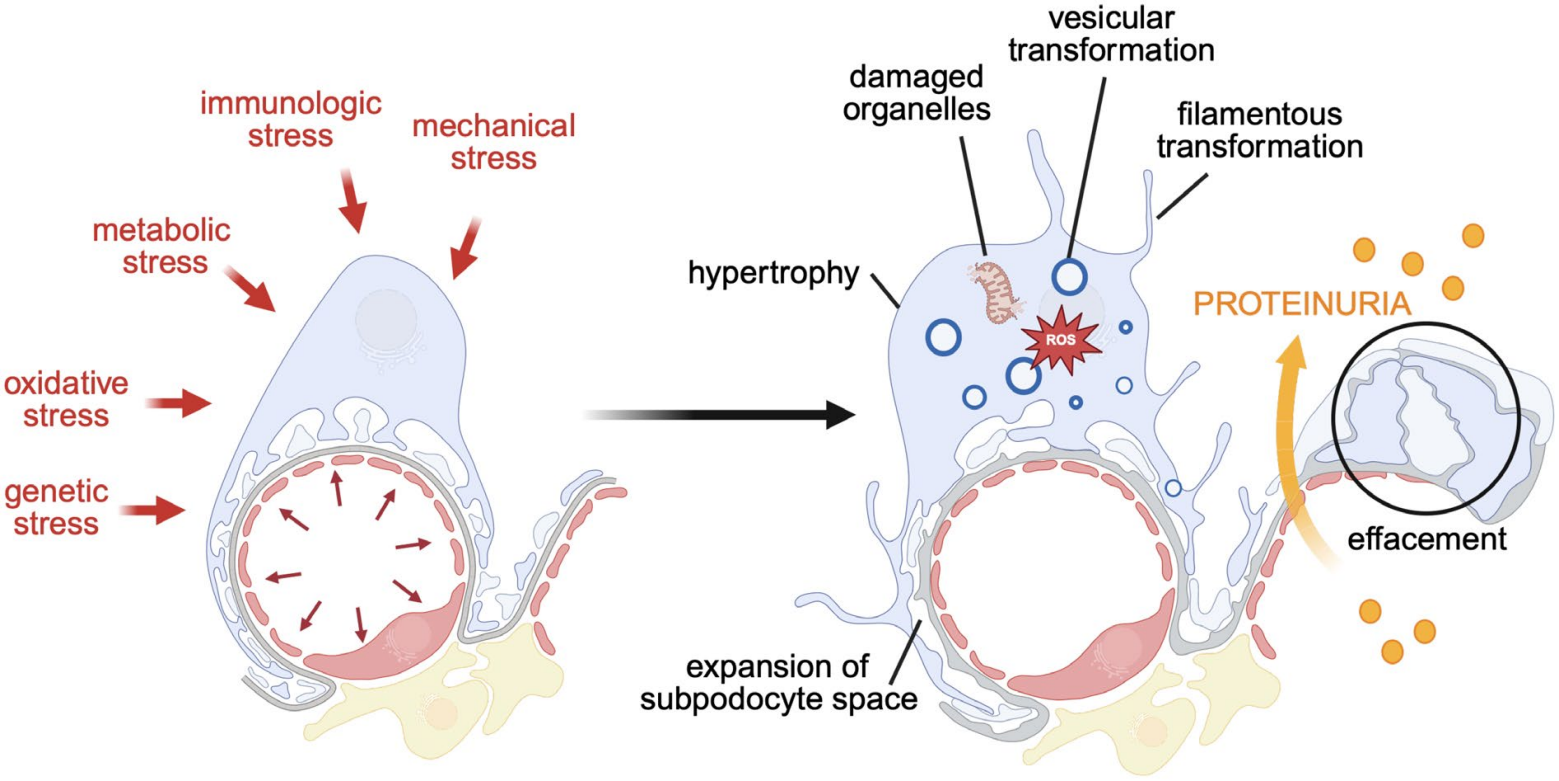
Podocyte stress. Podocytes have an intrinsic system for withstanding stresses, and they may undergo injury when the stresses exceed their compensatory capacity. The main stressors comprise genetic, oxidative, metabolic, immunologic, and mechanical injuries. Podocyte injury is characterized by ultrastructural and molecular changes. A central theme in podocyte injury is the reactivation of developmental programs. The functional consequence of podocyte injury is proteinuria, clinically visible as nephrotic syndrome. ROS = reactive oxygen species.

Podocytes, being exposed to 180 L/day of primary urinary filtrate, are the target of mechanic, metabolic, oxidative, and immunologic injury. In the recent decades, many signals mentioned in the preceding paragraphs have been unraveled that are tightly controlled to keep the podocyte healthy (i.e., VEGF [368], insulin [554], integrins [72]). It is therefore not surprising that disrupting this delicate balance can result in a dramatic loss of podocyte function [555]. Podocyte injury is mostly the result of mechanic, metabolic, immunologic, oxidative, and genetic stress. Further, reasons for (or consequences of) podocyte injury include the loss of GBM‐podocyte interactions, impaired cross‐talk to endothelial and/or mesangial cells, impairment of proteostatic principles, reactivation of developmental pathways, and podocyte ectodomain shedding, among others. Podocyte injury results in molecular and ultrastructural changes, and functionally to the development of proteinuria, ranging from albuminuria to non‐selective proteinuria (Figure 19). Clinically, nephrotic syndrome develops with the characteristics of large amounts of proteinuria (> 3.5 g per 1.73 m^2^ body surface area per day), hypoalbuminemia (< 3.5 g/dL), hyperlipidemia, and edema. Our knowledge about podocyte stress is permanently growing. As the focus of this review lies on podocyte physiology, only a brief overview about stress factors that perturb podocytes, and the molecular and structural consequences of such perturbations will be given in the following paragraphs.

### 14.2 Podocyte Stressors

Pathological mechanical stress, that is, through a rise in transcapillary pressure in the setting of glomerular hyperfiltration leads to an expansion of the subpodocyte space that lifts the podocyte cell body up [556]. Mechanical stress also arises through the enlargement/hypertrophy of the glomerular capillary tuft due to hyperfiltration. This capillary growth stretches/elongates podocyte processes leading to stress [557]. Podocytes can adapt to a certain extent to mechanical stress through hypertrophy. Underlying the importance of mechanical stress to podocyte injury is the fact that glomerular size assessed by morphometrics in patient biopsies represents the strongest predictor of long‐term clinical outcomes [558].

Metabolic podocyte stress mostly results from high glucose levels, advanced glycation end products, and insulin deficiency or resistance as present in the diabetic surrounding, as well as from dysregulated lipid metabolism as present in the diabetic or metabolic syndrome setting. The stress is thought to result from altered (either too strong or not enough) insulin signaling within podocytes [559], protein and lipid modifications/lipotoxicity through reactive oxygen species (ROS) and advanced glycation end products, and impairment of autophagy [560]. In podocytes, insulin signals through the mitogen‐activated protein kinase (MAPK) and phosphoinositide‐3‐kinase (PI3K) pathways via the insulin receptor, which leads to a direct remodeling of the actin cytoskeleton [554]. Insulin resistance in podocytes is a result of an enhanced insulin receptor degradation [561].

Oxidative stress is mediated by reactive oxygen species (ROS) which attack cellular proteins and lipids leading to their dysfunction. In podocytes, ROS are generated in response to different stressors of toxic, chemical, inflammatory, or ischemic nature. A central role of podocyte oxidative stress is attributed to the angiotensin II/aldosterone system, which induces ROS production and actin‐cytoskeletal alterations via rac1 activation. Angiotensin II binds to angiotensin II type 1 receptor expressed at podocytes, which leads to a rac1‐mediated activation of the nicotinamide adenine dinucleotide phosphate oxidase‐ROS cascade [149, 562, 563].

The causes for immunologic stress are multiple in podocytes. Podocytes are the target of the complement system through membrane attack complex (C5b‐9) formation and insertion into podocyte cell membranes [564]. Circulating cytokines that bind to podocyte‐expressed cytokine receptors such as the TGF‐β receptor and chemokine receptors CCR2 (receptor for MCP1), TNF receptor, RANK (receptor for RANKL), and Toll‐like receptors are involved in podocyte immunologic stress [565]. In the last decades, the importance of autoantibodies targeting essential podocyte proteins localized at foot processes (PLA2R1, THSD7A) [181, 200] and at the slit diaphragm (nephrin) [172] has become apparent [174].

### 14.3 Molecular and Structural Signs of Podocyte Stress

The phenotype of mature healthy podocytes is a mixture of epithelial and mesenchymal features [566]. During injury, a dedifferentiation of podocytes occurs, encompassing on the one hand the reactivation of embryonic pathways and on the other hand the loss of many epithelial features and the display of features reminiscent of specialized mesenchymal cells such as smooth muscle as well as neuronal features [567, 568]. For example, podocytes start to express proteins such as desmin, vimentin, notch, UCH‐L1 [237] on the one hand, and on the other hand lose the expression of proteins such as nephrin and podocin. They also start to express protective proteins like proteins involved in the scavenging of ROS such as sirtuin1 [569] or metallothionein [570]. Podocyte injury involves the reactivation of developmental programs such as those engaged by notch [571], wnt [527, 528, 572, 573], mTOR [574] and HIPPO [575] pathways. The overactivation, imbalance, and also impairment of these central intracellular signaling pathways involve the disruption of many essential podocyte functions, including cytoskeletal regulation, energy metabolism [576] and protein homeostasis [474]. This initiates a mostly irreversible dedifferentiation process.

Prominent morphological hallmarks of podocyte injury are foot process effacement and podocyte hypertrophy. Besides foot process effacement, ultrastructural features of injured podocytes also include cytoplasmic vacuoles, blebs, irregularities in organelles and the cell membrane, especially filamentous transformation [565]. Foot process effacement is thought to be an active process, initiated and caused by changes in the actin cytoskeleton [577]. Especially a functional imbalance among the key regulators rhoA, Cdc42, and rac1 is usually observed [578]. It is debated whether effacement per se causes proteinuria because proteinuria due to podocyte damage can occur independent of this change in shape. The relationship between podocyte foot process effacement and proteinuria is not fully understood yet [579], but might be explained by the direct action of podocytes on the GBM [23]. It is commonly accepted that effacement is the morphological manifestation of serious podocyte injury, which, as human genetic studies demonstrate, implies changes in either slit diaphragm proteins (i.e., nephrin [162] and podocin [263]), actin binding and regulating proteins (i.e., alpha‐actinin‐4 [146] and CD2AP [580]), podocyte attachment to the GBM (i.e., laminin beta2 [581] and integrin beta4 [582]), nuclear proteins (WT1 [393] and LMX1B [583]), mitochondrial [584] and lysosomal [473] components.

Podocyte hypertrophy can be adaptive in the setting of glomerular development, growth, and numerically limited podocyte depletion (up to 20%), or it can reflect a multifactorial maladaptive response of podocytes due to persistent injury‐promoting stimuli [273]. Recent findings highlight mammalian target of rapamycin (mTOR) and its downstream target, the translational repressor protein 4E‐BP1 [585] as a key regulator of both adaptive and maladaptive podocyte hypertrophy, whereby the timing, extent, and duration of mTOR activation decide whether hypertrophy is adaptive or maladaptive [574]. Inhibition of mTOR by rapamycin in the setting of adaptive hypertrophy results in proteinuria and glomerulosclerosis, whereas inhibition of mTOR in the setting of maladaptive hypertrophy could be of therapeutic benefit [586]. Besides an imbalance of mTOR signaling pathways [587, 588] podocyte hypertrophy in response to hyperglycemia and stretch has been shown to be mediated by the cyclin‐dependent kinase inhibitor p27Kip1 [589, 590]. Hypertrophy in membranous nephropathy seems to originate in part from altered protein degradation and subsequent cytoplasmic accumulation of proteins [591]. Hypertrophic podocytes may also be unable to maintain a normal foot process structure [592], increasing local shear stress, which triggers podocyte detachment [585]. Podocyte depletion is a major contributor to the development of age‐related glomerulosclerosis in humans and rodents [593, 594, 595, 596] and a decrease in podocyte number is one of the best predictors for a poor outcome in clinical diabetic kidney disease. A loss of up to 20% of podocytes is tolerated by rats [70] and mice [293] and is accompanied by mesangial cell proliferation and expansion. Segmental glomerulosclerosis ensues when 40% of podocytes are depleted and global glomerulosclerosis when the podocyte number is below 60% of normal [70].

## 15 Podocyte Death

Epithelial cells undergo various types of cell death ranging from apoptosis to non‐apoptosis related forms. It remains a mystery whether and how podocytes undergo cell death or if podocytes detach from the basement membrane when they get older and are simply shed into the urine [597]. The reason for our scarce knowledge is the fact that podocyte cell death has never been histologically seen in podocytes that reside within the glomerulus [565]. To date, the preferred theory is that dying podocytes detach from the GBM and are shed to the urine. This theory is supported by the occasional finding of free‐floating, still viable podocytes in Bowman's space [598]. Some of those detached cells are found in patient urine sediments and stand out as they have condensed or fragmented nuclei with apoptotic bodies and reduced volume reminiscent of a dying cell [599]. However, the exact mechanism how cell death occurs in detaching podocytes is still not clear. Of the various possibilities under consideration, apoptosis, or anoikis, and autophagy are the most likely types of cell death [600]. Characteristic signs of apoptosis are cell shrinkage, membrane blebbing, chromatin condensation, nuclear fragmentation, apoptotic bodies, and caspase 3/7/8/9 activation. As already described in Section 12.1, autophagy is particularly important for podocyte homeostasis under physiological and stress conditions [601] and dysregulation is assumed to be a possible pathway for podocyte death [602]. For example, deletion of Atg5 results in accumulated oxidized and ubiquitinated proteins, ER stress, proteinuria, and increased podocyte death [463]. Further, podocytes are thought to undergo anoikis, a special form of apoptosis in epithelial cells in response to detachment from the extracellular matrix [603] triggered by dysregulation of the integrin expression of the cell [604].

Currently highly debated is the theory of mitotic catastrophe as a cause for podocyte cell death. Mitotic catastrophe is defined as aberrant mitosis with chromosomal missegregation, aneuploidy, binucleation, micronuclei, and aberrant mitotic spindles observed in different kinds of kidney diseases [598, 602]. As terminal differentiation of mature podocytes ensues from an arrest in the mitotic phase G0, mitotic catastrophe as a cell death mechanism is a daring undertaking for podocytes. Actin is needed for mitotic spindle formation and is therefore no longer available for maintaining the adhesion of foot processes to the GBM. Sooner or later this leads to complete detachment of the podocyte [278]. Furthermore, the question arises why mature podocytes should go into mitosis at all. A possible explanation is that it is a desperate attempt to counteract podocyte injury [605]. Another observed phenomenon related to podocyte death is known as entosis, a process in which one podocyte is engulfed or invades another [598, 602, 606]. Adhesion proteins and actomyosin as well as autophagy‐related proteins like LC3 and the Atg family play a role in this form of cell cannibalism [602]. Further cell death theories are that podocytes undergo (1) necroptosis which leads to cell swelling, membrane rupture and loss of organelles, or (2) podoptosis with cytoplasmic vacuolization, endoplasmic reticulum stress, and p53‐activation, or (3) pyroptosis associated with cell swelling, membrane rupture, DNA condensation and fragmentation, and caspase‐1/4/5/11 activation [602]. However, precise data substantiating one definite or preferential form of podocyte cell death are lacking. Altogether, the detachment of podocytes from the GBM most likely represents the initiating event in the podocyte death cascade, the end of which remains challenging to be determined, as dying podocytes are very difficult to specifically detect within the urine. Experimental data in mice suggest a vicious cycle of podocyte loss, a so‐called ‘domino effect’. As such, loss of only a subpopulation of podocytes in human CD25 chimeric mice, which represents a receptor for the immunotoxin LMB2 (lipid‐mediated binding protein 2), results in severe podocyte injury if mice are exposed to LMB2 [607]. Severe podocyte injury includes the (expected) CD25‐positive podocytes as well as of the (unexpected) CD25‐negative podocytes. The domino effect might be explained by alterations of the podocyte‐endothelial communication by the initially injured podocytes (see also Section 10.1) that then affects other podocytes leading to progressive podocyte loss.

## 16 Experimental Models to Study Podocytes

**FIGURE 20:**
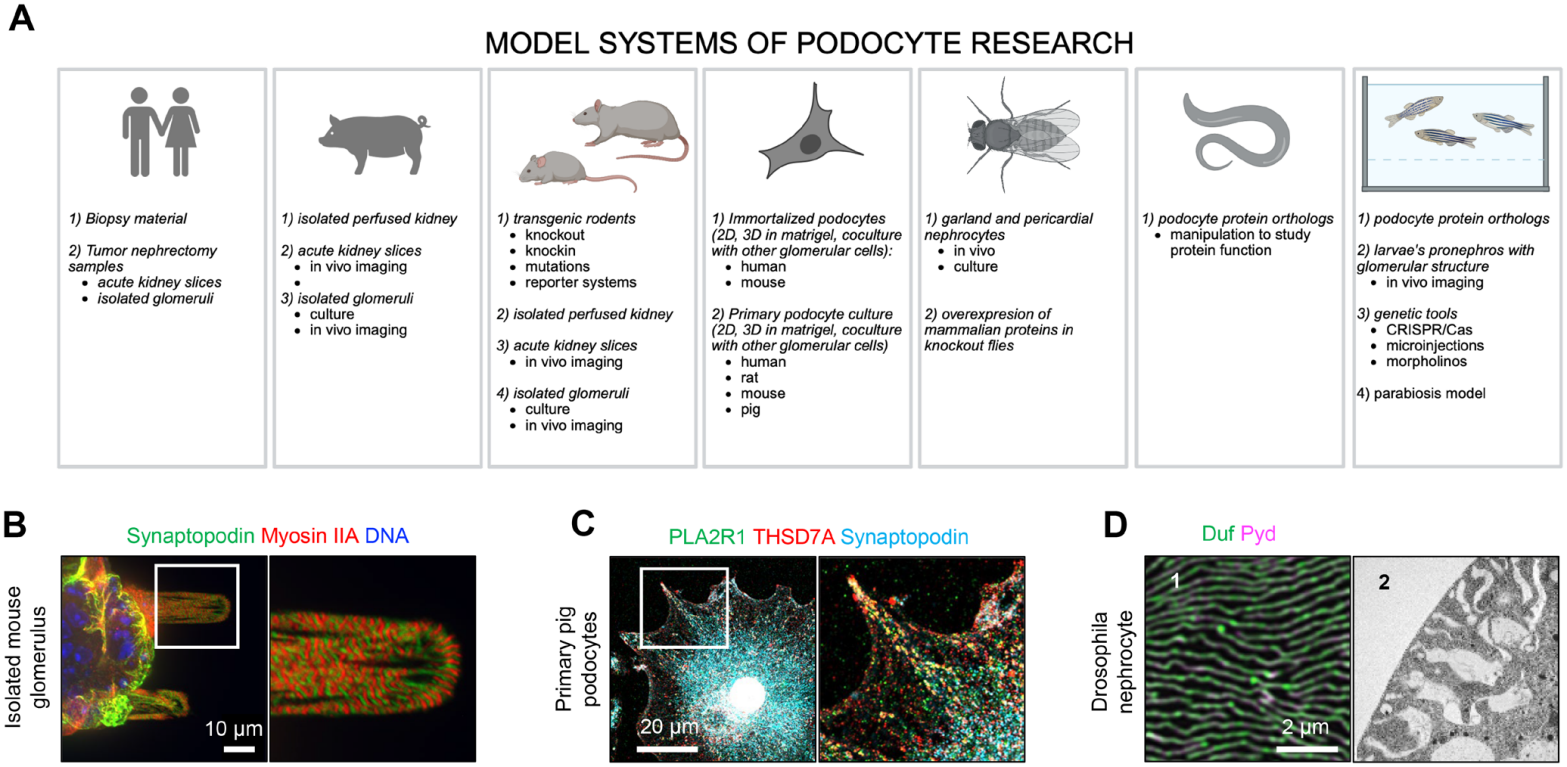
Summary of the most frequently used podocyte model systems in research. (A) Table summarizing the most common experimental models. (B) Isolated mouse glomeruli were cultured on LAM‐521 coated hydrogel. Micrograph depicts a mat of contractile sarcomere‐like structures originating from a spreading podocyte. Micrographs: courtesy of Hani Suleiman. (C) Primary pig podocytes were stained for the mature podocyte proteins PLA2R1 (green) and THSD7A (red), which are lost upon prolonged culture. Synaptopodin (light blue) demonstrates podocyte origin of the glomerular cellular outgrow. (D) Drosophila nephrocytes as a model system to study principles of podocyte slit diaphragm biology. *Panel 1* demonstrates the fingerprint like structure of Duf (neph1 homolog) and Pyd (ZO‐1 homolog) in garland nephrocytes. *Panel 2* demonstrates EM visualization of nephrocyte lacunae; Micrographs: courtesy of Sybille Köhler.

The interest to unravel podocyte biology has led many researchers to develop more and more creative model systems strongly differing in their advantages and disadvantages as well as strengths and limitations. To finalize this comprehensive review on the life of podocytes, this paragraph aims to provide a brief overview of common and novel model systems for studying podocyte biology that are the basis of our current perception of this unique cell (Figure 20).

### 16.1 Primary Podocyte Culture

One of the oldest methods to investigate podocyte biology is to culture isolated (decapsulated) glomeruli and to characterize the outgrowing cells. It is generally considered that the first cells to grow out of the glomerulus are of epithelial origin [608]; they could, however, be of visceral (podocyte) or parietal epithelial origin. Eight hours after taking isolated glomeruli into culture, podocyte foot processes broaden, and major processes become slenderer, and the formation of many microvilli occurs [609]. Subsequently, slit diaphragms become narrower and are often occluded by a tight junction, and structures resembling slit diaphragms displace from the basement membrane. After 2 days of culture, foot processes are completely retracted, and slit diaphragms disappear. Podocytes round up, are in contact with each other, and are found in a single layer epithelium with decreased iron staining, suggesting loss of anionic proteins on their cell surface. In principle, the early glomerular outgrown cell types can be differentiated into small polyhedral ciliated cells joined by junctional complexes, which grow in colonies, and a second very large, often multinucleated cell. Marker expression studies are required to distinguish visceral from parietal origin, as both cell types can carry features of podocytes [8]. Further, prolonged culture can lead to changes in phenotype and marker protein expression. For example, glomerular‐derived proliferating cells in the first cell culture passage exhibit a cobblestone appearance, and they express WT1 and O‐acetylated ganglioside, both specific markers of podocytes in vivo, but not synaptopodin, a marker of podocyte foot processes in vivo [610]. Four days after reaching confluency, the cobblestone cells begin to converge into large, arborized cells, which develop processes. These arborized cells exhibit WT1 and O‐acetylated ganglioside, and show a marked expression of synaptopodin, indicating that these cells possess markers of podocyte foot processes [610]. On the other hand, specific podocyte proteins, especially slit diaphragm‐related proteins such as nephrin and the basally located THSD7A, are quickly lost during glomerular outgrowth of podocytes [202]. Since visceral and parietal epithelial cells can share the expression of proteins under specific conditions [285, 295], differentiation in cultured glomerular outgrowths can be challenging. The approach to gain primary podocytes from glomerular outgrowths functions for a multitude of species such as mice, rats, humans, and pigs (Figure 20C). If podocytes express reporter proteins, a pure primary podocyte culture can be established, that is, upon FACS sorting of outgrown cells expressing the reporter protein [611]. Like other highly differentiated cells (i.e., neurons or cardiomyocytes), primary podocytes are unable to proliferate, and culturing leads to rapid growth arrest. Varying culture conditions (medium, coating of culture dishes with extracellular matrix proteins) have been attempted by many groups with the aim to induce nephrin expression in culture and to mimic the elaborate in vivo morphology of podocytes in the culture plate. For example, primary rat podocytes cultured with medium containing all‐trans‐retinoic acid and highly sulfated polysaccharides but no fetal bovine serum and cultured under high density on laminin‐coated plates [612] exhibit an elaborate morphology with primary and foot processes. Primary podocytes are the basis of many investigations, despite their limitations of being nonpolarized and of relying on a different metabolism and degradative setup compared to podocytes embracing glomerular capillaries [456, 457]. They are easily isolated from transgenic mouse models, exposed to mechanical (like stretch), toxic (like puromycin), immunologic (like autoantibodies), and metabolic (like high glucose) stressors.

### 16.2 Immortalized Podocyte Cell Culture Models

In the last decades, several podocyte cell lines of human, mouse, and rat origin [613] have been generated and are still widely used [614, 615]. To overcome the propagation difficulties of primary podocytes, the first immortalized murine podocyte cell line was pioneered [616]. Primary podocytes were isolated from the immortomouse [617], which expresses the temperature‐sensitive simian virus 40 (SV40) large tumor (T) antigen (TAg) directly. The first human podocyte cell lines were established by retroviral transduction of primary podocytes with the temperature‐sensitive SV40 large Tag [618]. These murine and human culture podocytes keep their proliferative characteristics at 33°C but are growth‐restricted upon shifting the culture temperature to 37°C, a process required for podocyte differentiation. Over 10–14 days of growth‐restriction (required for the degradation of TAg), podocytes develop cellular processes with actin filaments and microtubules reminiscent of podocytes in vivo [616, 619]. Furthermore, synaptopodin expression is induced, the proof of podocyte nature [620]. The temperature shift induces a proteostatic switch from mostly proteasomal proteins in undifferentiated podocytes to higher expression of lysosomal proteins in differentiated cells at 37°C [456]. Podocyte cell cultures are widely used to study principles of podocyte function in health and disease. They have strongly contributed to our current understanding of podocyte biology at the level of protein expression, survival, cell–cell communications, cytoskeleton regulation, effects of environmental changes and others [615]. However, there are several limitations associated with immortalized podocyte cell lines. They grow as a monolayer without any association to endothelial or mesangial cells, which are necessary for their function within the glomerulus. Most importantly, cell culture podocytes do not form slit diaphragms with neighboring cells, the hallmark of differentiated podocytes [614] nor do they show the typical strong expression of the eminent podocyte proteins such as nephrin and podocin [619]. Culture variations to overcome some of these limitations comprise transwell assays to recapitulate the filtration barrier, podocytes derived from human‐induced pluripotent stem cells [621], podocyte culture on cell‐derived decellularized matrix (DCM) [622], or three‐dimensional podocyte‐endothelial co‐culture models [623]. In this setup, podocytes adhere to the upper side of a porous membrane coated on both sides with type IV collagen, and VEGF‐matured endothelial sides adhere on the lower side. This co‐culture can be assembled with podocyte cell lines as well as with primary podocytes, extending the use to cells derived from transgenic mice [623]. Like primary podocytes, immortalized podocytes have facilitated (patho)physiologic investigations as they can be challenged and genetically modified. Further, as large amounts of podocytes can be obtained, the use of immortalized podocytes facilitates complex biochemical analyses and omics technologies and enables translational investigations when using human podocytes.

### 16.3 Caenorhabditis elegans

The nematode *Caenorhabditis elegans* ( *C. elegans* ) does not have a filtering homolog structure to the mammalian glomerulus. However, ortholog genes to neph1 and nephrin, SYG‐1 and SYG‐2 respectively, are expressed in analog structures and can therefore be used to understand the general mechanism of podocyte morphology and function [619]. Furthermore, the nematode's ortholog to podocin, MEC‐2, which is required for touch sensation in the worm, shares similar activity in the worm and in mammals: both bind cholesterol to regulate associated ion channels [33]. The short life cycle, cost‐effective keeping, and the fully mapped genome with easy manipulation possibilities make *C. elegans* a powerful tool to study cellular mechanisms, not only in kidney research [624]. However, lacking homologs to mammalian organs, *C. elegans* is of limited use for in‐depth studies on podocyte function.

### 16.4 Drosophila melanogaster

The fruit fly *Drosophila melanogaster* is another non‐mammalian model with a short life span and simple gene editing possibilities. In contrast to the nematode, it possesses podocyte‐like cells [625], the nephrocytes, which exist in two populations, namely as garland and pericardial cells. Nephrocytes form foot processes attached to a basement membrane. In between foot processes, the nephrocyte diaphragm builds part of the filtration barrier and is followed by an extensive lacunae system, where endocytosis takes place. Due to an elaborate endo/exocytotic activity, nephrocytes present with a high number of vesicles. Nephrocytes exhibit a high morphologic and molecular similarity with podocytes, as their functional roles are to filter the hemolymph and endocytose toxins and waste products. Hence, nephrocytes are an established model system to study the underlying machinery of membrane dynamics encompassing especially Duf (dneph1) and Sns (dnephrin) localization and internalization. These insect orthologs to nephrin and neph1 are involved in cell–cell recognition, adhesion, and form slit‐diaphragm‐like structures in nephrocytes with filtrating capability [626]. Sns deficiency results in loss of these slit diaphragm structures and thickening of the basement membrane, like nephrin deficiency in mammals. Nephrocytes can be targeted genetically in a cell‐specific manner by using the nephrocyte‐specific Gal4 fly strain Sns‐Gal4, which is then mated with the different RNAi lines [627]. Explanted nephrocytes enable analysis of endocytosis and filtration across the slit diaphragm‐like structure by applying fluorescent tracers [625, 628]. Performing rescue and overexpression studies of mammalian proteins in knockout animals as well as high throughput analyses due to the short lifecycle is rather simple in Drosophila, making this insect an ideal model to analyze variants of mammalian orthologs [629]. However, caution is required for direct translation to humans, as the mammalian kidney is much more complex and differently innervated than Drosophila nephrocytes.

### 16.5 Danio rerio

The zebrafish *Danio rerio* represents a highly versatile model organism for the study of podocyte biology, including functional and morphologic aspects such as glomerular filtration, ultrastructural analyses, and evaluation of podocyte response to nephrotoxic insults [630]. The zebrafish larvae's pronephros, which is composed of two bilateral pronephric ducts linked with one fused glomerulus in the midline of the larvae, is very similar to the human metanephros [631]. The glomerulus of the zebrafish pronephros displays podocytes, glomerular basement membrane, a fenestrated endothelium, and mesangial cells [631]. Podocytes form a functional slit diaphragm [632, 633]. Glomerular filtration begins as early as 48 h post‐fertilization, and a fully functioning pronephros is fully developed within 72 h post fertilization [634]. Zebrafish podocytes express the typical slit diaphragm proteins nephrin and podocin as well as podocyte proteins involved in human pathophysiology such as THSD7A [635]. Integrity of the filtration barrier can be assessed in larvae by quantifying protein lost into a small water volume in which larvae were housed for 24 h. In zebrafish, protein loss can be measured by quantifying the loss of fluorescent tracer molecules from the fish circulation [636]. The gene homology of 70% between zebrafish and human and genetic manipulation (CRISPR/Cas system, microinjections of morpholinos, DNAs, RNAs, and microRNAs) is readily done. However, some of these manipulations can have effects on the developing embryo, and observed phenotypes might not be specific to the tissue or organ of interest. Furthermore, injection of these molecules often has off‐target effects, which are sometimes difficult to identify [637]. Like in Drosophila, genetic tools have been developed that allow podocyte‐specific investigations in the zebrafish. The translucency of zebrafish larvae enables high‐end microscopical techniques such as intravital microscopy to image living biological podocyte processes [125]. Parabiosis‐based zebrafish models are feasible to generate a common circulation between two zebrafish larvae [638], thus making the zebrafish in general a valid and highly versatile model for renal (patho)physiological investigations.

### 16.6 Organoids

The above‐mentioned models provide many insights into podocytes biology, which are, however, not 100% transferable to humans, particularly in the translation of diseases. The establishment of the organoid technology from human induced pluripotent stem cells (hiPSCs) opened new avenues of basic medical research in general, but also specifically in kidney research, as kidney organoids consist of multi‐segmented nephron epithelial cells. In general, organoids are three‐dimensional (3D) miniaturized versions of organs or tissues that are derived from cells with stem potential and can self‐organize and differentiate into 3D cell masses, recapitulating the morphology and functions of their in vivo counterparts. Compared with traditional bidimensional culture, organoid culture systems have the unique advantage of conserving parental gene expression and mutation characteristics, as well as long‐term maintenance of the function and biological characteristics of the parental cells in vitro [639]. Thus, hiPSCs are induced to differentiate into primitive streak, intermediate mesoderm, and subsequently self‐organizing nephron progenitors by manipulating the Wingless related integration site (wnt), fibroblast growth factor (FGF), and transforming growth factor beta (TGF‐β) pathways [640]. Kidney organoids can nowadays also be derived from patients, healthy donors [641, 642], or from embryonic kidney progenitors [643]. As summarized recently [644], kidney organoids offer several advantages over animal models and 2D cell culture. Despite the heterogeneity of individual organoids in respect to their number of podocytes, degree of differentiation and cellular composition in part due to the lack of vascular structures and hence glomerular perfusion, the generation of functional glomerular structures with nephrin‐positive podocytes and endothelial cells was recently described [645, 646]. Additionally, kidney organoids were shown to exhibit a simple filtration ability when transplanted into mice [647], and to have a certain stress response (i.e., to gentamycin or cisplatin treatment) as seen by the expression of typical kidney injury markers like kidney injury molecule (Kim)1 or caspase‐3, showing the usability of kidney organoids for disease modeling and drug screening [648, 649]. By using CRISPR/Cas9 gene‐editing approaches (“Clustered Regularly Interspaced Short Palindromic Repeats” and “CRISPR‐associated protein 9”), hiPSCs can be used to introduce disease specific mutations and effects of rescue/treatment can be analyzed. For example, hiPSC‐derived organoids from a patient with *NPHS1* mutation transplanted into the kidney of an immunodeficient mouse induced alterations in the slit diaphragm. In a second step, again using the CRISPR/Cas9 system to introduce the correct *NPHS1* gene, they could rescue the disease phenotype [650]. An advantage of using glomeruli‐enriched organoids in podocyte research is the expression of slit diaphragm and filtration‐associated proteins, which are not observed in immortalized cell lines [651]. Podocytes derived from stepwise differentiated hiPSCs display an interdigitated pattern with gene expression of *NPHS1*, *NPHS2* and *PODXL* representing a good and reproducible model of human podocytes. Using the same protocol with hiPSCs from congenital nephrotic syndrome patients results in disruption of podocyte polarization and less expression of slit diaphragm proteins [651]. Even though organoids currently display the best in vitro model available, they lack important characteristics of the original organ. One major disadvantage is the absence of blood vessels resulting in a limited survival time when kept in culture. Transplanted in mice, organoids can get vascularized but still are limited in growth [647]. Other disadvantages are the altered cell composition and cell‐positioning due to the lack of guidance cues that orchestrate embryogenesis of the natural organ [652] and the absence of immune cells in organoids making it impossible to study immune‐mediated kidney diseases [653] and cellular cross talk. It is noteworthy to stress that research of podocyte biology using organoids is hampered by the low degree of reproducibility, not only due to differences between genotypes, cell lines, or clones, but also within a batch or between areas of the same organoid, leading to a high degree of variability between individual experiments [654]. Additionally, due to the early developmental stage of organoids, their application in kidney research is limited. Nevertheless, kidney organoids display a good in vitro model to study podocyte biology [655] and for disease modeling, especially of genetic diseases.

### 16.7 Organ On‐a‐Chip

A milestone approach to enhance vascularization and morphological maturation of kidney organoids in vitro was the use of microfluidic devices, which increases the size of organoids significantly [656]. Originally developed as an interdisciplinary approach of different technologies called “lab‐on‐a‐chip” in the 1990s, it has been specified for cell culture applications since the early 2000s, and the specification is still going on. These devices enable the controlled supply of oxygen, differently composed media to change environmental conditions (reviewed in [657]). Growing organoids on a 3D microphysical system (MPS) enables long‐term viability as well as protein function necessary for secretory mechanisms [658] or glomerular filtration by using endothelial cells and podocytes [659]. These achievements enable mimicking physiological environments and are discussed as a real alternative to animal experiments [660]. Very recently, a new glomerulus‐on‐a‐chip model was developed, showing improved podocyte differentiation under filtration flow conditions. Further, this model enables pressure‐controlled mechanical filtration, which can be used to study filtration stress as well as for drug testing [661].

### 16.8 Organotypic Slice Cultures

Organotypic slice cultures from kidneys are just establishing themselves as a new ex vivo model for kidney research. They are bridging the gap between in vitro models and animal models, which do not fully represent the human situation. Currently, several protocols for murine precision‐cut kidney slices (PCKS) are being tested with the aim to finally use human kidney slices as an ex vivo model for pathophysiology. So far, only renal fibrosis models or acute kidney injury have been published [662, 663] and very recently, a porcine model for transplant‐related ischemia–reperfusion injury was developed [664]. However, so far PCKS have not been used for glomerular disease research, most likely because it is not yet possible to keep PCKS alive and functional in culture for an extended period of time (2 days or more) [662, 663].

### 16.9 Podocyte‐Specific Genetic Modulation in Rodents

A further breakthrough in podocyte research was the establishment of a transgenic mouse line expressing cre recombinase exclusively in podocytes. Here, cre recombinase expression is driven by the 2.5‐kb fragment of the human NPHS2 promoter (*hNPHS2Cre* [180]) enabling for the first time the podocyte‐specific deletion/overexpression of genes, as well as the podocyte‐specific expression of reporters for podocyte‐fate tracking or isolation in mice. For example, podocytes can be isolated in a high efficiency and purity in a mouse model generated by crossing the *Gt(ROSA)26Sortm4(ACTB‐tdTomato,‐EGFP)Luo* ^/J^ (mG/mT) mice [665] to the *hNPHS2Cre mice* [180] which results in endogenous expression of eGFP exclusively in podocytes and tdTomato in non‐podocyte cells, enabling FACS‐based isolation of podocytes [666]. To further allow the differentiation of podocytes and non‐podocyte cells (mesangial and glomerular endothelial cells) in this context, the FACS‐based timMEP (tripartite murine mesangial cell, endothelial cell and podocytes) method was developed, which enables the bulk isolation of pure podocytes from mice without an intrinsic fluorescent reporter expression [455] in combination with glomerular endothelial and mesangial cells from the same mouse. Over the last decade, other podocyte‐specific mouse models have evolved, such as tamoxifen‐inducible cre‐recombinase expression in podocytes, which enables the manipulation of gene expression at a desired time point [667]. Transgenic mice not only allow for the study of protein expressions and their pathophysiologic consequences, but it is now also possible to perform targeted functional studies in vivo analyzed by multi‐photon microscopy [498, 668, 669]. For example, the DREADD (Designer Receptor Exclusively activated by a Designer Drug) knock‐in mouse enables the monitoring of intracellular Ca^2+^ level increases because of drug application [670].

The hNphs2.Cre (hPod.Cre) mouse has been the backbone of podocyte research despite certain limitations. Due to the random integration of the hPod.Cre transgene and the prokaryotic nucleotide sequence, cre expression is limited and might be affected by gene silencing. Hence, in 2017 a tricistronic mouse model was generated [671] by combining the expression of a codon‐improved cre recombinase [672] with the expression of a membrane‐targeted tandem dimer tdTomato (mTomato) under the control of the endogenous *Nphs2* promoter [673]. The widely used cre recombinase originates from the bacteriophage P1 [672]. In 2002, a codon improved nucleotide sequence (ciCre) adapted to eukaryotic species by minimizing the CpG (5’‐C‐phosphate‐G‐3′) content to reduce the chances of epigenetic gene silencing and altering the stop codon leading to increased cre expression was introduced [672]. To link ciCre expression to a fluorescent reporter and to ascertain exclusive expression in podocytes, a 2A‐peptide approach was used, and integrating the sequences into the endogenous *Nphs2* locus by gene targeting was used [671]. The 2A‐peptides are 19 amino acid–long viral sequences that induce a discontinuity in the translation process at glycine 18 and proline 19 [674, 675]. Consequently, the first peptide is released from the ribosome with a C‐terminal 2A‐tag [674]. At proline 19, the translation process starts again, and a second downstream product is expressed [674]. Hence, 2A‐peptides lead to the equimolar co‐expression of different proteins from a single open reading frame under the control of a single promoter [674, 675, 676].

Podocyte‐specific gene modulation common in mice has now also become possible in rats, including overexpression [677] as well as knockdown of podocyte‐genes [678]. As a model organism for podocyte research, rats have many advantages compared to mice, such as larger kidneys, easy access to isolated glomeruli using sieving techniques and easy urine collection at higher volumes, to name a few [679]. The isolation of murine glomeruli in good purity is challenging and the best approach till now is isolation using Dynabeads. Perfusing either through the heart or the kidney artery, these 4.5 μm diameter magnetic beads accumulate in the glomerular vessels, making it possible to isolate glomeruli after tissue digestion with collagenase using a magnet. Isolated glomeruli are intact and of high purity and can be used for transcript profiling, proteomic analysis, or primary podocyte cell culture [680]. The Dynabead method is nowadays widely used for podocyte research for many different aspects.

## 17 Closing Remarks and Outlook

Podocytes are indispensable for maintaining the integrity of the glomerular filtration barrier, with their complex structure and intricate biological functions underscoring their vulnerability to injury. Disruptions to podocyte homeostasis contribute significantly to the pathogenesis of various glomerular diseases. Advances in our understanding of podocyte biology, particularly regarding cytoskeletal regulation, signaling pathways, and interactions with the microenvironment, have laid the groundwork for innovative therapeutic approaches, and further in‐depth investigations in in vivo settings are warranted, especially in the fields concerning the principles of podocyte metabolism, waste removal, and loss. Looking ahead, the integration of omics technologies, advanced imaging, and single‐cell analyses holds the promise of uncovering novel biological principles, molecular targets, and biomarkers for podocyte‐related diseases. Bridging the gap between bench and bedside will require multidisciplinary collaboration and the development of targeted, patient‐specific investigations and interventions. As we continue to unravel the complexities of podocyte biology, the potential to transform the diagnosis, treatment, and prevention of kidney diseases becomes increasingly tangible.

## Author Contributions

**Desiree Loreth:** writing – original draft, writing – review and editing. **Wiebke Sachs:** writing – original draft, writing – review and editing. **Catherine Meyer‐Schwesinger:** conceptualization, validation, formal analysis, supervision, visualization, writing – original draft, writing – review and editing.

## Disclosure

The authors have nothing to report.

## Conflicts of Interest

The authors declare no conflicts of interest. The material is conform with good publishing practice in physiology [681].

## Data Availability

Data sharing not applicable to this article as no datasets were generated or analysed during the current study.
